# Identification of novel genetic factors that regulate c-di-AMP production in *Staphylococcus aureus* using a riboswitch-based biosensor

**DOI:** 10.1128/msphere.00321-24

**Published:** 2024-09-17

**Authors:** Igor Kviatkovski, Qiyun Zhong, Sanika Vaidya, Angelika Gründling

**Affiliations:** 1 Section of Molecular Microbiology and Centre for Bacterial Resistance Biology, Imperial College London, London, United Kingdom; The University of Iowa, Iowa City, Iowa, USA

**Keywords:** *Staphylococcus aureus*, biosensors, cyclic nucleotides

## Abstract

Nucleotide secondary messengers regulate various processes in bacteria allowing them to rapidly respond to changes in environmental conditions. c-di-AMP is an essential second messenger required for the growth of the human pathogen *Staphylococcus aureus*, regulating potassium, osmolyte uptake, and beta-lactam resistance. Cellular concentrations of c-di-AMP are regulated by the activities of two enzymes, DacA and GdpP, which synthesize and hydrolyze c-di-AMP, respectively. Besides these, only a limited number of other factors are known to regulate c-di-AMP levels. Using a c-di-AMP biosensor consisting of the *Bacillus subtilis* c-di-AMP-binding *kimA* riboswitch and *yfp,* we were able to efficiently detect differences in cellular c-di-AMP levels in *S. aureus*. To identify novel factors that regulate c-di-AMP levels, we introduced the biosensor into a library of *S. aureus* transposon mutants. In this manner, we obtained mutants with increased c-di-AMP levels that contained insertions in *gdpP* coding for the c-di-AMP hydrolase and *ybbR* (*cdaR*) coding for a c-di-AMP cyclase regulator, thus validating our screen. We also identified two high c-di-AMP mutants with insertions upstream of the *nrdIEF* operon coding for the ribonucleotide reductase enzyme. Further analysis revealed that the insertion down-regulated *nrdIEF* expression, indicating that the enzyme is a negative regulator of c-di-AMP production. This negative regulation was dependent on *rsh*, encoding for the synthase of the endogenous GdpP inhibitor (p)ppGpp. The methods established in this work can be readily adapted for use in other bacteria to uncover genetic or environmental factors regulating c-di-AMP levels.

**IMPORTANCE:**

c-di-AMP is an important secondary messenger, produced by many bacterial species including the opportunistic pathogen *Staphylococcus aureus*. In this bacterium, c-di-AMP controls cell wall homeostasis, cell size, and osmotic balance. In addition, it has been shown that strains with high c-di-AMP levels exhibit increased resistance to beta-lactam antibiotics. Here, we developed a biosensor-based method for the rapid detection of c-di-AMP levels in *S. aureus*. We utilized the biosensor in a genetic screen for the identification of novel factors that impact cellular c-di-AMP. In this manner, we identified the ribonucleotide reductase as a novel factor altering cellular c-di-AMP levels and showed that reducing its expression leads to increased cellular c-di-AMP levels. As methicillin-resistant *S. aureus* strains are considered as a global health threat, it is important to study processes that dictate cellular c-di-AMP levels, which are associated with antibiotic resistance.

## INTRODUCTION

Secondary messengers are key elements required for an efficient adaptation of bacteria to various environmental conditions, such as soil, plant, animal, or human host environments (1, 2). In response to environmental cues, secondary messengers regulate various physiological traits important for bacterial survival such as biofilm formation, catabolite repression, motility, virulence factor production, or osmolyte transport (3–6). The human opportunistic pathogen *Staphylococcus aureus* is a highly adaptive bacterium, colonizing various niches within the host, and it can adapt to growth under adverse conditions such as high salt and high or low pH conditions (7–11). In addition, *S. aureus* is known for its ability to develop resistance to many antibiotics. Notably, methicillin-resistant *S. aureus* (MRSA) strains are of special concern, being the causative agent of up to 50% of hospital-acquired infections (12). *S. aureus* produces two nucleotide secondary messengers: the stringent response alarmone (p)ppGpp and c-di-AMP (13, 14). c-di-AMP is an essential nucleotide signal in *S. aureus*, required for growth and survival under nutrient-rich and aerobic conditions, as well as under osmotic stress (6, 15–17). Maintenance of osmotic balance and cell size control through the regulation of osmolyte and potassium uptake and export are well-studied roles of c-di-AMP in *S. aureus* and many other bacteria (6, 15, 18–22). Another important trait that was shown to be regulated by c-di-AMP is the resistance of *S. aureus* toward beta-lactam antibiotics (14, 23). *S. aureus* cells deficient in c-di-AMP production are significantly more susceptible to certain beta-lactam antibiotics, such as oxacillin or penicillin G, while cells overproducing c-di-AMP are more resistant (14, 23).

Given the importance of c-di-AMP for viability, growth, and survival under different growth conditions, it is vital for bacteria to tightly regulate the cellular concentration of c-di-AMP. The two main factors that regulate c-di-AMP levels in *S. aureus* are the di-adenylate cyclase DacA (also referred to as CdaA) that synthesizes c-di-AMP and the phosphodiesterase GdpP that hydrolyses c-di-AMP into pApA, which is further degraded to ADP by the enzyme Pde2 (14, 24). A *dacA* null mutant does not produce c-di-AMP and can only be propagated under certain growth conditions (15). A *dacA_G206S_* mutant, expressing the well-characterized DacA_G206S_ variant with glycine to serine substitution close to the active site of the cyclase, produces significantly lower levels of c-di-AMP compared to the WT stain but is viable under standard growth conditions (15, 23). A mutant lacking the c-di-AMP hydrolase enzyme GdpP produces drastically elevated levels of c-di-AMP. These changes in c-di-AMP levels significantly affect the physiology of the cells, impacting viability, antibiotic resistance, cell size, and cell wall homeostasis (14, 15, 23).

Besides GdpP and DacA, not many other factors are known to control c-di-AMP levels in *S. aureus*. It is known that two proteins encoded by genes in the same operon as *dacA*, the peptidoglycan precursor synthesis enzyme GlmM and the membrane-anchored protein YbbR (also referred to as CdaR), directly interact with the DacA enzyme and regulate c-di-AMP production (25–28). The stringent response alarmone (p)ppGpp has been shown to bind to the GdpP phosphodiesterase and inhibit its activity (29). In addition, glutamine and ammonium have been shown to inhibit c-di-AMP production in *S. aureus,* through a mechanism yet to be revealed (30). Recently, it has also been shown that purine metabolism is tightly linked with the c-di-AMP signaling network, as strains with a mutation in the purine biosynthesis, metabolism, and transport genes *purF, nupG,* and *deoD* have reduced c-di-AMP levels (31, 32). However, other factors, either genetic or environmental, which regulate c-di-AMP production in *S. aureus,* are not known.

To identify novel factors regulating cellular c-di-AMP levels, a rapid real-time method for the detection of cellular c-di-AMP levels is required. Currently used methods for the quantification of c-di-AMP in *S. aureus* include a mass spectrometry approach or a competitive enzyme-linked immunosorbent assay (ELISA) (14, 24, 33). These methods are time- and resource-consuming and not suitable for high-throughput screening. Shortly after the discovery of c-di-AMP, a riboswitch that specifically and selectively binds c-di-AMP was characterized (34). Upon the binding of c-di-AMP, the RNA riboswitch changes its secondary structure and in this manner affects transcription of the downstream genes, which in the case of the c-di-AMP riboswitch predominantly code for potassium, osmolyte, or amino acid transporters (34–39). Based on the rapid detection of c-di-AMP by RNA riboswitches, various reporters have been developed to determine c-di-AMP levels, whereby either a gene coding for a fluorescence protein or a dye-binding aptamer was fused downstream of the c-di-AMP riboswitch (34, 40, 41). c-di-AMP riboswitch-based biosensors enabled the *in vivo* measurements of c-di-AMP dynamics and have also been used to study differences in c-di-AMP levels between different clinical strains (40, 42).

Here, we constructed and characterized a riboswitch-based c-di-AMP biosensor for *S. aureus* by placing the *kimA* riboswitch from *Bacillus subtilis* upstream of the *yfp* reporter gene coding for the yellow fluorescence protein. Using WT, *gdpP* (high c-di-AMP level), and *dacA_G206S_* (low c-di-AMP level) *S. aureus* strains, we showed that the *kimA-yfp* biosensor can be used to detect changes in cellular c-di-AMP levels. We then introduced the biosensor plasmid into a *S. aureus* transposon mutant library and performed a screen to identify *S. aureus* mutants with altered c-di-AMP production. Using this approach, we identified known as well as novel factors including the ribonucleotide reductase (RNR) enzyme NrdEF that impact cellular c-di-AMP levels. Thus, we have established an effective high-throughput method using a c-di-AMP riboswitch biosensor to discover novel factors that affect c-di-AMP production in *S. aureus*.

## RESULTS

### Differences in c-di-AMP levels can be detected in live *S. aureus* cells using a *B. subtilis kimA* riboswitch-based biosensor

**Fig 1:**
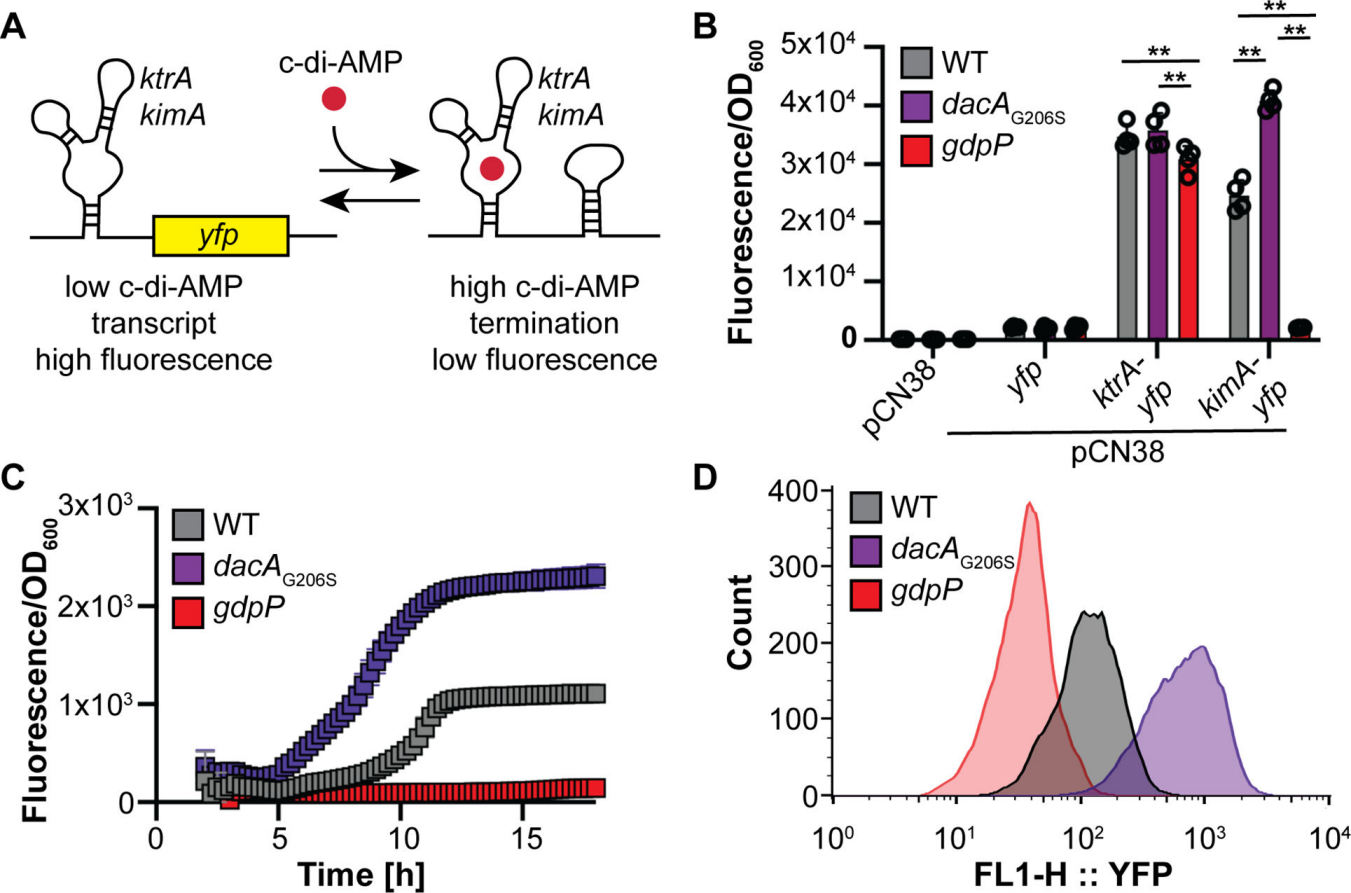
Characterization of the c-di-AMP biosensor plasmids pCN38-*kimA-yfp* and pCN38-*ktrA-yfp* in *S. aureus*. (**A**) Schematic representation of the c-di-AMP biosensor constructs. In the c-di-AMP biosensor plasmids, the *B. subtilis* c-di-AMP-binding *ktrA* or *kimA* riboswitch region is placed upstream of the *yfp* gene coding for the yellow fluorescence protein. At low c-di-AMP levels, the riboswitch is in the unbound state, allowing transcription of the *yfp* gene and giving a high fluorescence signal. At high c-di-AMP levels, the riboswitch is in the bound state, resulting in the formation of a transcription terminator and a low fluorescence signal. (**B**) End point fluorescence measurements. The WT *S. aureus* strain TM283 and isogenic *dacA_G206S_* and *gdpP* mutant strains containing the pCN38 or pCN38-*yfp* control plasmids or the pCN38-*ktrA-yfp* or pCN38-*kimA-yfp* biosensor plasmids were grown in a TSB medium in 96-well plates. After 18 h of growth, fluorescence and OD_600_ reading were taken, and the average and standard deviation of the normalized fluorescence (fluorescence/OD_600_) from four independent cultures were calculated and plotted. A two-way ANOVA followed by Tukey’s *post hoc* test was used to identify statistically significant differences between WT and mutant strains. ** indicates *P*-value <0.01. (**C**) Kinetic fluorescence measurements. The WT *S. aureus* strain TM283 and isogenic *dacA_G206S_* and *gdpP* mutant strains containing the pCN38-*kimA-yfp* biosensor plasmids were grown for 18 h in a plate reader, and fluorescence and OD_600_ readings were taken every 15 min. The average value and standard deviation of the normalized fluorescence (fluorescence/OD_600_) from six independent cultures were calculated and plotted for each time point. (**D**) FACS analyses of *S. aureus* cells containing the pCN38-*kimA-yfp* biosensor. Ten thousand cells of WT TM283, *dacA_G206S_,* and *gdpP* mutant strains containing the pCN38-*kimA-yfp* biosensor plasmid were analyzed by FACS, and the fluorescence intensity per cell was plotted. One representative result of three experiments is shown.

To establish a rapid method for the detection of cellular c-di-AMP levels in live *S. aureus* cells, we first created two c-di-AMP biosensor constructs containing the *B. subtilis ktrA* or *kimA* c-di-AMP-binding riboswitch regions. In these biosensors, the *ktrA* or *kimA* riboswitch region was placed upstream of the *yfp* gene coding for the yellow fluorescence protein and expressed from a constitutive modified spac promoter (Fig. 1A). Binding of c-di-AMP to the riboswitch region will result in the formation of a stem-loop structure and termination of transcription. Hence, if the biosensor is functional, high fluorescence should be detected in cells producing low levels of c-di-AMP, and low fluorescence should be detected in cells with high c-di-AMP levels (Fig. 1A). For the expression of the biosensors in *S. aureus,* plasmids pCN38-*ktrA-yfp* and pCN38-*kimA-yfp* containing these biosensor constructs were introduced into the *S. aureus* WT strain TM283 as well as isogenic *dacA_G206S_* (low c-di-AMP level) and *gdpP* (high c-di-AMP level) mutant strains. As additional controls, the empty vector pCN38 and plasmid pCN38-*yfp*, containing the promoter-*yfp* fragment but containing a large deletion in the *ktrA* riboswitch region, were also introduced into the same *S. aureus* strains. The *S. aureus* strains containing the control and c-di-AMP biosensor plasmids were grown for 18 h in a tryptic soy broth (TSB) medium, and at this point, fluorescence and optical density at 600 nm (OD_600_) readings were taken, and normalized fluorescence values (fluorescence/OD_600_) were calculated (Fig. 1B). All three strains (WT, *dacA*_G206S_, and *gdpP*) containing the empty vector pCN38 or the pCN38-*yfp* plasmid showed low normalized fluorescence values with no significant differences between the strains (Fig. 1B). All three *S*. *aureus* strains containing the pCN38-*ktrA-yfp* plasmid showed high normalized fluorescence readings. No difference in the normalized fluorescence value was detected between the low c-di-AMP strain (*dacA*_G206S_ mutant) and the WT strain, indicating that the *ktrA* riboswitch is not suitable for detecting changes in cellular c-di-AMP levels in *S. aureus*. On the other hand, clear differences in the normalized fluorescence values were observed between strains containing the pCN38-*kimA-yfp* biosensor plasmid. As expected for a functional c-di-AMP biosensor, significantly higher normalized fluorescence values were detected in the low c-di-AMP *dacA*_G206S_ mutant and lower values in the high c-di-AMP *gdpP* mutant compared to the WT strain (Fig. 1B). Similar results were obtained when using pCN34e-derived control and biosensor plasmids pCN34e-*ktrA-yfp* and pCN34e-*kimA-yfp* in a second MRSA strain background LAC* (Fig. S1A). The pCN34e-derived plasmids were also used for a microscopy experiment. Consistent with the fluorescence plate reader data, all strains containing the empty pCN34e vector or the pCN34e-*yfp* vector were not or only very weakly fluorescent (Fig. S1B). All strains containing the pCN34e-*ktrA-yfp* plasmid displayed high fluorescence regardless of their cellular c-di-AMP levels, while bacteria containing the pCN34e-*kimA-yfp* biosensor plasmids showed clear differences in fluorescence intensity depending on the cellular c-di-AMP levels (Fig. S1B). These data highlight that the *B. subtilis kimA* riboswitch construct is highly sensitive to changes in cellular c-di-AMP levels in *S. aureus* while the *ktrA* riboswitch construct is not able to detect these changes.

To further characterize the *kimA* biosensor, we used it to measure the c-di-AMP dynamics over time during bacterial growth. Higher normalized fluorescence values were obtained for the *dacA_G206S_* mutant compared to the WT while the normalized fluorescence values of the *gdpP* mutant only marginally increased over time (Fig. 1C). Moreover, we performed FACS analysis on these strains to characterize the ability of the *kimA* biosensor to detect differences in c-di-AMP on a single cell level. Consistent with the plate reader results, *gdpP* mutant cells produced lower fluorescence compared to the WT while the *dacA_G206S_* mutant cells produced higher fluorescence than the WT (Fig. 1D). These data show that the *kimA-yfp* biosensor can be used not only to report the c-di-AMP level on a populational level but also to monitor c-di-AMP dynamics in real time and on single cell level.

### Using the biosensor to screen for *S. aureus* transposon mutants with altered c-di-AMP levels

**Fig 2:**
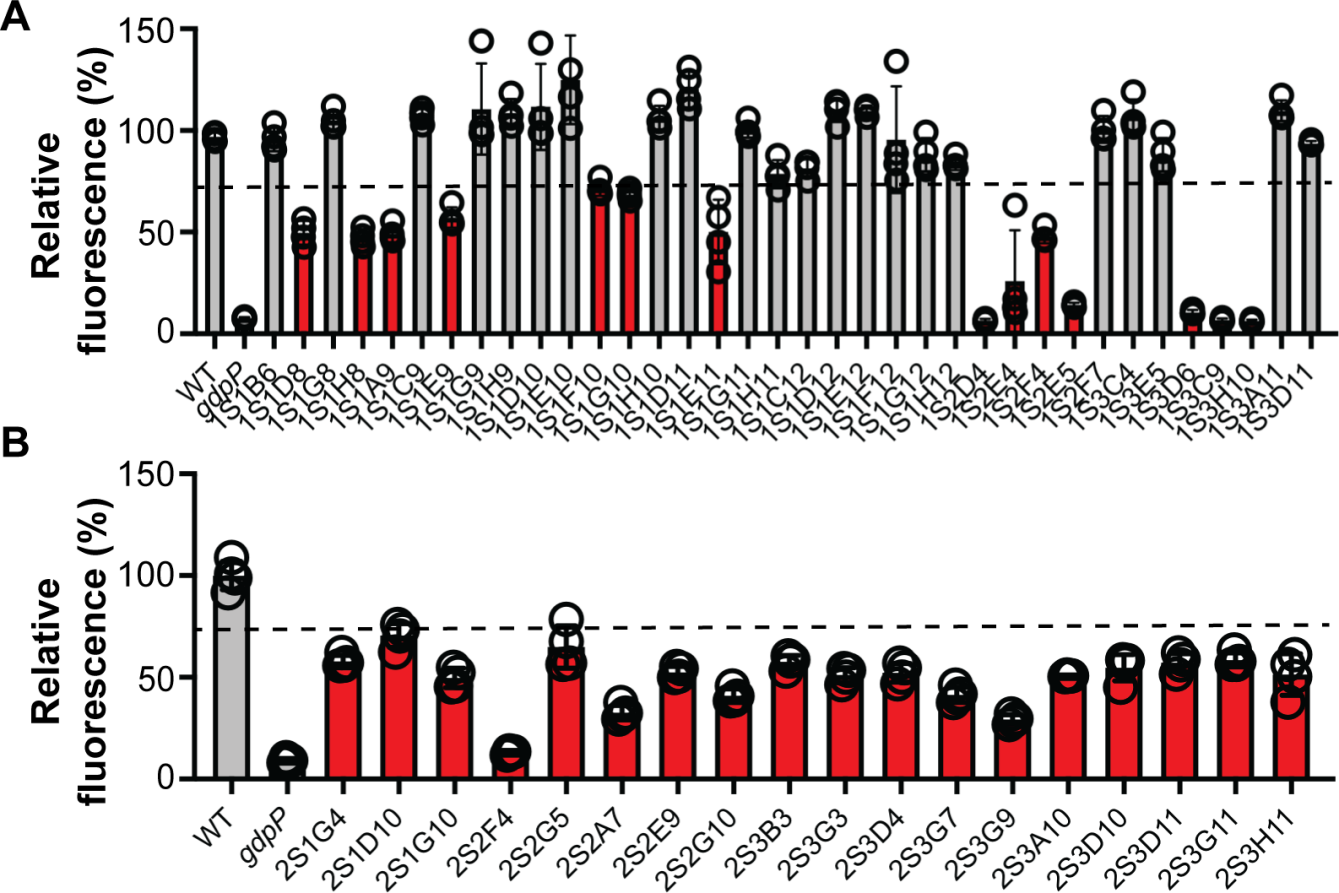
Several *S. aureus* transposon mutant strains show reduced fluorescence in the biosensor plate reader assay. (**A and B**) *S. aureus* Tn mutants as well as the WT TM283 and *gdpP* mutant control strains containing the pCN38-*kimA-yfp* biosensor plasmid were grown for 18 h in TSB in 96-well plates. At this point, fluorescence and OD_600_ measurements were taken, and the average value and standard deviation of the normalized fluorescence (fluorescence/OD_600_) from four independent cultures were calculated and plotted as relative fluorescence compared to the value obtained for the WT strain, which was set to 100%. Transposon mutant strains with relative fluorescence value of <75% compared to the WT (threshold is indicated by a dotted line) are shown in red and were further analyzed.

To identify novel factors regulating cellular c-di-AMP levels in *S. aureus,* the biosensor plasmid pCN38-*kimA-yfp* was transformed into a pooled *S. aureus* transposon (Tn) mutant library, and single transformants were picked, grown overnight in the TSB medium, and analyzed for their fluorescence production using a plate reader. Normalized fluorescence values (fluorescence/OD_600_) were calculated for all strains and compared to the value obtained for the WT TM283 strain, which was set to 100%. Strains showing relative fluorescence values of <50% or >200% compared to WT were chosen for further analysis. Of 436 Tn mutant strains analyzed from two independent transformation reactions, 55 strains exhibited altered normalized fluorescence values in this initial screen, with 52 exhibiting lower normalized fluorescence suggesting a higher c-di-AMP level, and three mutants exhibited higher normalized fluorescence, suggesting lower c-di-AMP levels (Fig. S2 and S3). These 55 strains were re-analyzed in triplicate. While none of the strains showing in the initial experiment high normalized fluorescence values could be confirmed, 32 strains had normalized fluorescence values of ≤75% compared to WT, suggesting an increase in c-di-AMP levels (Fig. 2). The transposon insertion sites of these 32 strains were mapped by arbitrary PCR. Tn insertions were found in genes coding for proteins with a variety of different functions including transporters, transcriptional regulators, and different enzymes. The data are summarized in Table 1. Three mutants had insertions in *gdpP*, which confirmed the validity of the screen. These mutants also exhibited the most significant decrease in normalized fluorescence values, suggesting that they had the highest c-di-AMP levels of all strains identified. Additionally, several other strains had insertions in genes or operons coding for proteins previously linked to the c-di-AMP signaling pathway. This included Tn insertions within the gene coding for YbbR, in an intragenic region within the operon coding for the c-di-AMP receptor PstA (DarA), as well as in *mgrA,* coding for a MarR-type transcriptional regulator, and in *SAUSA300_0091* coding for an MFS-type transporter, which in other bacteria have been shown to be involved in c-di-AMP export (Table 1). Interestingly, two strains with a large decrease in normalized fluorescence had insertions upstream of the *nrdIEF* operon, coding for the NrdEF ribonucleotide reductase and its regulator NrdI (43), which have not been associated with c-di-AMP signaling in *S. aureus* before. The Tn insertions were located at slightly different positions, indicating that these mutants were obtained independently (Table 1).

**TABLE 1:** Transposon insertion site information of 32 *S*. *aureus* strains showing reduced fluorescence in the biosensor plate reader assay

Mutant	Mutated gene	Gene/product annotation	Insertion position USA300_FPR3757	Operon
1S1D8	SAUSA300_0510	*clpC* (protease)	570046	SAUSA300_0507 (*ctsR*), SAUSA300_0508, SAUSA300_0509, SAUSA300_0510 (*clpC*)
1S1H8	Between SAUSA300_2542 and SAUSA300_2541	SAUSA300_2542: acyl-CoA ligase/acyl-CoA synthetase SAUSA300_2541: *mqo* (malate dehydrogenase)	2745828	
1S1A9	SAUSA300_2067	*glyA* (serine hydroxymethyltransferase)	2229554	SAUSA300_2068, SAUSA300_2067 (*glyA*), SAUSA300_2066 (*upp*), SAUSA300_2065 (*mnaA*)
1S1E9	SAUSA300_0091	MFS-family transporter permease	55962	
1S1F10	SAUSA300_2273	Na^+^/H^+^ antiporter family	2443962	
1S1G10	SAUSA300_1859	Acyl-CoA hydrolase	2021204	SAUSA300_1861, SAUSA300_1860 (*pepS*), SAUSA300_1859
1S1E11	SAUSA300_0945	*menD* (menaquinone synthase)	1034224	SAUSA300_0945, SAUSA300_0946 (*mend*), SAUSA300_0947, SAUSA300_0947 (*menB*)
1S2D4	SAUSA300_0014	*gdpP* (phosphodiesterase)	18539	
1S2E4	SAUSA300_1662	Aminotransferase/cysteine desulfurase	1827975	SAUSA300_1662, SAUSA300_1661, SAUSA300_1660, SAUSA300_1659, SAUSA300_1658, SAUSA300_1657 (*ackA*)
1S2F4	SAUSA300_0160	*capI* (capsular polysaccharide biosynthesis protein)	182037	*SAUSA300_0152 (capA), SAUSA300_0153 (capB), SAUSA300_0154* (*capC*)*, SAUSA300_0155 (capD), SAUSA300_0156 (capE), SAUSA300_0157 (capF), SAUSA300_0158 (capG), SAUSA300_0159 (capH), SAUSA300_0160 (capI), SAUSA300_0161 (capJ), SAUSA300_0162 (capK), SAUSA300_0163 (capL), SAUSA300_0164 (capM), SAUSA300_0165 (capN), SAUSA300_0166 (capO), SAUSA300_0167 (capP*)
1S2E5	Between SAUSA300_2037 and SAUSA300_2038	SAUSA300_2037: *cshA* (ATP-dependent RNA helicase) SAUSA300_2038: *murF* (muramyl ligase)	2202812	Between SAUSA300_2037 (*cshA*) and operon SAUSA300_2039 (*ddlA*), SAUSA300_2038 (*murF*)
1S3D6	Between SAUSA300_0714 and SAUSA300_0715	SAUSA300_0715: *nrdI* (ribonucleotide reductase stimulatory protein)	793599	Between operon SAUSA300_0714, SAUSA300_0713 and operon SAUSA300_0715 (*nrdI*), SAUSA300_0716 (*nrdE*), SAUSA300_0717 (*nrdF*)
1S3C9	SAUSA300_0014	*gdpP* (phosphodiesterase)	18536	
1S3H10	SAUSA300_0014	*gdpP* (phosphodiesterase)	18362	
2S1G4	Between SAUSA300_0672 and SAUSA300_0673	SAUSA300_0672: *mgrA* (MarR*-*family transcriptional regulator)	746785	
2S2A7	Between SAUSA300_1802 and SAUSA300_1803	SAUSA300_1802: hypothetical membrane protein	1986191	Between SAUSA300_1802 and operon SAUSA300_1803, SAUSA300_1804
2S2G10	SAUSA300_2112	*ybbR*	2288144	*SAUSA300_0113 (dacA*), *SAUSA300_0112 (ybbR*), *SAUSA300_0111 (glmM*)
2S3A10	SAUSA300_0701	Transposase	79111	SAUSA300_0698 (*pabA*), SAUSA300_0699 (*pabB*), SAUSA300_0700 (*pabC*), SAUSA300_0701, SAUSA300_0702
2S3G9	Between SAUSA300_0460 and SAUSA300_0461	SAUSA300_0460: *pstA* (*PII-like protein*) SAUSA300_0461: *holB (DNA polymerase III*)	521161	SAUSA300_0458, SAUSA300_0459 (*tmk*), SAUSA300_0460 (*pstA*), SAUSA300_0461 (*holB*), SAUSA300_0462, SAUSA300_0463, SAUSA300_0464, SAUSA300_0465, SAUSA300_0465
2S3G3	SAUSA300_2472	Drug transporter	2669525	
2S1D10	SAUSA300_2088	*luxS* (*autoinducer 2 synthase*)	2251993	
2S2G5	SAUSA300_2044	*cls* (cardiolipin synthase)	2208513	SAUSA300_2044 (*cls*), SAUSA300_2045
2S3D10	SAUSA300_0143	*phnE2* (phosphonate ABC transporter)	162810	SAUSA300_0145, SAUSA300_0144 (*phnC*), SAUSA300_0143 (*phnE2*), SAUSA300_0142 (*phnE1*)
2S3D11	Between SAUSA300_1842 and SAUSA300_1843	SAUSA300_1842: *perR* (FUR-family transcriptional regulator of ferric acid uptake)	1735884	SAUSA300_1844, SAUSA300_1843, SAUSA300_1842
2S3G7	Between SAUSA300_1585 and SAUSA300_1586	SAUSA300_1586: *aspS* (tRNA synthase)	1735885	Between SAUSA300_1585 and operon SAUSA300_1587 (*hisS),* SAUSA300_1586 (*aspS*)
2S3H11	SAUSA300_2286	Transporter	2458604	SAUSA300_2286, SAUSA300_2285
2S1G10	SAUSA300_0672	*mgrA* (MarR*-*family transcriptional regulator)	746520	
2S2E9	Between SAUSA300_2467 and SAUSA300_2468	SAUSA300_2467: acetyltransferase *srtA*	2664055	
2S2F4	Between SAUSA300_0714 and SAUSA300_0715	SAUSA300_0715: *nrdI* (ribonucleotide reductase stimulatory protein)	793572	Between operon SAUSA300_0714, SAUSA300_0713 and operon SAUSA300_0715 (*nrdI*), SAUSA300_0716 (*nrdE*), SAUSA300_0717 (*nrdF*)
2S3D4	SAUSA300_2553	*sycG* (syroheme synthase)	2761481	SAUSA300_2554 (*cysJ*), SAUSA300_2553 (*cysG*)
2S3G11	SAUSA300_2160	MerR*-family transcriptional regulator*	2337567	SAUSA300_2160, SAUSA300_2159
2S3B3	SAUSA300_2448	Putative membrane protein	2643932	SAUSA300_2448, SAUSA300_2447

### Confirmation of the effect of the Tn insertions on c-di-AMP production

**Fig 3:**
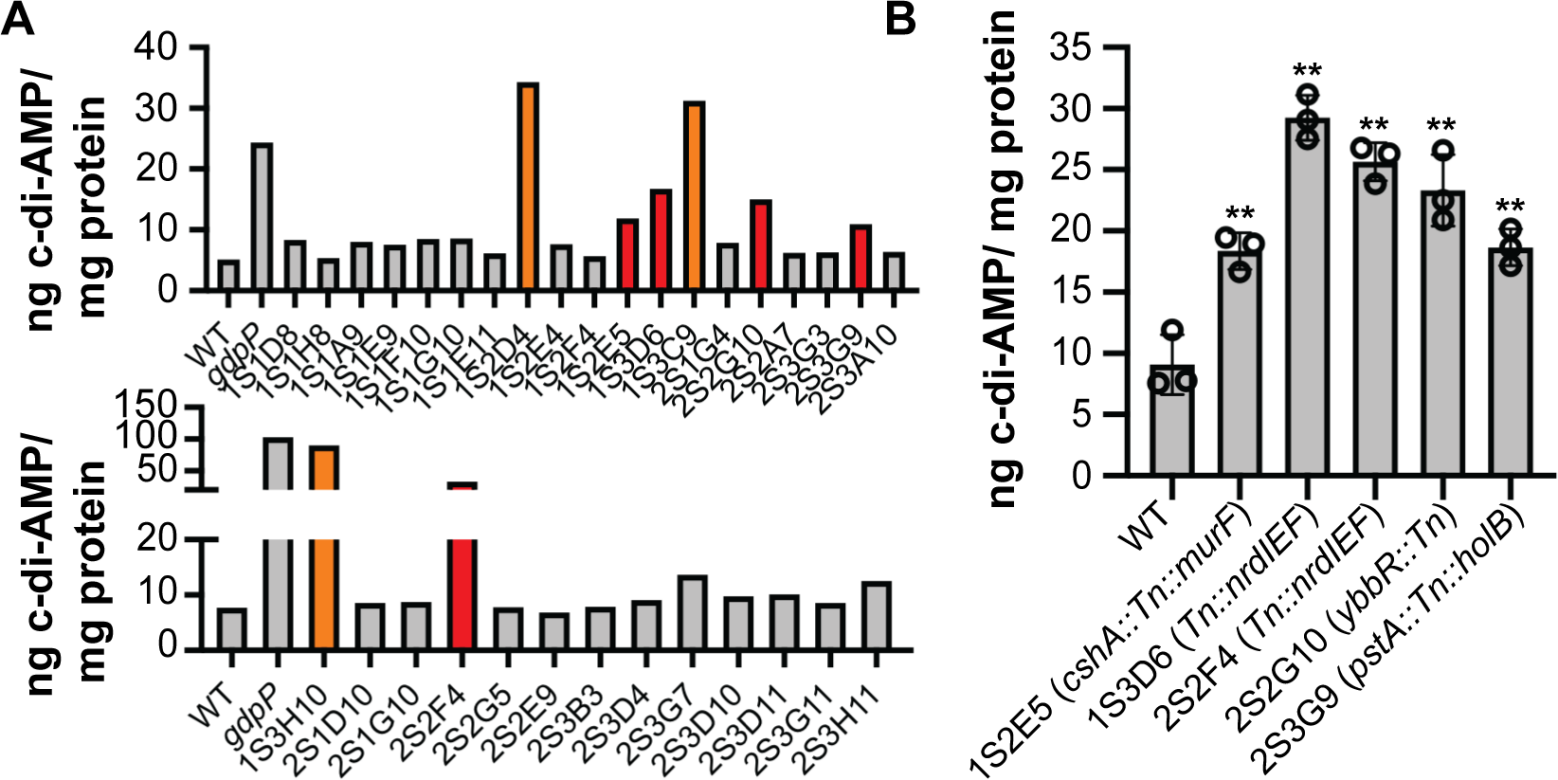
Increased c-di-AMP amounts are detected in the culture supernatant of several *S. aureus* transposon mutant strains. (**A and B**) Competitive ELISA to determine c-di-AMP levels in the culture supernatant fraction. (**A**) WT TM283, the *gdpP* mutant control strain, and the 32 transposon mutants showing decreased fluorescence in the biosensor assay (see Table 1) were grown overnight in CDM lacking NH_3_. The next day, culture supernatant fractions were prepared, and c-di-AMP levels were determined using a competitive ELISA. Transposon mutants with greater than twofold higher c-di-AMP amounts in the supernatant fraction compared to the WT strain are highlighted in color. Three strains with transposon insertions in *gdpP* are shown in orange, and five strains with transposon insertions in other genomic regions are shown in red. This initial assay was performed with a single replicate, but strains yielding high c-di-AMP levels and highlighted in red with transposon insertions in genomic regions other than *gdpP* were further analyzed. (**B**) Same as in (**A**) but using three biological replicates of WT TM283 and the five *S*. *aureus* transposon mutant strains highlighted in red in panel (A) and yielding high c-di-AMP levels. The transposon insertion site in specific genes or genomic regions is indicated on the *X*-axis label. The average and standard deviation of the c-di-AMP levels in the supernatant fraction of the three replicate samples are plotted, and a one-way ANOVA followed by Dunnett’s *post hoc* test was used to identify statistically significant differences between WT and the mutant strains. ** indicates *P*-value <0.01.

To assess if the decrease in fluorescence using the biosensor is indeed due to an increase in c-di-AMP production, cellular c-di-AMP levels were measured for the WT TM283 strain, the *gpdP* mutant control strain, and the 32 mutant strains following growth in the TSB medium using a well-established competitive ELISA method (33). In *Listeria monocytogenes* and group B *Streptococcus*, c-di-AMP has been shown to be secreted from the cells (44, 45). Hence, we also measured c-di-AMP levels in the supernatant fraction following growth in a chemically defined medium (CDM). Given the large number of strains analyzed, the initial ELISAs were performed using only a single biological replicate and three technical replicates for each strain. Although none of the strains other than *gdpP* Tn insertion mutants showed clear differences in cellular c-di-AMP levels compared to the WT strain in the TSB medium (Fig. S4), when grown in the CDM without NH_3_, which enhances c-di-AMP production (30), the three *gdpP* mutant strains and five additional mutants (1S2E5, 1S3D6, 2S2F4, 2S2G10, and 2S3G9) showed a twofold increase in c-di-AMP levels in the culture supernatant compared to the WT (Fig. 3A). This result was verified using three biological replicates, and a more than twofold increase in c-di-AMP levels in the culture supernatant was seen for all five mutants compared to the WT TM283 strain (Fig. 3B). Among them, Tn insertions were found in the 5′ untranslated region (UTR) of the *nrdIEF* operon (strains 1S3D6 and 2S2F4 Tn::*nrdIEF*), within the *ybbR* gene (strain 2S2G10 *ybbR*::Tn), in an intergenic region within the operon coding for the known c-di-AMP-binding partner PstA (strain 2S3G9 *pstA*::Tn::*holB*), and in the 5′ UTR region upstream of *cshA* coding for an ATP-dependent RNA helicase (SAUSA300_2037) (Fig. S5; Table 1).

**Fig 4:**
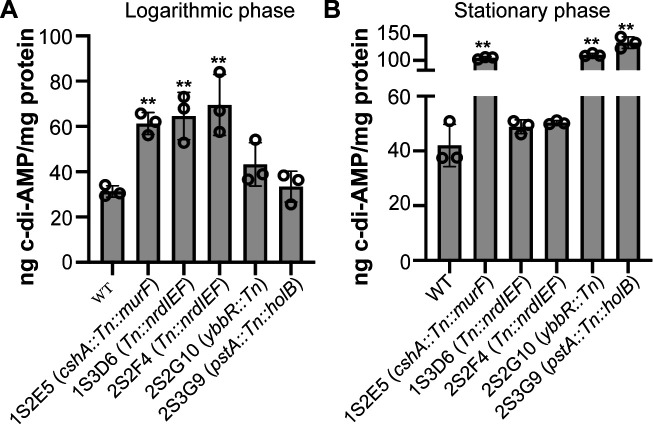
Increased c-di-AMP amounts are detected in cell extracts of several *S. aureus* transposon mutant strains. (**A and B**) Cell extracts were prepared from (**A**) log phase cultures and (**B**) stationary phase cultures of WT TM283 and five *S*. *aureus* transposon mutant strains harboring Tn insertions in genes or genomic regions as indicated on the *X*-axis label. c-di-AMP levels were determined using a competitive ELISA, and the average and standard deviation from three biological replicates were plotted. A one-way ANOVA followed by Dunnett’s *post hoc* test was used to identify statistically significant differences between WT and mutant strains. ** indicates *P*-value <0.01.

Since a clear increase in c-di-AMP levels in the supernatant fraction was observed for these five strains following growth in the CDM, additional ELISAs were performed to re-evaluate the cellular c-di-AMP levels using cell extracts prepared from logarithmic as well as stationary phase cultures following growth in CDM. All five strains showed statistically significant and a greater than twofold increase in cellular c-di-AMP levels, either in the log phase (strain *Tn::nrdIEF*) (Fig. 4A), the stationary phase (strains *ybbR*::*Tn* and *Tn::cshA*) (Fig. 4B), or both growth phases (strain *pstA::Tn::holB*) (Fig. 4). Taken together, besides *gdpP* mutants, five additional transposon mutants identified based on their lower fluorescence signal in the c-di-AMP biosensor screen were confirmed to produce higher c-di-AMP amounts. These strains contained insertions in *ybbR,* the *pstA* operon, the 5′ UTR regions of *cshA,* and the *nrdIEF* operon (two strains) (Fig. S5).

### Characterization of *S. aureus* mutant strains with a Tn insertion upstream of the *nrdIEF* operon

**Fig 5:**
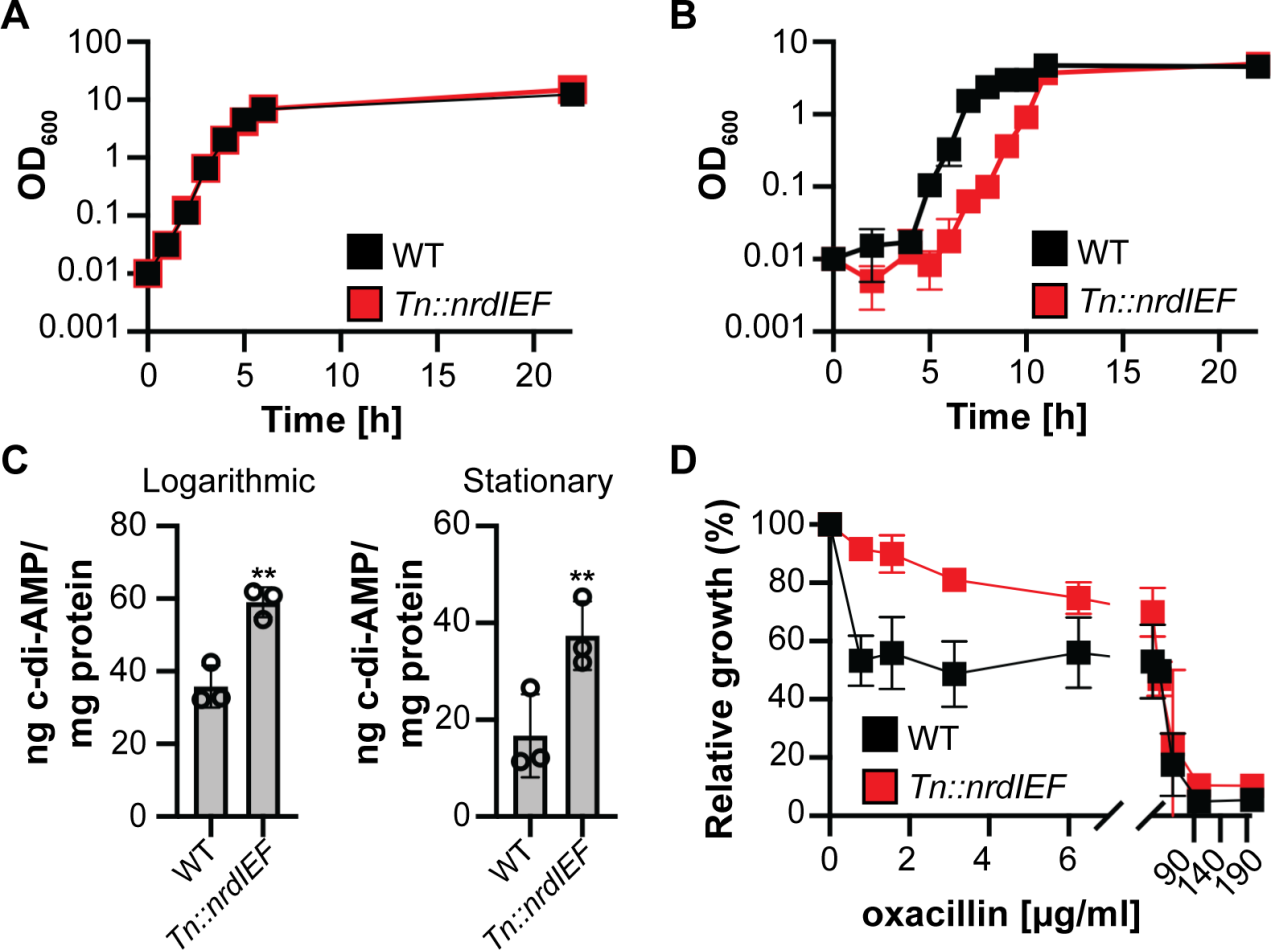
Growth and oxacillin resistance comparison between WT LAC* and the LAC* *Tn::nrdIEF* mutant strain. (**A and B**) Bacterial growth curves. WT LAC* and the isogenic LAC* *Tn::nrdIEF* mutant were grown in (**A**) TSB medium or (**B**) CDM in culture flasks, and OD_600_ values were determined at the indicated time points. The average values and standard deviations from three independent cultures were plotted. (**C**) Competitive ELISA to determine cellular c-di-AMP levels. WT LAC* and the isogenic LAC* *Tn:: nrdIEF* mutant strain were grown in CDM, cell extracts were prepared from logarithmic phase (left panel) and stationary phase (right panel) cultures, and c-di-AMP levels were determined using a competitive ELISA. The average values and standard deviations from three biological replicates were plotted, and a *t*-test was used to determine statistically significant differences between WT and the mutant. ** indicates *P*-value <0.01. (**D**) Oxacillin resistance assay. WT LAC* and the isogenic LAC* *Tn:: nrdIEF* mutant were grown in the presence of increasing concentrations of oxacillin and OD_600_ readings taken after 22 h of growth. The growth in the absence of oxacillin was set to 100% for each strain, % relative growth in the presence of oxacillin was calculated, and the average values and standard deviations of three cultures were plotted.

After *gdpP* mutants, strains containing Tn insertion upstream of the *nrdIEF* operon (*Tn::nrdIEF* strains 1S3D6 and 2S2F4) showed the lowest fluorescence values using the biosensor assay as well as highest c-di-AMP levels in the supernatant fraction and cell extracts prepared from log phase cultures. NrdEF codes for a class Ib RNR, which converts nucleotides to deoxynucleotides in bacterial cells. RNRs have not been previously linked to changes in c-di-AMP levels in *S. aureus*, and hence, we decided to further characterize these mutants. To ensure that the observed change in c-di-AMP levels in the *Tn::nrdIEF* Tn mutant is universal in different and clinically relevant *S. aureus* strains, the Tn insertion from strain 1S3D6 was transduced into the MRSA strain LAC*, resulting in the generation of strain LAC* *Tn::nrdIEF*. Strain LAC* *Tn::nrdIEF* grew identical to the WT LAC* in rich medium (TSB); however, the strain exhibited a delay in growth in CDM (Fig. 5A and B). More importantly, and despite the delay in growth, the c-di-AMP amount was significantly higher in cell extracts prepared from strain LAC* *Tn::nrdIEF* compared to WT LAC*, in both log and stationary phase cultures (Fig. 5C).

In the previous work, it has been shown that *gdpP* mutants, which have increased c-di-AMP levels, have increased resistance toward beta-lactam antibiotics (46, 47). While no change in MIC toward the beta-lactam antibiotic oxacillin was observed for the LAC* *Tn::nrdIEF* mutation compared to the WT LAC* strain, a significantly higher growth rate was observed for strain LAC* *Tn::nrdIEF* compared to the LAC* in the presence of sub-MIC concentrations of oxacillin (Fig. 5D).

**Fig 6:**
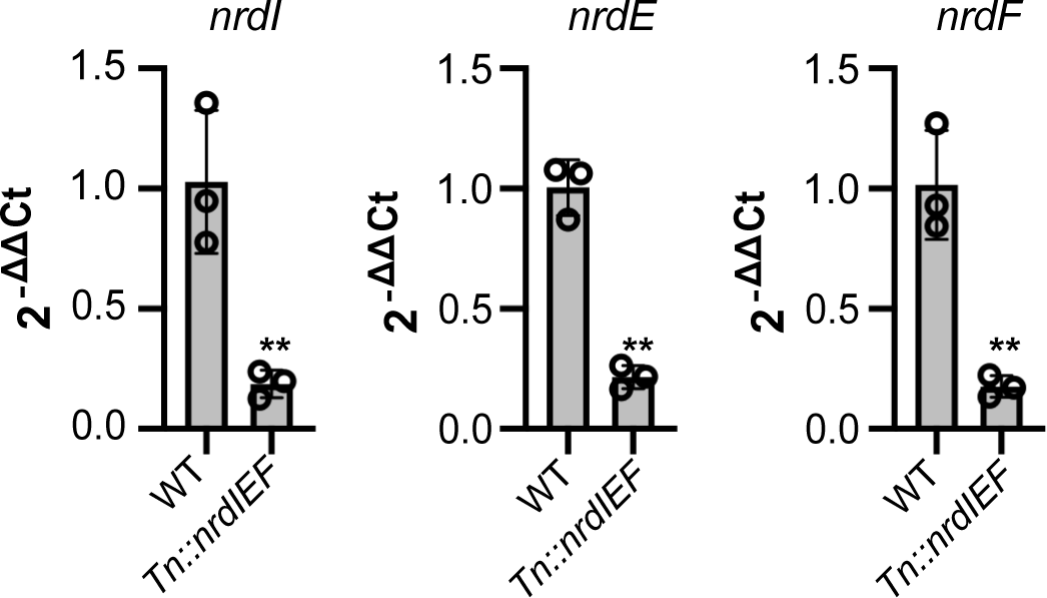
The transposon insertion in LAC* *Tn::nrdIEF* leads to reduced expression of the *nrdIEF* operon. qPCR analysis. RNA was extracted from logarithmic phase cultures of WT LAC* and the isogenic LAC* *Tn::nrdIEF* mutant strain following growth in TSB medium. The expression levels of the *nrdIEF* transcript were compared between the WT and mutant strain using *nrdI, nrdE,* and *nrdF* probes and normalized against the *gyrB* housekeeping gene. The average and standard deviation from three samples are plotted, and a *t*-test was used to determine statistically significant differences in *nrdI, nrdE,* and *nrdF* transcript levels between WT and the mutant strain. ** indicates *P*-value <0.01.

To investigate how the Tn insertion upstream of the *nrdIEF* operon affects downstream gene expression, the *nrdIEF* expression levels were determined by quantitative polymerase chain reaction (qPCR) for the WT LAC* strain and the LAC* *Tn::nrdIEF* mutant. A significant reduction in mRNA levels was observed in the mutant when using probes targeting *nrdI, nrdE,* or *nrdF* (Fig. 6), highlighting that the Tn insertion disrupts *nrdIEF* expression. As RNRs produce deoxyribonucleotides, we examined whether the addition of exogenous deoxyribonucleotides might complement the effect of *nrdIEF* down-regulation. The addition of deoxynucleotide triphosphates (dNTPs) to CDM slightly promoted the growth of the WT strain, but more importantly, it significantly improved the growth of the *Tn::nrdIEF* mutant to near WT levels (Fig. S6A). Furthermore, the addition of deoxyribonucleotide attenuated the effect of the *Tn::nrdIEF* mutation on c-di-AMP production compared to the WT, and the c-di-AMP levels in WT and the *Tn::nrdIEF* mutant strain were now almost identical and no longer significantly increased in the mutant (Fig. S6B). However, we also noticed that upon the supplementation of CDM with dNTPs, a general increase in c-di-AMP levels was observed in the WT strain (Fig. S6B). In *Escherichia coli,* dNTPs have been shown to serve as a nitrogen source under nitrogen-limiting conditions (48), and different nitrogen sources such as ammonium and purines have been shown to significantly impact c-di-AMP production in *S. aureus* (30, 32)*,* which could all contribute to the observed increase in c-di-AMP production in the WT strain upon the addition of dNTPs. Combining these results with the other findings, we concluded that a reduction in the production of the RNR enzyme leads to an increase in c-di-AMP production.

### The impact of decreased *nrdIEF* expression on c-di-AMP synthase, hydrolase, and target proteins

**Fig 7:**
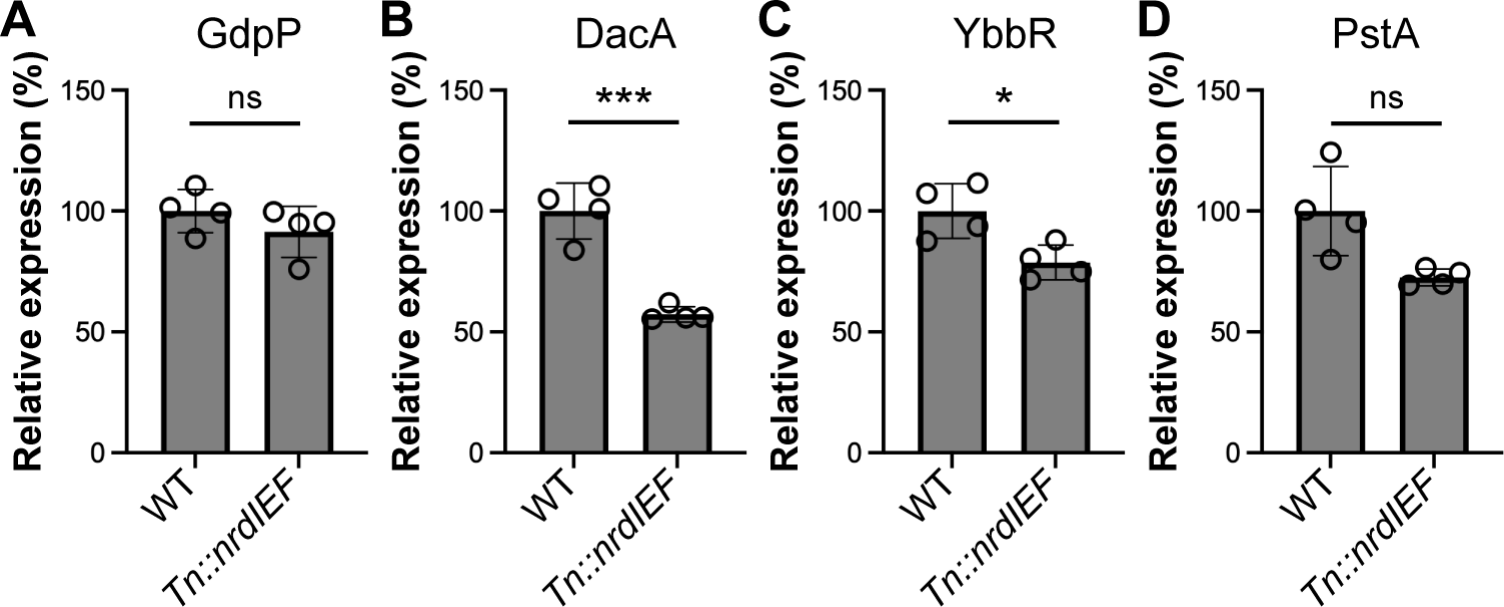
Comparison of GdpP, DacA, YbbR, and PstA protein levels between WT and Tn::*nrdIEF* mutant strains. Relative GdpP (**A**), DacA (**B**), YbbR (**C**), and PstA (**D**) protein levels in *S. aureu*s WT and the Tn::*nrdIEF* mutant as determined by western blot. Samples for the western blot analysis were prepared from cultures normalized to an OD_600_ of 3, and the protein band intensity of each sample was further normalized based on the band intensity of the L6 ribosomal protein as described in Materials and Methods. The average and standard deviation from four samples were plotted, and the average protein band intensity for the WT strain was set to 1. For statistical analysis, a *t*-test was used to determine statistically significant differences in protein level between the WT and *Tn::nrdIEF* mutant strains. For the PstA protein shown in panel (D), a Welch correction was performed due to the significant difference in variance between populations. *** indicates *P*-value <0.001, * indicates *P*-value <0.05, and ns denotes not statistically significant.

We then aim to investigate the mechanism of c-di-AMP production regulation by RNR. The conversion of ribonucleotides to deoxyribonucleotides by RNR enzymes impacts many cellular and metabolic processes in bacteria. Notably, in *B. subtilis* under starvation conditions*,* NrdEF overproduction results in a decrease in the production of several c-di-AMP-associated proteins, including the c-di-AMP cyclase DisA, the DacA regulator CdaR (YbbR in *S. aureus*), and the c-di-AMP target protein DarA (named PstA in *S. aureus*) (49). Hence, the effect of *nrdIEF* expression on the c-di-AMP level could be explained by changes in c-di-AMP regulator proteins such as GdpP, DacA, or YbbR. To test this hypothesis, we compared the levels of the GdpP, DacA, YbbR, and PstA proteins between WT LAC* and the Tn::*nrdIEF* mutant by western blot. This analysis revealed that GdpP and PstA levels were not significantly changed in the Tn::*nrdIEF* mutant (Fig. 7; Fig. S7). On the other hand, DacA and YbbR protein levels were decreased in the Tn::*nrdIEF* mutant (Fig. 7; Fig. S7). Since the c-di-AMP levels are increased in the Tn::*nrdIEF* mutant, a decrease in the DacA cyclase level and the unaltered GdpP phosphodiesterase protein levels cannot explain the elevated c-di-AMP levels in the mutant. Therefore, is it more likely that alteration in enzyme activity rather than in protein levels contributes to the increase in the cellular c-di-AMP levels in the Tn::*nrdIEF* mutant.

### The effect of reduced *nrdIEF* expression on c-di-AMP levels is dependent on the (p)ppGpp synthase RSH

**Fig 8:**
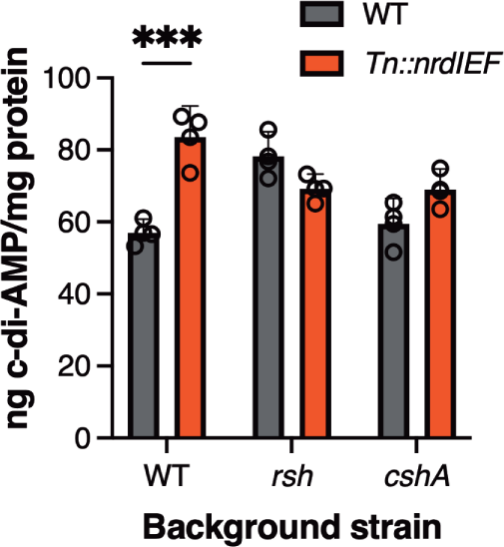
The increase in c-di-AMP in the *Tn::nrdIEF* mutant strain is dependent on the (p)ppGpp synthase RSH. ELISA assay to determine c-di-AMP levels in WT, *Tn::nrdIEF*, *rsh,* and *cshA::Tn-Kan* single mutants and in the *rsh/Tn::nrdIEF* and *cshA::Tn-Kan/Tn::nrdIEF* double mutant strains. c-di-AMP levels were determined using a competitive ELISA, and the average and standard deviation from three biological replicates were plotted. A two-way ANOVA with Sidak’s *post hoc* test was used to identify the statistically significant impact of *Tn::nrdIEF* mutation in WT, *rsh,* and *cshA*::Tn background strains. *** indicates *P*-value <0.001.

One of the factors that regulate the activity of c-di-AMP-metabolizing enzyme is the stringent response molecule (p)ppGpp, which inhibits GdpP activity at least *in vitro* (29). (p)ppGpp is also a negative regulator of *cshA* gene expression (50–52), which is interesting since one of the transposon insertion mutants with an increase in c-di-AMP levels had a Tn insertion just upstream of *cshA*. Interestingly, when we assessed *cshA* transcript levels in the *nrdIEF* mutant, we found a twofold increase compared to WT (Fig. S8). To more specifically investigate if the observed increase in c-di-AMP production in the *Tn::nrdIEF* mutant is dependent on the activity of RSH, the main (p)ppGpp synthase, or the activity of the CshA helicase, we constructed *rsh/Tn::nrdIEF* and *cshA/Tn::nrdIEF* double mutant stains and assessed c-di-AMP levels in the WT, *rsh,* and *cshA* single and respective double mutant strains (Fig. 8). These data revealed that while the c-di-AMP levels still remained slightly but no longer significantly elevated in the *cshA/Tn::nrdIEF* double mutant strain, the increase in c-di-AMP levels was completely abolished in the *rsh/Tn::nrdIEF* double mutant strain, and indeed, a slight reduction in the c-di-AMP levels was observed (Fig. 8). These data suggest that changes in CshA activity or expression might have a small contribution to the observed changes in c-di-AMP levels in the *Tn::nrdIEF* mutant strains. On the other hand, the increase in c-di-AMP levels in a strain with reduced *nrdIEF* expression is absolutely dependent on the activity of the main (p)ppGpp synthase RSH.

## DISCUSSION

Here, we show that a *B. subtilis kimA* riboswitch-based c-di-AMP biosensor can be used to detect differences in cellular c-di-AMP levels in *S. aureus*. When combined with a large mutant library, this is an effective way of identifying novel genetic factors that regulate c-di-AMP levels. As a proof of concept, we identified several genes coding for proteins with an established connection to the c-di-AMP signaling network, including strains with Tn insertions in *gdpP, ybbR,* and the operon coding for the c-di-AMP-binding protein PstA. More importantly, three additional mutants that were previously not known to be connected to the c-di-AMP signaling network in *S. aureus* were confirmed to exhibit elevated c-di-AMP levels. One mutant contained an insertion upstream of *cshA,* coding for a DEAD-box RNA helicase. Interestingly, *cshA* is located immediately downstream of the *kdp* genes coding for the KdpDE two-component system and the inducible KdpABC potassium transporter system. In *S. aureus,* c-di-AMP has been shown to bind to the sensor histidine kinase KdpD (16, 19), and this binding is thought to inhibit the expression of the *kdpABC* genes. Two other mutants with elevated c-di-AMP levels contained transposon insertions upstream of *nrdIEF*. This operon has not been associated with c-di-AMP in *S. aureus* before, although in other bacteria, indirect links have been established. For example, the repressor of *nrdIEF* in *L. monocytogenes,* NrdR, binds to c-di-AMP, and NrdEF overexpression in *B. subtilis* under starvation conditions reduces DisA and increases CdaR and DarA protein levels (49, 53). We show here that the Tn insertion leads to reduced expression of the *nrdIEF* genes, which in turn implicates that the RNR enzyme NrdEF is a negative regulator of c-di-AMP production in *S. aureus*. As the level of the c-di-AMP hydrolase GdpP was unaltered and the levels of the c-di-AMP synthase DacA were decreased in the *Tn::nrdIEF* mutant, the increase in c-di-AMP in this mutant strain cannot be explained by the alteration in GdpP or DacA production and, hence, is likely due to a change in enzyme activity. One factor that is known to affect the activity of the c-di-AMP-metabolizing enzymes is the nucleotide alarmone (p)ppGpp, which has been shown to inhibit the c-di-AMP-hydrolase GdpP (29). While we found that the activity of the main (p)ppGpp synthase Rel plays a key role in the observed increase in c-di-AMP production in the *Tn::nrdIEF* mutant, to assess if this is due to the direct binding of (p)ppGpp to GdpP and regulating its activity or through an indirect effect remains to be determined. However, we demonstrated that deoxyribonucleotides, products of ribonucleotide reductase activity, promoted c-di-AMP production in WT but not in the Tn::*nrdIEF* mutant and abolished the effect of the decrease in *nrdIEF* transcript on c-di-AMP levels, suggesting an additional link between RNR activity, nucleotide metabolism, and c-di-AMP signaling.

To conclude, in this study, an efficient high-throughput method for screening for factors that regulate c-di-AMP production in *S. aureus* was developed. While we used a 96-well plate-based method and screened individual mutant strains as part of this study, the number of mutants analyzed could be significantly increased by performing a FACS-based screen in which strains with fluorescence above or below the specific threshold are sorted (54, 55). In this manner, it would be possible to screen mutants in most of the non-essential genes. We noticed a certain degree of inconsistency between the fluorescence observed in the biosensor assay, and actual cellular c-di-AMP levels as assessed by ELISA and several mutants that exhibit reproducibly altered fluorescence did not show altered c-di-AMP production. This may be a result of Tn mutation-derived alterations in plasmid copy number (56), independent of c-di-AMP levels. To address this limitation, in future studies, an additional constitutively expressed fluorophore could be added to the biosensor plasmid to normalize for plasmid copy number (57), or the biosensor could be integrated into the chromosome to eliminate the influence of copy number variation. c-di-AMP is produced by many pathogenic and non-pathogenic bacteria (21). The methods developed in this study can be readily adapted for use in other bacterial species to better understand the role of c-di-AMP and potentially provide deeper insights into the relationship between c-di-AMP regulation and antibiotic resistance.

## MATERIALS AND METHODS

### Bacterial strains and growth conditions

The strains and plasmids used in the study are listed in Table S1. *S. aureus* strains were grown either in TSB, a rich medium, CDM prepared as previously described (15), CDM without NH_3_, or Mueller Hinton Broth (MHB) supplemented with 2% (wt/vol) NaCl. The *S. aureus dacA* mutant was grown in TSB supplemented with 0.6 M KCl. All *E. coli* strains were grown in lysogenic broth. Where appropriate, antibiotics were used at the following concentration: 100 µg/mL ampicillin, 10 µg/mL chloramphenicol, and 10 µg/mL erythromycin. The LAC* Tn::*nrdIEF* strain (ANG6122) was constructed by transducing the Tn::*nrdIEF* region from strain TM283 Tn::*nrdIEF* (strain 1S3D6–ANG6120) into the *S. aureus* LAC* strain using phage 85. The LAC* *rsh* Tn::*nrdIEF* double mutant (ANG6537) was constructed by transducing the Tn::*nrdIEF* region from the LAC* Tn::*nrdIEF* strain (ANG6122) into the *rsh* mutant strain lacking three amino acids in the *rsh* gene and hence unable to synthesize (p)ppGpp via the main (p)ppGpp synthase Rel (29, 58). For the construction of the *cshA/Tn::nrdIEF* double mutant strain, the erythromycin resistance marker in the Nebraska Transposon Mutant Library strain JE2 *cshA::Tn* (ErmR) (NE565/ANG6642) (59) was first replaced by allelic exchange with a kanamycin resistance cassette using plasmid pKAN (60). This resulted in the construction of strain JE *cshA::Tn-Kan* (ANG6643). Strains LAC* *cshA::Tn-Kan* (ANG6652) and LAC* *cshA::Tn-Kan Tn::nrdIEF* (ANG6653) were obtained by transducing the *cshA::Tn-Kan* mutation with phage 11 from strain JE2 *cshA::Tn-Kan* into WT LAC* and strain LAC* *Tn::nrdIEF,* respectively.

### Biosensor plasmid construction

The primers used in this study for plasmid or strain construction are listed in Table S2. Plasmid pCN34e (strain ANG4060) was produced by replacing the *aphA-3* gene in plasmid pCN34 (strain ANG201) with the *ermC* gene from plasmid pCN49 (strain ANG203) (61). To this end, plasmid pCN34 was digested with AvrII and SacII and ligated with the *aphA-3* fragment excised from plasmid pCN49 with the same enzymes. The vectors pRSLG2 and pRSLF3 containing the *kimA*-yfp and *ktrA-yfp* fragments under the control of a modified constitutive spac promoter (lacking the *lacI* operator sites) were obtained from the laboratory of Prof. Wade C. Winkler, University of Maryland, USA. To produce the pCN34e-*kimA-yfp* plasmid (strain ANG4097) and pCN34e-*ktrA-yfp* plasmid (strain ANG4096), the promoter *kimA-yfp* and promoter *ktrA-yfp* fragments were excised from plasmids pRSLF3 and pRSLG2 with BamHI and EcoRI and the fragments ligated with BamHI and EcoRI cut pCN34e. For the production of the riboswitch-lacking control vector pCN34e-*yfp* (strain ANG4095), a large part of the *ktrA* riboswitch region was deleted from plasmid pRSLF3 and the remaining promoter-*yfp* fragment cloned into plasmid pCN34e. To this end, primer pair ANG2347/ANG2348 was used to amplify the promoter region, and only part of the *ktrA* riboswitch region from plasmid pRSLF3 and primer pair ANG2349/ANG2350 was used to amplify the *yfp* gene region also from plasmid pRSLF3. The two fragments were fused by PCR using primers ANG2347/ANG2350, cut with BamHI and EcoRI, and ligated with BamHI and EcoRI cut pCN34e plasmid. Plasmids pCN38-*ktrA-yfp* (strain ANG5369), pCN38-*kimA-yfp* (strain ANG5370), and pCN38-*yfp* (strain ANG5368) were prepared by replacing the erythromycin resistance marker in the pCN34e-derived plasmids with a chloramphenicol resistance marker. To this end, plasmids pCN34e-*yfp*, pCN34e-*kimA-yfp,* and pCN34e-*ktrA-yfp* were cut with AvrII and XhoI to remove the *ermC* gene, and the cut plasmids were ligated with the c*at194* gene fragment, which was excised from plasmid pCN38 with restriction enzymes AvrII and XhoI. All plasmids were initially recovered in *E. coli* strain XL1-Blue and subsequently introduced into *S. aureus* strain RN4220 and then into *S. aureus* strain TM283 and isogenic *dacA*_G206S_ and *gdpP* mutant strains or *S. aureus* strain LAC* and isogenic *dacA*_G206S_ and *gdpP::kan* mutant strains (see Table S1 with the bacterial strain list). The sequences for plasmids pCN34e, pCN34e-*yfp,* pCN34e-*kimA-yfp,* pCN34e-*ktrA-yfp,* pCN38, pCN38-*yfp,* pCN38-*kimA-yfp,* and pCN38-*ktrA-yfp* were determined, and the sequences of the respective inserts verified by nanopore sequencing at the Full Circle Laboratory at Imperial College London and the sequences are provided as supplemental text in the supplemental materials file.

### Biosensor assay using a plate reader

The fluorescence of *S. aureus* strains containing the biosensor or control plasmids was measured using a plate reader. For end point measurements, colonies of the indicated WT, *gdpP*, *dacA_G206S_,* or other mutant strains were picked and used to inoculate 100-µL TSB medium supplemented with 10-µg/mL chloramphenicol in 96-well plates. The plates were then incubated at 37°C for 18 h with shaking at 500 rpm. Following the incubation, the OD_600_ and fluorescence readings were determined using a Tecan Infinite 200Pro plate reader. Fluorescence measurements were performed using excitation and emission wavelengths of 488 and 535 nm, respectively. OD_600_ and fluorescence values for media-only wells were subtracted from the sample readings, and normalized fluorescence values were calculated by dividing the background-corrected fluorescence reading by the background-corrected OD_600_ readings for each culture. For kinetic assays, cultures of the indicated WT, *dacA_G206S_,* and *gdpP* mutants were grown overnight at 37°C with shaking in 5-mL TSB supplemented with 10-µg/mL chloramphenicol. The next day, the bacteria from the 1-mL culture were collected by centrifugation for 5 min at 16,200 × *g* and washed twice with 1-mL phosphate-buffered saline (PBS) pH 7.4, and the OD_600_ of the cultures was measured and adjusted to an OD_600_ of 0.01. One hundred microliters of the diluted cultures was transferred into wells of a black-walled 96-well plate with a clear bottom, and the plate was incubated at 37°C with shaking in a Tecan Infinite plate reader. Fluorescence and OD_600_ readings were taken every 15 min, and normalized fluorescence values for each time point were calculated as described above.

### FACS analysis

For FACS analysis, 5-mL cultures of the indicated WT, *dacA_G206S_,* and *gdpP* mutant strains harboring the biosensor or control plasmids were grown overnight at 37°C with shaking in TSB medium supplemented with 10-µg/mL chloramphenicol. The next day, bacteria from a culture aliquot equivalent to 1 mL of OD_600_ of 1 was collected by centrifugation for 3 min at 17,000 × *g* and washed three times with 1-mL suspension buffer (0.06 M Na_2_HPO_4_, 0.06 M NaH_2_PO_4_, 5 mM KCl, 130 mM NaCl, 1.3 mM CaCl_2_, 0.5 mM MgCl_2_, and 10 mM glucose set to pH 7.2). An aliquot of the washed cells was diluted in suspension buffer to an OD_600_ of 0.02 (giving around 10^7^ cells per mL) and analyzed using a FACS Calibur machine with an excitation of 488 nm and emission of 530 nm.

### Microscopy analysis

For microscopy analysis, overnight cultures of the *S. aureus* LAC* strain and isogenic *gdpP::kan* and *dacA*_G206S_ mutant strains harboring plasmids pCN34e, pCN34e-*yfp*, pCN34e-*kimA-yfp,* or pCN34e-*ktrA-yfp* were grown overnight at 37°C with shaking in 5 mL of TSB medium supplemented with 10-µg/mL erythromycin. The next day, 1.5 µL of the overnight cultures was spotted onto flat 1.5% agarose pads, and phase contrast and fluorescence images (using a *yfp* filter set and excitation and emission wavelength of 488 and 530 nm) were taken using a Zeiss Axio Imager.A2 microscope. Three biological replicates were imaged, and representative results are shown.

### Screening of a *S. aureus* Tn mutant library for clones with altered fluorescence

To identify *S. aureus* mutants with altered c-di-AMP levels, the pCN38-*kimA-yfp* biosensor plasmid was introduced by electroporation into a previously described *S. aureus* Tn mutant library (8). To this end, an aliquot of the transposon library was adjusted to an OD_600_ of 0.1 in 200 mL of TSB supplemented with 10-µg/mL erythromycin. The culture was incubated at 37°C with shaking at 180 rpm until reaching an OD_600_ of 3, at which point electro-competent cells were prepared, and the pCN38-*kimA-yfp* biosensor plasmid was introduced by electroporation. Transformants were recovered on tryptic soy agar (TSA) plates supplemented with 10-µg/mL erythromycin and 10-µg/mL chloramphenicol. Two independent transformations were performed, and subsequently, a total of 431 colonies were screened for altered normalized fluorescence signals compared to the WT TM283 control strain. To this end, individual transformants and colonies of the TM283 WT, *dacA*_G206S_, and *gdpP* control strains containing the pCN38-*ktrA-yfp* biosensor plasmid (six replicates for the control strains) were picked and used to inoculate wells of black-walled 96-well plates containing 100-µL TSB medium supplemented with 10-µg/mL chloramphenicol. The 96-well plates were incubated at 37°C for 18 h in a plate shaker, shaking at 500 rpm. Next, fluorescence and OD_600_ readings were taken using a Tecan Infinite plate reader, and normalized fluorescence values were calculated as described above. A relative normalized fluorescence value for each of the Tn mutant strains was calculated as the percentage of normalized fluorescence relative to the value obtained for the WT TM283 pCN38-*ktrA-yfp* strain, which was set to 100%. Cultures exhibiting a twofold increase (suggesting lower c-di-AMP levels) or twofold decrease (suggesting higher c-di-AMP levels) were re-streaked for single colonies and re-tested using triplicate samples. Strains, which gave relative normalized fluorescence values lower than 75% compared to WT TM283 pCN38-*ktrA-yfp,* were further analyzed.

### Determination of c-di-AMP levels using a competitive ELISA

A competitive ELISA was used to determine the c-di-AMP levels for Tn mutant strains that gave relative normalized fluorescence values of below 75% compared to the WT TM283 pCN38-*ktrA-yfp* strain. For the initial analysis, the assay was performed using only one biological replicate for each strain and using cell extracts (intracellular c-di-AMP levels) as well as supernatant samples (extracellular c-di-AMP levels). Strains that exhibited a greater than twofold change in c-di-AMP levels were subsequently analyzed in triplicate, and extracellular c-di-AMP levels were determined from stationary phase cultures and intracellular c-di-AMP levels from logarithmic phase and stationary phase cultures. To measure the intracellular c-di-AMP level from stationary phase cultures, single colonies of the indicated bacterial strains were inoculated into 5-mL TSB or CDM, and the cultures were incubated for 18 h at 37°C with shaking. Next, bacteria from 1.5 mL of the TSB-grown culture or 3 mL of the CDM-grown culture were collected by centrifugation for 5 min at 16,200 × *g*, washed twice with 1-mL PBS pH 7.4 buffer, and subsequently suspended in 750 µL of 50 mM Tris pH 8 buffer. For cell lysis, lysostaphin was added to a final concentration of 20 µg/mL, and the suspension was incubated for 30 min at 37°C. This was followed by a further mechanical lysis step, where the suspension was mixed with the equivalent of a 500-µL volume of 0.1-mm glass beads, and the cells were disrupted three times for 30 sec at setting 6 in a Fastprep-24 beat beater (MP Biomedicals). The solution was then centrifuged for 5 min at 16,200 × *g*, and the cleared lysate was transferred to a new microfuge tube. Twenty-five microliters of the solution was removed, and the protein content was determined using a Pierce BCA Protein Assay Kit (Thermo Fisher). The remaining lysate was boiled for 5 min and centrifuged for 10 min at 16,200 × *g*. The clear lysate was transferred to a new microfuge tube and stored frozen until performing the ELISA. For measuring intracellular c-di-AMP levels from log phase cultures, 5-mL overnight cultures were prepared in CDM at 37°C with shaking. The next day, the cultures were diluted into 25-mL CDM to a starting OD_600_ of 0.01 and incubated with shaking at 37°C until reaching an OD_600_ of approximately 0.5. At this point, bacteria from the equivalent of 8 OD_600_ units were collected by centrifugation for 10 min at 16,200 × *g* and washed twice with 1-mL PBS pH 7.4, and subsequently, cell extracts were prepared, and protein content was determined as described above. To examine the effect of dNTPs on c-di-AMP production, the cell extracts were prepared as described above, from bacteria grown in CDM or CDM supplemented with 4 mM of dNTP mix.

For measuring extracellular c-di-AMP levels, single colonies of the indicated bacterial strains were used to inoculate 5-mL CDM or CDM lacking NH_3_ as indicated and incubated for 18 h at 37°C with shaking. Next, 4.5 mL of the cultures was centrifugation for 5 min at 16,200 × *g*. Three milligrams of the cleared supernatant fraction was transferred to a new tube and freeze-dried. The dried supernatant fraction pellet was re-suspended in either 300 µL (for the WT and the Tn mutant strains) or 3 mL (for the *gdpP* mutant) of 50-mM Tris pH 8 buffer and stored frozen for the subsequent ELISA analysis. To determine the protein content of the cultures used for the supernatant sample preparation, bacteria from 1.5 mL of the culture were collected by centrifugation and washed, cell lysates were prepared, and the protein content was determined as described above.

Competitive ELISAs to determine c-di-AMP levels in the cell extracts or supernatant fraction samples were performed as described previously (30, 33). Prior to adding the samples to the ELISA plates, the cell extract samples for determining intracellular c-di-AMP levels were diluted to a concentration of 500 µg protein/mL (WT and Tn mutant samples) or 200 µg protein/mL (*gdpP* mutant sample). The supernatant samples were used without further dilution and where applicable readings normalized based on protein content after the assay.

### Mapping transposon insertion sites by arbitrary PCR

The transposon insertion sites were mapped by arbitrary PCR (62). Briefly, the first PCR was set up using gDNA of the different Tn mutant strains as template DNA and the arbitrarily binding primer ARB1 and the transposon-specific primer MarTnF (Table S2). The PCR steps using Taq polymerase were as follows: 5 min at 94°C followed by 5 cycles of 30 sec at 94°C, 30 sec at 30°C, and 90 sec at 72°C, followed by 30 cycles of 30 sec at 94°C, 30 sec at 45°C, and 90 sec at 72C°, and a final extension step at 72°C for 5 min. An aliquot of the first PCR reaction was used as template DNA for the second round of PCR, using the transposon-specific primer ITR2b and the chromosome-targeting primer ARB2. The reaction steps were as follows: 30 cycles of 30 sec at 94°C, 30 sec at 45°C, and 90 sec at 72°C. The PCR products were sequenced using primer ITR2b, and the Tn insertion site was mapped using BLAST and the *S. aureus* USA300_FPR3757 genome as reference.

### Bacterial growth curves in culture flasks

*S. aureus* strain LAC* and LAC* Tn::*nrdIEF* were grown overnight in 5-mL TSB or CDM (15) at 37°C with shaking. The next day, bacteria from a 1-mL culture aliquot were pelleted and washed once with 1-mL PBS buffer pH 7.4. The washed cultures were used to inoculate 25-mL TSB or CDM or where indicated in CDM supplemented with 4-mM dNTPs in 250-mL flasks to an OD_600_ of 0.01. The cultures were incubated at 37°C with shaking, the samples were removed, and OD_600_ readings were taken at the indicated time points.

### Bacterial growth assay in the presence of oxacillin

*S. aureus* strain LAC* and LAC* Tn::*nrdIEF* were grown overnight in 5-mL MHB + 2% NaCl at 37°C with shaking. The next day, the cultures were set to an OD_600_ of 0.02, and 50 µL was transferred to the appropriate number of wells of a 96-well plate. The diluted cultures were mixed with 50 µL of MHB + 2% NaCl containing oxacillin to give final concentrations of 200, 100, 50, 25, 12.5, 6.25, 3.125, 1.56250, 0.7812, and 0 µg/mL. The plates were incubated for 22 h at 37°C with shaking. Following incubation, OD_600_ readings were taken in a plate reader, the blank value of media-only-containing wells was subtracted from the sample OD_600_ readings, and % growth of the cultures in the presence of antibiotic was calculated by dividing the OD_600_ reading in the presence of antibiotic by the OD_600_ reading without oxacillin and multiplying by 100. These values were plotted as a function of oxacillin concentration. The experiment was performed in triplicate, and average and standard deviations were plotted.

### qPCR

RNA was isolated from *S. aureus* strains LAC* and LAC* Tn::*nrdIEF*. To this end, overnight cultures were grown in 5 mL of TSB at 37°C with shaking. The next day, the cultures were diluted to an OD_600_ of 0.01 in 5-mL fresh TSB and incubated at 37°C with shaking until reaching an OD_600_ of approximately 1. At this point, the cultures were diluted again to an OD_600_ of 0.01 into 40-mL TSB and incubated for 2 h at 37°C with shaking at 180 rpm. 46.6-mL GTC buffer (5 M guanidine thiocyanate, 0.5% N-lauryl sarcosine, 0.1 M β-mercaptoethanol, 0.5% Tween-80, and 10 mM Tris pH 7.5) was added to a 20-mL culture aliquot, and bacteria were pelleted by centrifugation for 10 min at 8,300 × *g*. The pellet was suspended in 1-mL GTC buffer and transferred to a microfuge tube, the bacteria were collected again by centrifugation for 3 min at 17,000 × *g,* and the pellet was suspended in 1-mL RNApro reagent solution from the MPBio RNA isolation kit and incubated for 1 min on ice. Bacterial cells were lysed using a Fastprep-24 beat beater (MP Biomedicals), and the RNA was extracted using the MPBio RNA kit according to the manufacturer’s instructions. The RNA samples were suspended in 100 µL of RNA rescue solution and incubated at 60°C for 10 min. The RNA samples were afterward further purified using Qiagen RNeasy spin columns following the manufacturer’s instructions. Following the clean-up and DNase treatment step, a second DNase treatment step was performed using Ambion TURBO DNA-free DNase according to the manufacturer’s instruction. Finally, using 1,000 or 100 ng of RNA per 20 µL of reaction, cDNA was produced using the RT SuperMix (Invitrogen). Transcript levels were determined for *nrdI*, *nrdE*, *nrdF*, *cshA,* and *gyrB* by qPCR using primers and probes listed in Table S2. Ten-microliter reactions were set up and consisted of 5-µL TaqMan fast mix (Thermo Fisher), 3.5-µL H_2_O, 0.5-µL primer/probe mix, and 1-µL 50 or 5 ng cDNA. The reactions were performed in a One-Step Real-Time PCR machine (Thermo Fisher) using the following conditions: 95°C for 20 sec followed by 40 cycles of 95°C for 3 sec and 60°C for 30 sec. The fold change in the expression level of each gene was calculated as 2^−ΔΔCt^, and the average and standard deviations from three independently isolated RNA samples were plotted.

### Western blot analysis

Western blot analysis was performed to compare DacA, YbbR, GdpP, and DacA protein levels and to control the L6 ribosomal protein levels in WT LAC* and the LAC* Tn:*nrdIEF* mutant strain. To this end, LAC* and LAC* Tn:*nrdIEF* overnight cultures were diluted 1:100 into a 5-mL fresh TSB medium, and the cultures were incubated at 37°C with shaking for about 3 h until reaching an OD_600_ of approximately 3. Next, bacteria from the equivalent of 1 mL of an OD_600_ of 3 were collected by centrifugation, and the pellet was resuspended in 50 µL of 50 mM Tris pH 8 buffer supplemented with 0.2-mg/mL lysostaphin and 0.1-mg/mL DNaseI final concentrations. The samples were incubated for 30 min at 37°C to lyse the bacteria. For the detection of DacA, 100 µL of urea buffer (30 mM Tris pH 6.5, 6 M urea, 3% SDS, and 7.5 mM MgCl_2_) was added, and the samples were incubated for a further 30 min. Following the incubation, the samples were centrifuged for 5 min at 16,200 × *g*, a 100-µL sample was removed and added to 20 µL of 6× SDS sample buffer (375 mM Tris-HCl_2_, 9% SDS, 50% glycerol, 0.03 bromophenol blue, and 9% beta-mercaptoethanol), and 10 µL was analyzed on SDS-PAGE gels. For the detection of GdpP, YbbR, and PstA, 50 µL of SDS sample buffer (62.5 mM Tris-HCl_2_ pH 6.8, 2% SDS, 10% glycerol, 0.01% bromophenol blue, and 10% beta-mercapto ethanol) was added directly to the lysed cells. The samples were boiled for 15 min and centrifuged for 5 min at 16,200 × *g,* and 10 µL was analyzed on SDS-PAGE gels. For GdpP quantification, 10% SDS-PAGE gels were used; for DacA, YbbR, and L6 quantification, 12% SDS-PAGE gels; and for PstA quantification, 15% SDS-PAGE gels. Following electrophoresis, proteins were electro-transferred to PVDF membranes; the membranes were blocked for 1 h with 20-mL TBST buffer [20 mM Tris-HCl pH 7.6, 136 mM NaCl, 0.1% (vol/vol) Tween 20] containing 5% milk and 7.5 µg/mL human IgG. Primary antibody incubations were performed overnight at 4°C in the same buffer with polyclonal rabbit antibodies raised against the *S. aureus* DacA, GdpP, YbbR, and PstA (Covalab) or the L6 ribosomal control protein (University of Chicago). L6, YbbR, PstA, and GdpP antibodies were used at a 1:10,000 and the DacA antibody at a 1:20,000 dilution. A horseradish peroxidase (HRP)-conjugated anti-rabbit IgG antibody (Cell Signalling Technologies) was used at a 1:10,000 dilution as a secondary antibody, and blots were incubated for 1 h at room temperature. The membranes were then developed using the Clarity ECL western blot substrate (Bio-Rad) according to the manufacturer’s instructions. Quantification was performed using the ImageJ software. For this, a background value measured at an empty spot on the blot was subtracted from the target protein band intensity value for each sample band, and this value was divided by the L6 protein value. The average and standard deviation of four samples were plotted, and the average value for the WT LAC* sample was set to 1 for each target protein.

## SUPPLEMENTAL MATERIAL

Supplemental material msphere.00321-24-s0001.pdf Supplemental tables, figures, and text with plasmid sequences.

ASM does not own the copyrights to Supplemental Material that may be linked to, or accessed through, an article. The authors have granted ASM a non-exclusive, world-wide license to publish the Supplemental Material files. Please contact the corresponding author directly for reuse.
