# Supplementary material for: Identification of novel genetic factors that regulate c-di-AMP production in Staphylococcus aureus using a riboswitch-based biosensor
Source: mSphere. 2024 Sep 17;9(10):e00321-24. doi: 10.1128/msphere.00321-24 (PMC11520302; doi:10.1128/msphere.00321-24)
Supplement: Supplemental material — Supplemental tables, figures, and text with plasmid sequences. [file msphere.00321-24-s0001.pdf]

Supplemental Material

Identification of novel genetic factors that regulate c-di-AMP production in *Staphylococcus aureus* using a riboswitch-based biosensor

Igor Kviatkovski[1], Qiyun Zhong[1,2], Sanika Vaidya[1,3], Angelika Gründling[1*]

ORCID IDs: 0000-0002-1358-7360 (IK); 0000-0001-7053-9640 (QZ); 0000-0001-6877-617X (SV); 0000-0002-6235-8687 (AG)

[1]Section of Molecular Microbiology and Centre for Bacterial Resistance Biology, Imperial College London, London, UK

[2]Current address: Division of Cancer Biology, Chester Beatty Laboratories, Institute of Cancer Research, 237 Fulham Road, London SW3 6JB, UK.

[3]Current address: Antimicrobial Discovery Center, Department of Biology, Northeastern University, Boston, MA 02115, USA

*Correspondance to: a.grundling@imperial.ac.uk

Running title: Factors regulating c-di-AMP production in *Staphylococcus aureus*

Table S1: Bacterial strains used in this study.

Strain number	Strain	Reference
S. aureus		
1575	LAC*	(1)
1961	LAC**gdpP:: kan*; KanR	(2)
3005	RN4220 pKAN (NE3004)	(3)
3301	LAC* Δ*ybbR*	(4)
3112	LAC* Δ*pstA*	This study
3165	LAC* rsh_{syn}	(5)
3666	LAC* *dacA::kan*	(6)
3729	TM283	(7)
3773	TM283 Δ*gdpP*	This study
3779	TM283 $dacA_{G206S}$	This study
4460	LAC* pCN34e; ErmR	This study
4461	LAC* pCN34e-*yfp*; ErmR	This study
4462	LAC* pCN34e-*ktrA-yfp*; ErmR	This study
4463	LAC* pCN34e-*kimA-yfp*; ErmR	This study
4464	LAC* *gdpP*::*kan* pCN34e; ErmR	This study
4465	LAC* *gdpP*::*kan* pCN34e-*yfp*; ErmR KanR	This study
4466	LAC* *gdpP*::*kan* pCN34e-*ktrA-yfp*; ErmR KanR	This study
4467	LAC* *gdpP*::*kan* pCN34e-*kimA-yfp*; ErmR KanR	This study
4468	LAC* $dacA_{G206S}$ pCN34e; ErmR KanR	This study
4469	LAC* $dacA_{G206S}$ pCN34e-*yfp*; ErmR	This study
4470	LAC* $dacA_{G206S}$ pCN34e-*ktrA-yfp*; ErmR	This study
4471	LAC* $dacA_{G206S}$ pCN34e-*kimA-yfp*; ErmR	This study
5354	TM283 pCN38; CamR	This study
5355	TM283 pCN38-*yfp*; CamR	This study
5356	TM283 pCN38-*kimA-yfp*; CamR	This study
5357	TM283 pCN38-*ktrA-yfp*; CamR	This study
5358	TM283 $dacA_{G206S}$ pCN38 CamR	This study
5359	TM283 $dacA_{G206S}$ pCN38-*yfp*; CamR	This study
5360	TM283 $dacA_{G206S}$ pCN38-*kimA-yfp*; CamR	This study
5361	TM283 $dacA_{G206S}$ pCN38-*ktrA-yfp*; CamR	This study
5362	TM283 ΔgdpP pCN38; CamR	This study
5363	TM283 Δ*gdpP* pCN38-*yfp*; CamR	This study
5364	TM283 Δ*gdpP* pCN38-*ktrA-yfp*; CamR	This study
5365	TM283 Δ*gdpP* pCN38-*kimA-yfp*; CamR	This study
6122	LAC* Tn::*nrdIEF*; ErmR	This study
6642	JE2 *cshA::Tn*; ErmR (NE565)	(8)
6643	JE2 *cshA::Tn-Kan*; KanR	This study
6652	LAC* *cshA::Tn-Kan*; KanR	This study

6653	LAC* *cshA::Tn-Kan Tn::nrdIEF*; ErmR, KanR	This study
E. coli		
127	XL1-Blue	Stratagene
1676	XL1-Blue pCN38; AmpR	This study
4029	XL1-Blue pRSLF3 (*ktrA-yfp*); AmpR	Wade C. Winkler lab
4030	XL1-Blue pRSLG2 (*kimA-yfp*); AmpR	Wade C. Winkler lab
4060	XL1-Blue pCN34e; AmpR	This study
4095	XL1-Blue pCN34e-*yfp*; AmpR	This study
4096	XL1-Blue pCN34e-*ktrA-yfp;* AmpR	This study
4097	XL1-Blue pCN34e-*kimA-yfp*; AmpR	This study
5368	XL1-Blue pCN38-*yfp*; AmpR	This study
5369	XL1-Blue pCN38-*ktrA-yfp*; AmpR	This study
5370	XL1-Blue pCN38-*kimA-yfp;* AmpR	This study

Table S2. Primers and probes used in this study.

Number	Primer	Sequence	Reference
		**Standard PCR**	
14	ARB1	GGCCACGCGTCGACTAGTACNNNNNNNNNNGATAT	(9)
15	ARB2	GGCCACGCGTCGACTAGTAC	(9)
28	MarTnF	TTTATGGTACCATTTCATTTTCCTGCTTTTTC	(10)
2347	5-BamHI-Prom	ATGAGGATCCGTAGCCCTTGCCTACCTAGC	This study
2348	3-Prom-YFP	CCTCCTTAAGCTTTTGGTTCCCCCGTTCGTGTTTGATTATGC	This study
2359	5-YFP	ACGGGGGAACCAAAAGCTTAAGGAGGAAAGTCACATTATGAG	This study
2350	3-Term-YFP	GAAACACACAAATTAAAAACTGGTCTGATCGG	This study
3576	ITR2b	CATAGTTCCTATATAGTTATGC	This study
		**Taqman qPCR**	
Taq1	*nrdI* F	CAGAACCCGTTCAATCTTTTTTAGAAGT	This study
Taq2	*nrdI* R	GCTTTTGCGAAATTTAGTCCCCAAT	This study
Taq3	*nrdI* probe	CTAGCTGCCACACCTC	This study
Taq4	*nrdE* F	GGTTGCAAACCCAAATGTTGAGAAA	This study
Taq5	*nrdE* R	CCTGATTGTAATTGTGTTTGCGCAAT	This study
Taq6	*nrdE* probe	TTTCACGCGCATTCTT	This study
Taq7	*nrdF* F	TCTCAGGTTTCTATTATCCACTATATCTTGCT	This study
Taq8	*nrdF* R	CGGTAAATACACCATGAATAGATTCATCTAAAAGAAT	This study
Taq9	*nrdF* probe	ATGTCGTCATTTTCCC	This study
Taq10	*cshA F*	TGTTCATCAACCTGAATTAGCAATCGTA	This study
Taq11	*cshA R*	CAAAGCACTTGTTAATTCATCAACACGA	This study
Taq12	*cshA* probe	TCGGACGTACAAAACG	This study

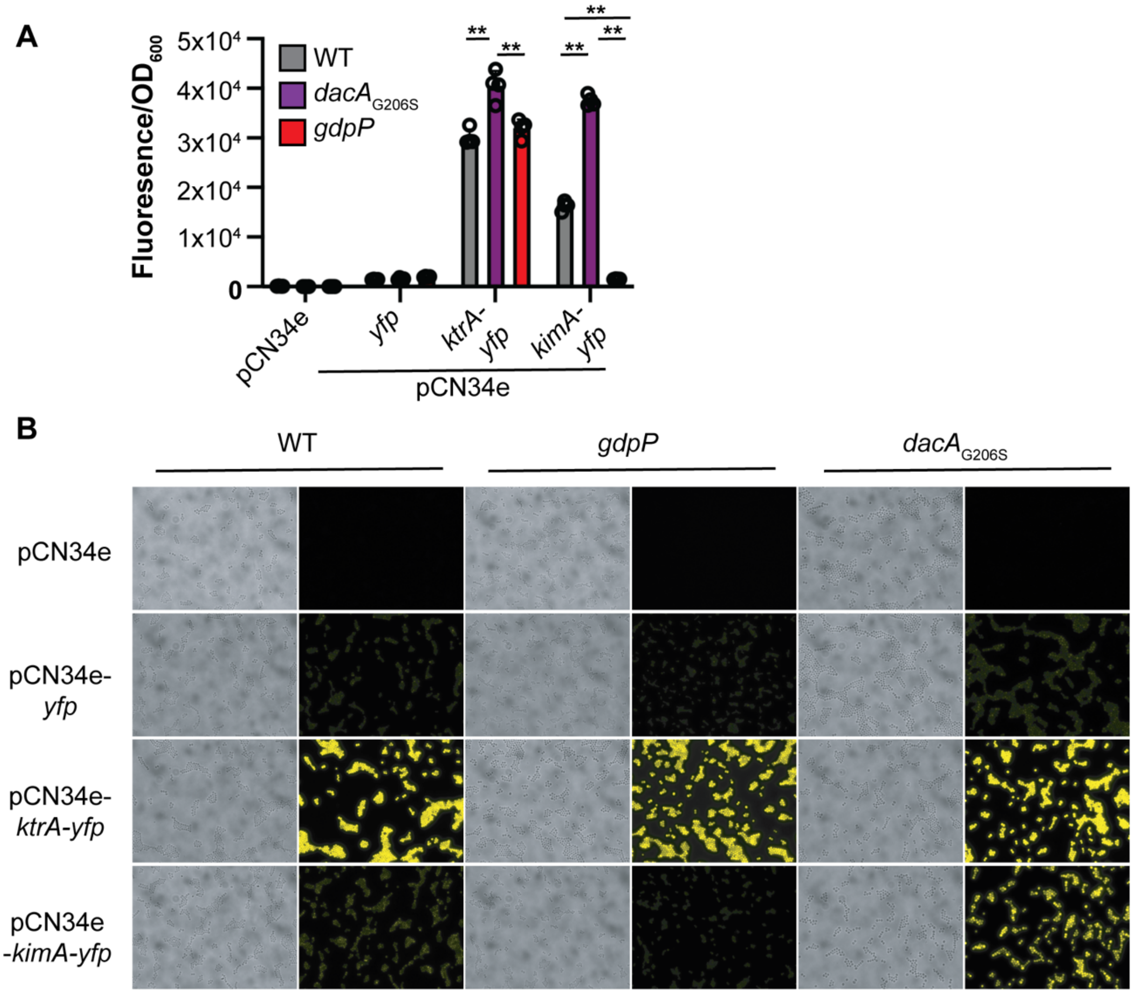

Figure S1. Characterization of the c-di-AMP biosensor plasmids pCN34e-*kimA-yfp* and pCN34e-*ktrA-yfp* in *S. aureus*. (A) End point fluorescence measurements. The WT *S. aureus* strain LAC* and isogenic *dacA*$_{G206S}$ and *gdpP* mutant strains containing the pCN34e or pCN34e-*yfp* control plasmids or the pCN34e-*ktrA-yfp* or pCN34e -*kimA-yfp* were grown in TSB medium in 96-well plates. After 18 h of growth, fluorescence and OD_{600} reading were taken and average values and standard deviations of the normalized fluorescence (Fluorescence/OD_{600}) from four independent cultures calculated and plotted. To determine statistically significant difference in the normalized fluorescence values between WT and mutant strains a two-way ANOVA followed by Tukey's post hoc test were performed. ** indicates p value < 0.01. (B) Microscopy analysis. Phase contrast and fluorescence images

were taken of WT LAC*, *dacA*$_{G206S}$ and *gdpP* mutant strains containing plasmids pCN34e, pCN34e-*yfp*, pCN34e-*kimA*-*yfp* or pCN34e-*ktrA*-*yfp*. Representative images of 3 independent experiments are shown.

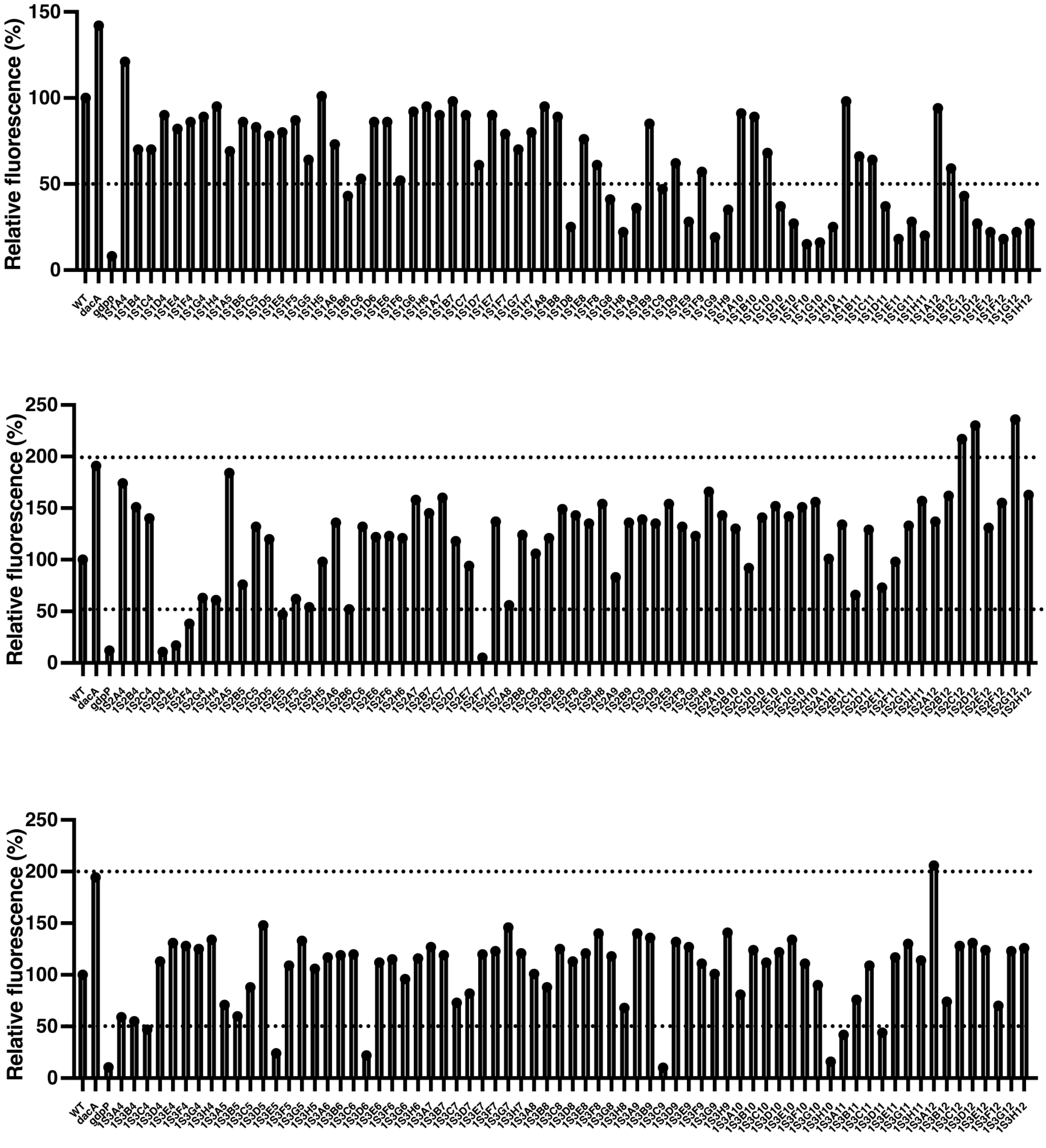

Figure S2. First biosensor screen. The biosensor plasmid pCN38-*kimA*-*yfp* was introduced into *S. aureus* TM283 transposon mutant library strains, transformants from this first transformation reaction picked and grown for 18 h in TSB medium in 96-well plates. WT TM283, $dacA_{G206S}$ and *gdpP* mutant strains containing plasmid pCN38-*kimA*-*yfp* were included on all plates as controls. Following growth, fluorescence and OD_{600} were determined, and

normalized fluorescence (Fluorescence/OD_{600}) values calculated, and relative fluorescence values compared to WT, which was set to 100%, plotted. Mutant strains, which showed relative fluorescence values of < 50% or > 200% compared to WT (dotted lines indicate threshold) were further analyzes and verification in replicates (see Fig. 2).

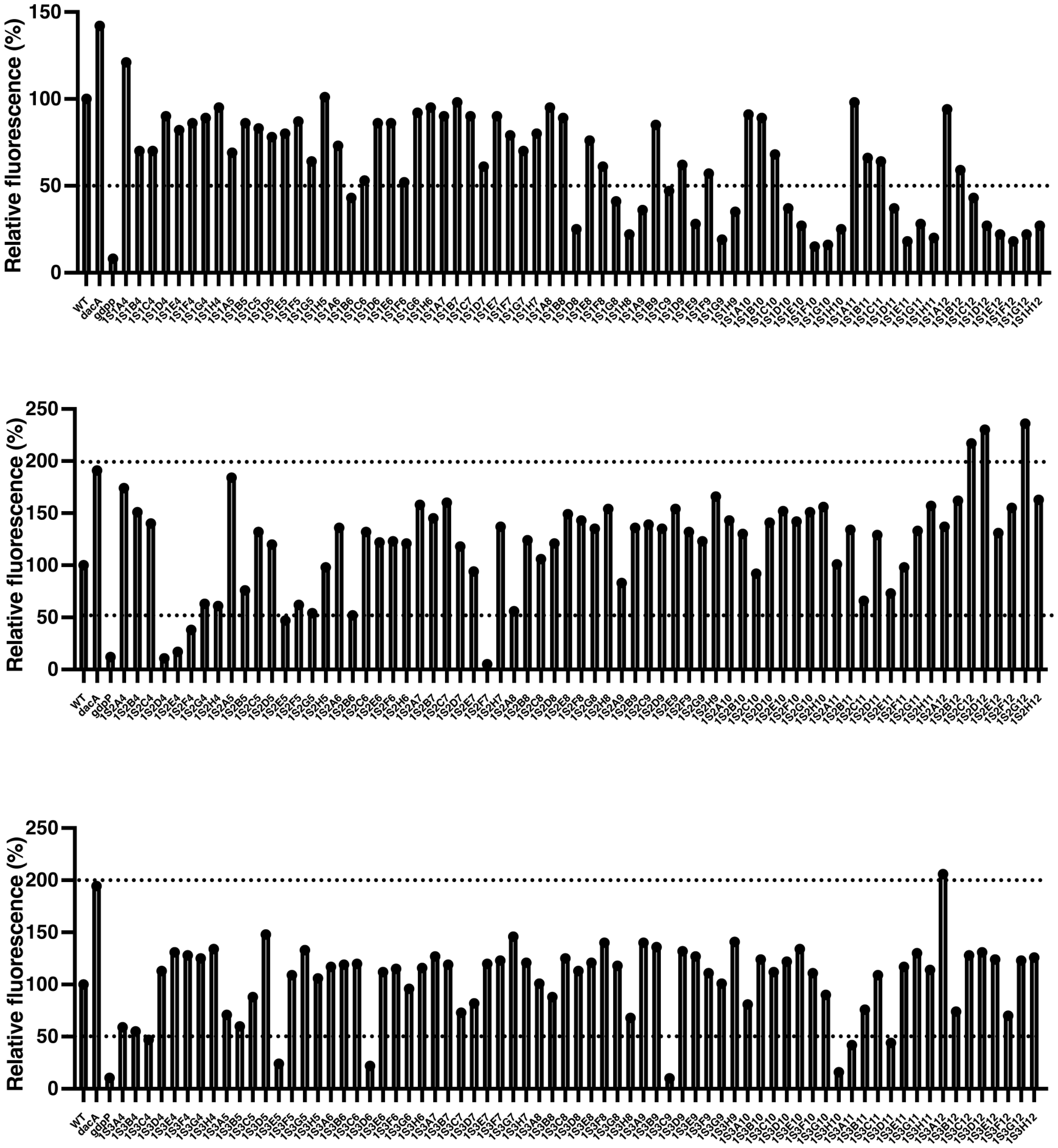

Figure S3. Second biosensor screen. The biosensor plasmid pCN38-*kimA*-*yfp* was introduced into *S. aureus* TM283 transposon mutant library strains, transformants from this second independent transformation reaction were picked and grown for 18 h in TSB medium in 96-well plates. WT TM283, *dacA*$_{G206S}$ and *gdpP* mutant strains containing plasmid pCN38-*kimA*-*yfp* were included on all plates as controls. Following growth, fluorescence and OD_{600} were

determined, and normalized fluorescence (Fluorescence/OD_{600}) values calculated, and relative fluorescence values compared to WT, which was set to 100%, plotted. Mutant strains, which showed relative fluorescence values of < 50% or > 200% compared to WT (dotted lines indicate threshold) were further analyzes and verification in replicates (see Fig. 2).

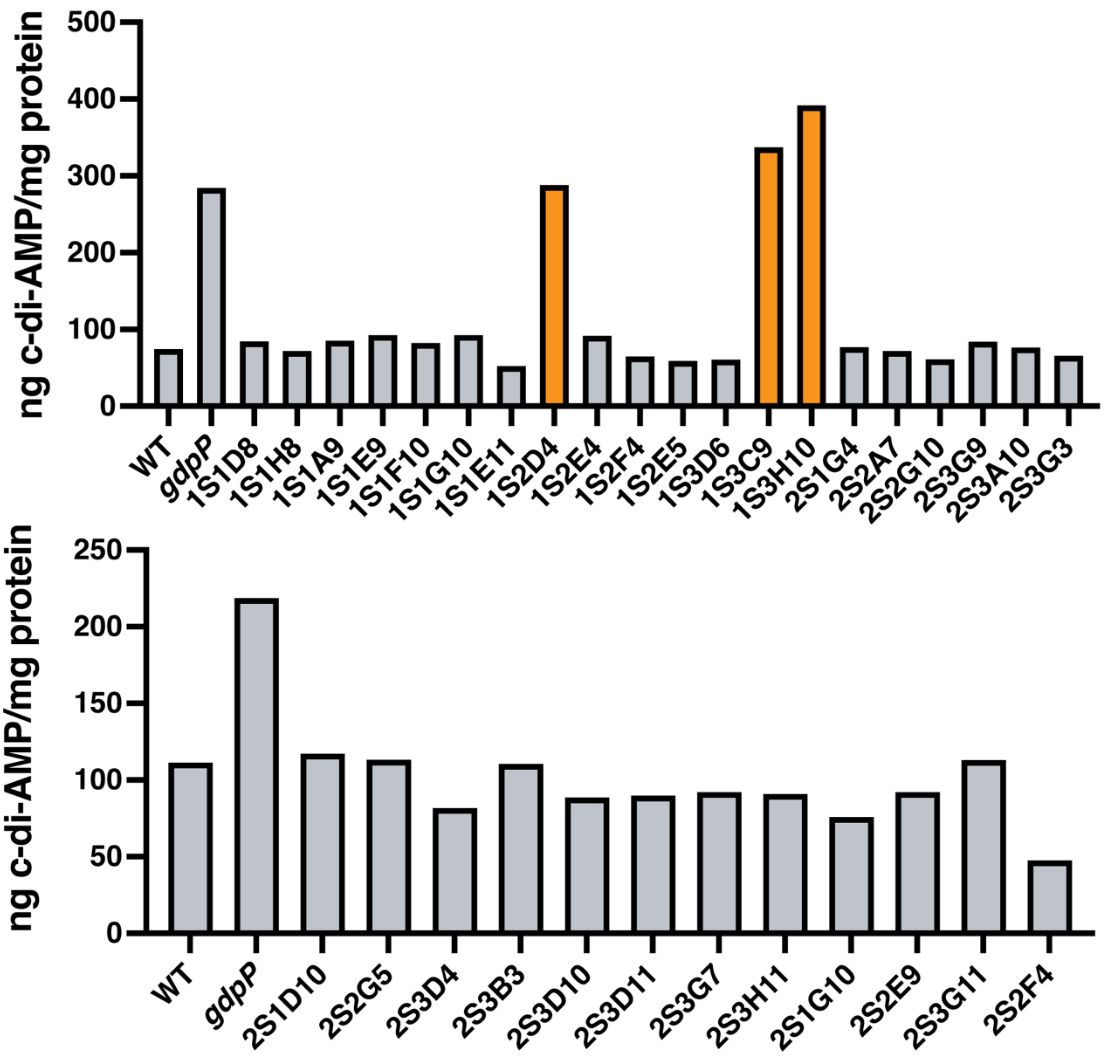

Figure S4. Cellular c-di-AMP in different *S. aureus* transposon mutant strains following growth in TSB medium. WT TM283, the *gdpP* mutant control strain and the 32 *S. aureus* transposon mutants (see Table S1), which showed reduced fluorescence compared to the WT strain in the biosensor assay, were grown overnight in TSB medium. Next day, cell extracts were prepared, and c-di-AMP levels determined using a competitive ELISA. Only strains containing transposon insertions in *gdpP* (highlighted in orange) showed a > 2 increase in cellular c-di-AMP levels. This initial c-di-AMP ELISA was performed using only one biological replicate, but multiple replicates were used in follow-up assays (see result section).

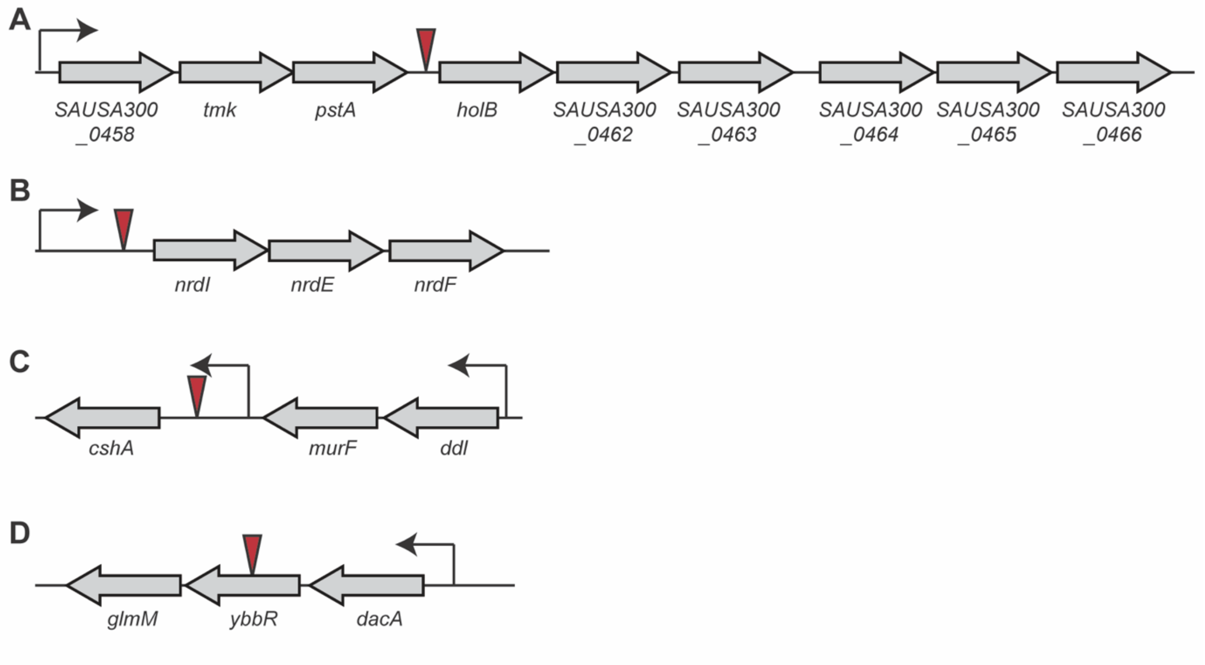

Figure S5. Schematic representation of the transposon insertions site in strains with confirmed increased c-di-AMP production. (A) Strain 2S3G9 with a transposon insertion in the *pstA* operon between *pstA* and *holB*, (B) strains 1S3D6 and 2S2F4 with a transposon insertion in the 5' UTR region of the *nrdIEF* operon, (C) strain 1S2E5 with a transposon insertion in the 5'UTR region of *cshA,* between *cshA* and the *ddl/murF* operon and (D) strain 2S2G10 with a transposon insertion in the *ybbR* gene. Black arrows indicate location of predicted promoters and the red arrow heads indicate transposon insertion sites. The genes (not drawn to scale) are shown as gray arrows.

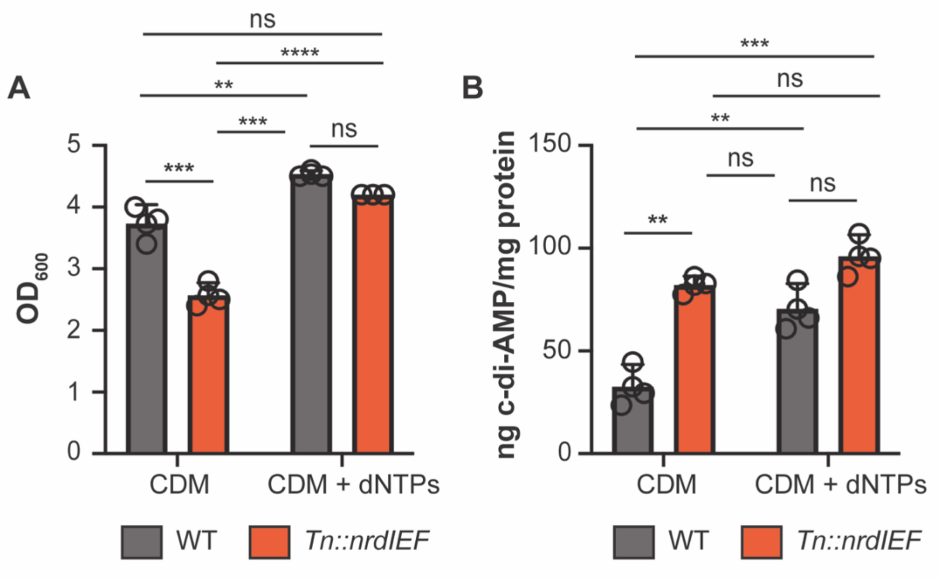

Figure S6. Deoxyribonucleotides restore the growth of the *Tn::nrdIEF* mutant and attenuate the effect of *nrdIEF* downregulation on c-di-AMP production. (A) OD_{600} values of WT LAC* and the *Tn::nrdIEF* mutant following 12h growth in CDM or CDM without 4 mM of dNTPs (B) Competitive ELISA to determine c-di-AMP levels in cell extracts. WT LAC* and the *Tn::nrdIEF* mutant were grown for 18h in CDM or CDM without 4 mM dNTPs, cell were extracts prepared and c-di-AMP levels determined by ELISA. The average and standard deviations of three biological replicates are plotted. Two-way ANOVA with Tukey's post hoc tests were performed to determine statistically significant differences between WT and the *Tn::nrdIEF* in either presence or absence of dNTPs. ** p value < 0.01**;** *** p value < 0.001, **** p value < 0.0001, ns indicates non-significant (p value> 0.05).

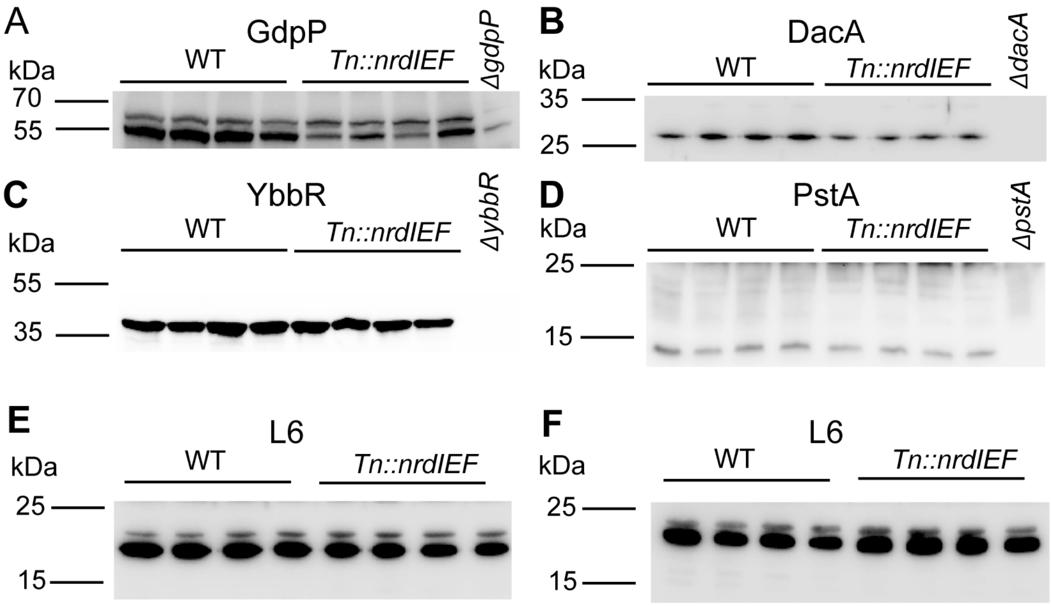

Figure S7. Comparison of GdpP, DacA, YbbR and PstA protein levels in WT and Tn::*nrdIEF* mutant strains. Western blotting for the detection of (A) GdpP, (B) DacA, (C) YbbR and (D) PstA, (E) L6 and (F) L6 from samples prepared using urea buffer. Samples were prepared from *S. aureu*s WT and the *Tn*::*nrdIEF* mutant and all samples were prepared from 4 biological replicates. The *dacA* mutant control strain for panel B was grown in the TSB supplemented with 0.6 M KCl.

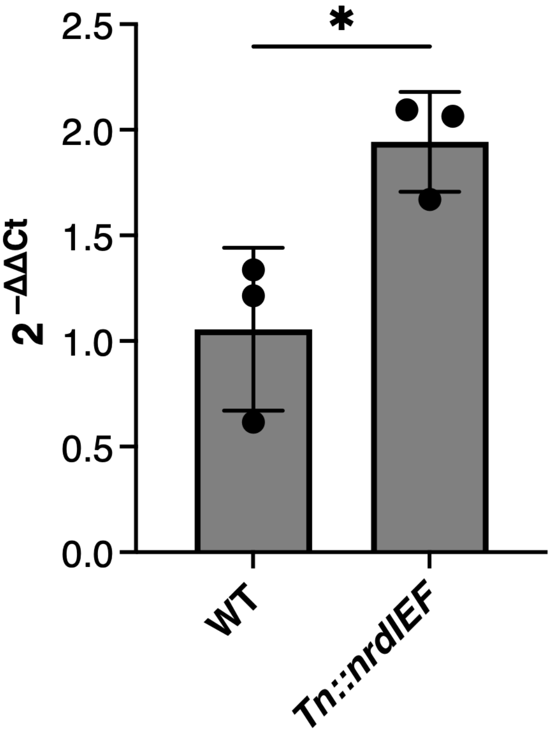

Figure S8. The transposon insertion in LAC* *Tn::nrdIEF* leads to increased expression of the *cshA* gene. qPCR analysis. RNA was extracted from log phase cultures of WT LAC* and the isogenic LAC* *Tn::nrdIEF* mutant strain following growth in TSB medium. The expression of the *cshA* gene normalized against the *gyrB* housekeeping gene was compared between the WT and the *Tn::nrdIEF* mutant by qPCR. The average and standard deviation from three samples is plotted and a t-test was used to determine statistically significant differences in *cshA* transcript levels between WT and the mutant strain. * indicates a p value < 0.05.

Supplemental text:

<u>Biosensor plasmid sequences</u>

pCN38
ATTCTGTGGATAACCGTATTACCGCCTTTGAGTGAGCTGGCGGCCGCTGCATGCCTGCAG
GTCGACTCTAGAGGATCCCCGGGTACCGAGCTCGAATTCACTGGCCGTCGTTTTACAACG
TCGTGACTGGGAAAACCCTGGCGTTACCCAACTTAATCGCCTTGCAGCACATCCCCCTTT
CGCCAGCTGGCGTAATAGCGAAGAGGCCCGCACCGATCGCCCTTCCCAACAGTTGCGC
AGCCTGAATGGCGAATGGCGCCTTTGCGGAAAGAGTTAATAAGTTAACAGAAGATGAACC
AAAACTAAATGGTTTAGCAGGAAACTTAGATAAAAAAATGAATCCAGAATTATATTCAGAACA
GGAACAGCAACAAGAACAACAAAAGAATCAAAAACGAGATAGAGGTATGCACTTATAGAA
CATGCATTTATGCCGAGAAAACTTATTGGTTGGAATGGGCTATGTGTTAGCTAACTTGTTAGC
GAGTTGGTTGGACTTGAATTGGGATTAATCCCAAGAAAGTACCAACTCAACAACACATAAA
GCCCTGTAGGTTCCGACCAATAAGGAAATTGGAATAAAGCAATAAAAGGAGTTGAAGAAAT
GAAATTCAGAGAAGCCTTTGAGAATTTTATAACAAGTAAGTATGTACTTGGTGTTTTAGTAGTT
TTAACTGTTTACCAGATAATACAAATGCTTAAATAAAAAAAGACTTGATCTGATTAGACCAAAT
CTTTTGATAGTGTTATATTAATAACAAAATAAAAAGGAGTCGCTCACGCCCTGACCAAAGTTT
GTGAACGACATCATTCAAAGAAAAAAACACTGAGTTGTTTTTATAATCTTGTATATTTAGATATT
AAACGATATTTAAATATACATCAAGATATATATTTGGGTGAGCGATTCCTTAAACGAAATTGAG
ATTAAGGAGTCGATTTTTTATGTATAAAAACAATCATGCAAATCATTCAAATCATTTGGAAAAT
CACGATTTAGACAATTTTTCTAAAACCGGCTACTCTAATAGCCGGTTGGACGCACATACTGT
GTGCATATCTGATCCAAAATTAAGTTTTGATGCAATGACGATCGTTGGAAATCTCAACCGAG
ACAACGCTCAAGCCCTTTCTAAATTTATGAGTGTAGAGCCCCAAATAAGACTTTGGGATATT
CTTCAAACAAAGTTTAAAGCTAAAGCACTTCAAGAAAAAGTTTATATTGAATATGACAAAGTG
AAAGCAGATAGTTGGGATAGACGTAATATGCGTATTGAATTTAATCCAAACAAACTTACACG
AGATGAAATGATTTGGTTAAAACAAAATATAATAAGCTACATGGAAGATGACGGTTTTACAAG
ATTAGATTTAGCCTTTGATTTTGAAGATGATTTGAGTGACTACTATGCAATGTCTGATAAAGCA
GTTAAGAAAACTATTTTTTATGGTCGTAATGGTAAGCCAGAAACAAAATATTTTGGCGTGAGA
GATAGTAATAGATTTATTAGAATTTATAATAAAAAGCAAGAACGTAAAGATAATGCAGATGCTG
AAGTTATGTCTGAACATTTATGGCGTGTAGAAATCGAACTTAAAAGAGATATGGTGGATTACT
GGAATGATTGCTTTAGTGATTTACATATCTTGCAACCAGATTGGAAAACTATCCAACGCACT
GCGGATAGAGCAATAGTTTTTATGTTATTGAGTGATGAAGAAGAATGGGGAAAGCTTCACAG
AAATTCTAGAACAAAATATAAGAATTTGATAAAAGAAATTTCGCCAGTCGATTTAACGGACTT
AATGAAATCGACTTTAAAAGCGAACGAAAAACAATTGCAAAAACAAATCGATTTTTGGCAAC
ATGAATTTAAATTTTGGAAATAGTGTACATATTAATATTACTGAACAAAAATGATATATTTAAACT
ATTCTAATTTAGGAGGATTTTTTTATGAAGTGTCTATTTAAAAATTTGGGGAATTTATATGAGGT
GAAAGAATAATTTACCCCTATAAACTTTAGTCACCTCAAGTAAAGAGGTAAAATTGTTTAGTTT
ATATAAAAAAATTTAAAGGTTTGTTTTATAGCGTTTTATTTTGGCTTTGTATTCTTTCATTTTTTAGTG
TATTAAATGAAATGGTTTTAAATGTTTCTTTACCTGATATTGCAAATCATTTTAATACTACTCCTG
GAATTACAAACTGGGTAAACACTGCATATATGTTAACTTTTTCGATAGGAACAGCAGTATATG
GAAAATTATCTGATTATATAAATATAAAAAAAATTGTTAATTATTGGTATTAGTTTGAGCTGTCTTG
GTTCATTGATTGCTTTTATTGGGCCCACCTAGGTATTATCAAGATAAGAAAGAAAAGGATTTTT
CGCTACGCTCAAATCCTTTAAAAAAACACAAAAGACCACATTTTTTAATGTGGTCTTTTATTCT
TCAACTAAAGCACCCATTAGTTCAACAAACGAAAATTGGATAAAGTGGGATATTTTTAAAATA
TATATTTATGTTACAGTAATATTGACTTTTAAAAAAGGATTGATTCTAATGAAGAAAGCAGACAA

GTAAGCCTCCTAAATTCACTTTAGATAAAAATTTAGGAGGCATATCAAATGAACTTTAATAAAA
TTGATTTAGACAATTGGAAGAGAAAAGAGATATTTAATCATTATTTGAACCAACAAACGACTTT
TAGTATAACCACAGAAATTGATATTAGTGTTTTATACCGAAACATAAAACAAGAAGGATATAA
ATTTTACCCTGCATTTATTTTCTTAGTGACAAGGGTGATAAACTCAAATACAGCTTTTAGAACT
GGTTACAATAGCGACGGAGAGTTAGGTTATTGGGATAAGTTAGAGCCACTTTATACAATTTTT
GATGGTGTATCTAAAACATTCTCTGGTATTTGGACTCCTGTAAAGAATGACTTCAAAGAGTTT
TATGATTTATACCTTTCTGATGTAGAGAAATATAATGGTTCGGGGAAATTGTTTCCCAAAACA
CCTATACCTGAAAATGCTTTTTCTCTTTCTATTATTCCATGGACTTCATTTACTGGGTTTAACTT
AAATATCAATAATAATAGTAATTACCTTCTACCCATTATTACAGCAGGAAAATTCATTAATAAA
GGTAATTCAATATATTTACCGCTATCTTTACAGGTACATCATTCTGTTTGTGATGGTTATCATG
CAGGATTGTTTATGAACTCTATTCAGGAATTGTCAGATAGGCCTAATGACTGGCTTTTATAAT
ATGAGATAATGCCGACTGTACTTTTTACAGTCGGTTTTCTAATGTCACTAACCTGCCCCGTTA
GTTGAAGAAGGCCGCGGCCTCGAGCGGCCGCATAGTTAAGCCAGCCCCGACACCCGC
CAACACCCGCTGACGCGCCCTGACGGGCTTGTCTGCTCCCGGCATCCGCTTACAGACA
AGCTGTGACCGTCTCCGGGAGCTGCATGTGTCAGAGGTTTTCACCGTCATCACCGAAACG
CGCGAGACGAAAGGGCCTCGTGATACGCCTATTTTTATAGGTTAATGTCATGATAATAATGG
TTTCTTAGACGTCAGGTGGCACTTTTCGGGGAAATGTGCGCGGAACCCCTATTTGTTTATTT
TTCTAAATACATTCAAATATGTATCCGCTCATGAGACAATAACCCTGATAAATGCTTCAATAAT
ATTGAAAAAGGAAGAGTATGAGTATTCAACATTTCCGTGTCGCCCTTATTCCCTTTTTTGCGG
CATTTTGCCTTCCTGTTTTTGCTCACCCAGAAACGCTGGTGAAAGTAAAAGATGCTGAAGAT
CAGTTGGGTGCACGAGTGGGTTACATCGAACTGGATCTCAACAGCGGTAAGATCCTTGAG
AGTTTTCGCCCCGAAGAACGTTTTCCAATGATGAGCACTTTTAAAGTTCTGCTATGTGGCGC
GGTATTATCCCGTATTGACGCCGGGCAAGAGCAACTCGGTCGCCGCATACACTATTCTCA
GAATGACTTGGTTGAGTACTCACCAGTCACAGAAAAGCATCTTACGGATGGCATGACAGTA
AGAGAATTATGCAGTGCTGCCATAACCATGAGTGATAACACTGCGGCCAACTTACTTCTGA
CAACGATCGGAGGACCGAAGGAGCTAACCGCTTTTTTGCACAACATGGGGGATCATGTAA
CTCGCCTTGATCGTTGGGAACCGGAGCTGAATGAAGCCATACCAAACGACGAGCGTGAC
ACCACGATGCCTGTAGCAATGGCAACAACGTTGCGCAAACTATTAACTGGCGAACTACTTA
CTCTAGCTTCCCGGCAACAATTAATAGACTGGATGGAGGCGGATAAAGTTGCAGGACCAC
TTCTGCGCTCGGCCCTTCCGGCTGGCTGGTTTATTGCTGATAAATCTGGAGCCGGTGAGC
GTGGGTCTCGCGGTATCATTGCAGCACTGGGGCCAGATGGTAAGCCCTCCCGTATCGTA
GTTATCTACACGACGGGGAGTCAGGCAACTATGGATGAACGAAATAGACAGATCGCTGAG
ATAGGTGCCTCACTGATTAAGCATTGGTAACTGTCAGACCAAGTTTACTCATATATACTTTAG
ATTGATTTAAAACTTCATTTTTAATTTAAAAGGATCTAGGTGAAGATCCTTTTTGATAATCTCATG
ACCAAAATCCCTTAACGTGAGTTTTCGTTCCACTGAGCGTCAGACCCCGTAGAAAAGATCA
AAGGATCTTCTTGAGATCCTTTTTTTCTGCGCGTAATCTGCTGCTTGCAAACAAAAAAACCA
CCGCTACCAGCGGTGGTTTGTTTGCCGGATCAAGAGCTACCAACTCTTTTTCCGAAGGTAA
CTGGCTTCAGCAGAGCGCAGATACCAAATACTGTTCTTCTAGTGTAGCCGTAGTTAGGCCA
CCACTTCAAGAACTCTGTAGCACCGCCTACATACCTCGCTCTGCTAATCCTGTTACCAGTG
GCTGCTGCCAGTGGCGATAAGTCGTGTCTTACCGGGTTGGACTCAAGACGATAGTTACCG
GATAAGGCGCAGCGGTCGGGCTGAACGGGGGGTTCGTGCACACAGCCCAGCTTGGAG
CGAACGACCTACACCGAACTGAGATACCTACAGCGTGAGCTATGAGAAAGCGCCACGCT
TCCCGAAGGGAGAAAGGCGGACAGGTATCCGGTAAGCGGCAGGGTCGGAACAGGAGA
GCGCACGAGGGAGCTTCCAGGGGGAAACGCCTGGTATCTTTATAGTCCTGTCGGGTTTC
GCCACCTCTGACTTGAGCGTCGATTTTTGTGATGCTCGTCAGGGGGGCGGAGCCTATGGA
AAAACGCCAGCAACGCGGCCTTTTTACGGTTCCTGGCCTTTTGCTGGCCTTTTGCTCACAT
GTTCTTTCCTGCGTTATCCCCTG

pCN38 *yfp*

ATTCTGTGGATAACCGTATTACCGCCTTTGAGTGAGCTGGCGGCCGCTGCATGCCTGCAG
GTCGACTCTAGAGGATCCGTAGCCCTTGCCTACCTAGCTTCCAAGAAAGATATCCTTACAG
CACAAGAGCGGAAAGATGTTTTGTTCTACATCCAGAACAACCTCTGCTAAAATTCCTGAAAA
ATTTTCGAAAAAGTTGTTGACTTTATCTACAAGGTGTGGCATAATGTGTGTGCATAATCAAAC
ACGAACGGGGGAACCAAAAGCTTAAGGAGGAAAGTCACATTATGAGCAAAGGTGAAGAA
CTGTTCACCGGCGTTGTGCCAATTCTGGTTGAGCTGGATGGTGACGTGAATGGCCACAAA
TTTTCCGTGTCTGGTGAAGGCGAGTGTGATGCTACTTATGGCAAACTGACTCTGAAACTGAT
CTGTACCACCGGCAAACTGCCTGTTCCGTGGCCAACTCTGGTCACTACTCTGGGTTACGG
CCTGATGTGTTTTGCGCGTTACCCGGATCACATGAAACAGCATGACTTCTTCAAATCTGCC
ATGCCGGAAGGCTATGTCCAAGAACGTACGATCTTTTTCAAGGACGACGGCAACTATAAAA
CCCGTGCCGAAGTTAAATTCGAGGGTGACACCCTGGTCAACCGCATCGAACTGAAAGGC
ATTGACTTCAAAGAGGACGGCAACATTCTGGGTCACAAGCTGGAATACAACTACAACTCC
CACAACGTTTACATTACTGCTGACAAGCAGAAAAACGGCATCAAAGCAAACTTCAAGATCC
GTCACAACATTGAAGATGGTGGCGTACAGCTGGCAGATCACTACCAGCAGAACACTCCAA
TCGGTGATGGCCCAGTACTGCTGCCAGATAACCATTACCTGTCCTACCAGAGCAAACTGT
CTAAAGACCCGAACGAAAAACGTGACCACATGGTACTGCTGGAATTTGTTACCGCGGCAG
GCATTACCCACGGTATGGACGAACTGTATAAATAAGCTAGCAAAAACCCCGCCCCTGACA
GGGCGGGGTTTTTTTGAATTCACTGGCCGTCGTTTTACAACGTCGTGACTGGGAAAACCCT
GGCGTTACCCAACTTAATCGCCTTGCAGCACATCCCCCTTTCGCCAGCTGGCGTAATAGC
GAAGAGGCCCGCACCGATCGCCCTTCCCAACAGTTGCGCAGCCTGAATGGCGAATGGC
GCCTTTGCGGAAAGAGTTAATAAGTTAACAGAAGATGAACCAAAACTAAATGGTTTAGCAGG
AAACTTAGATAAAAAAATGAATCCAGAATTATATTCAGAACAGGAACAGCAACAAGAACAAC
AAAAGAATCAAAAACGAGATAGAGGTATGCACTTATAGAACATGCATTTATGCCGAGAAAAC
TTATTGGTTGGAATGGGCTATGTGTTAGCTAACTTGTTAGCGAGTTGGTTGGACTTGAATTGG
GATTAATCCCAAGAAAGTACCAACTCAACAACACATAAAGCCCTGTAGGTTCCGACCAATA
AGGAAATTGGAATAAAGCAATAAAAGGAGTTGAAGAAATGAAATTCAGAGAAGCCTTTGAGA
ATTTTATAACAAGTAAGTATGTACTTGGTGTTTTAGTAGTTTTAACTGTTTACCAGATAATACAA
ATGCTTAAATAAAAAAAGACTTGATCTGATTAGACCAAATCTTTTGATAGTGTTATATTAATAAC
AAAATAAAAAGGAGTCGCTCACGCCCTGACCAAAGTTTGTGAACGACATCATTCAAAGAAA
AAAACACTGAGTTGTTTTTATAATCTTGTATATTTAGATATTAAACGATATTTAAATATACATCAA
GATATATATTTGGGTGAGCGATTCCTTAAACGAAATTGAGATTAAGGAGTCGATTTTTTATGTA
TAAAAACAATCATGCAAATCATTCAAATCATTTGGAAAATCACGATTTAGACAATTTTTCTAAA
ACCGGCTACTCTAATAGCCGGTTGGACGCACATACTGTGTGCATATCTGATCCAAAATTAA
GTTTTGATGCAATGACGATCGTTGGAAATCTCAACCGAGACAACGCTCAAGCCCTTTCTAA
ATTTATGAGTGTAGAGCCCCAAATAAGACTTTGGGATATTCTTCAAACAAAGTTTAAAGCTAA
AGCACTTCAAGAAAAAGTTTATATTGAATATGACAAAGTGAAAGCAGATAGTTGGGATAGAC
GTAATATGCGTATTGAATTTAATCCAAACAAACTTACACGAGATGAAATGATTTGGTTAAAAC
AAAATATAATAAGCTACATGGAAGATGACGGTTTTACAAGATTAGATTTAGCCTTTGATTTTGA
AGATGATTTGAGTGACTACTATGCAATGTCTGATAAAGCAGTTAAGAAAACTATTTTTTATGGT
CGTAATGGTAAGCCAGAAACAAAATATTTTGGCGTGAGAGATAGTAATAGATTTATTAGAATT
TATAATAAAAAGCAAGAACGTAAAGATAATGCAGATGCTGAAGTTATGTCTGAACATTTATGG
CGTGTAGAAATCGAACTTAAAAGAGATATGGTGGATTACTGGAATGATTGCTTTAGTGATTTA
CATATCTTGCAACCAGATTGGAAAACTATCCAACGCACTGCGGATAGAGCAATAGTTTTTAT
GTTATTGAGTGATGAAGAAGAATGGGGAAAGCTTCACAGAAATTCTAGAACAAAATATAAGA
ATTTGATAAAAGAAATTTCGCCAGTCGATTTAACGGACTTAATGAAATCGACTTTAAAAGCGA

ACGAAAAACAATTGCAAAAACAAATCGATTTTTGGCAACATGAATTTAAATTTTGGAAATAGT
GTACATATTAATATTACTGAACAAAAATGATATATTTAAACTATTCTAATTTAGGAGGATTTTTTT
ATGAAGTGTCTATTTAAAAATTTGGGGAATTTATATGAGGTGAAAGAATAATTTACCCCTATAA
ACTTTAGTCACCTCAAGTAAAGAGGTAAAATTGTTTAGTTTATATAAAAAATTTAAAGGTTTGTT
TTATAGCGTTTTATTTTGGCTTTGTATTCTTTCATTTTTTAGTGTATTAAATGAAATGGTTTTAAAT
GTTTCTTTACCTGATATTGCAAATCATTTTAATACTACTCCTGGAATTACAAACTGGGTAAACA
CTGCATATATGTTAACTTTTTCGATAGGAACAGCAGTATATGGAAAATTATCTGATTATATAAAT
ATAAAAAAATTGTTAATTATTGGTATTAGTTTGAGCTGTCTTGGTTCATTGATTGCTTTTATTGGG
CCCACCTAGGAATTATCAAGATAAGAAAGAAAAGGATTTTTCGCTACGCTCAAATCCTTTAA
AAAAACACAAAAGACCACATTTTTTAATGTGGTCTTTTATTCTTCAACTAAAGCACCCATTAGT
TCAACAAACGAAAATTGGATAAAGTGGGATATTTTTAAAATATATATTTATGTTACAGTAATATT
GACTTTTAAAAAAGGATTGATTCTAATGAAGAAAGCAGACAAGTAAGCCTCCTAAATTCACTT
TAGATAAAAATTTAGGAGGCATATCAAATGAACTTTAATAAAATTGATTTAGACAATTGGAAGA
GAAAAGAGATATTTAATCATTATTTGAACCAACAAACGACTTTTAGTATAACCACAGAAATTGA
TATTAGTGTTTTATACCGAAACATAAAACAAGAAGGATATAAATTTTACCCTGCATTTATTTTCT
TAGTGACAAGGGTGATAAACTCAAATACAGCTTTTAGAACTGGTTACAATAGCGACGGAGA
GTTAGGTTATTGGGATAAGTTAGAGCCACTTTATACAATTTTTGATGGTGTATCTAAAACATTC
TCTGGTATTTGGACTCCTGTAAAGAATGACTTCAAAGAGTTTTATGATTTATACCTTTCTGATG
TAGAGAAATATAATGGTTCGGGGAAATTGTTTCCCAAAACACCTATACCTGAAAATGCTTTTT
CTCTTTCTATTATTCCATGGACTTCATTTACTGGGTTTAACTTAAATATCAATAATAATAGTAATT
ACCTTCTACCCATTATTACAGCAGGAAAATTCATTAATAAAGGTAATTCAATATATTTACCGCT
ATCTTTACAGGTACATCATTCTGTTTGTGATGGTTATCATGCAGGATTGTTTATGAACTCTATT
CAGGAATTGTCAGATAGGCCTAATGACTGGCTTTTATAATATGAGATAATGCCGACTGTACT
TTTTACAGTCGGTTTTCTAATGTCACTAACCTGCCCCGTTAGTTGAAGAAGTCCGCGGCCT
CGAGCGGCCGCATAGTTAAGCCAGCCCCGACACCCGCCAACACCCGCTGACGCGCCC
TGACGGGCTTGTCTGCTCCCGGCATCCGCTTACAGACAAGCTGTGACCGTCTCCGGGAG
CTGCATGTGTCAGAGGTTTTCACCGTCATCACCGAAACGCGCGAGACGAAAGGGCCTCG
TGATACGCCTATTTTTATAGGTTAATGTCATGATAATAATGGTTTCTTAGACGTCAGGTGGCA
CTTTTCGGGGAAATGTGCGCGGAACCCCTATTTGTTTATTTTTCTAAATACATTCAAATATGTA
TCCGCTCATGAGACAATAACCCTGATAAATGCTTCAATAATATTGAAAAAGGAAGAGTATGA
GTATTCAACATTTCCGTGTCGCCCTTATTCCCTTTTTTGCGGCATTTTGCCTTCCTGTTTTTGC
TCACCCAGAAACGCTGGTGAAAGTAAAAGATGCTGAAGATCAGTTGGGTGCACGAGTGGG
TTACATCGAACTGGATCTCAACAGCGGTAAGATCCTTGAGAGTTTTCGCCCCGAAGAACGT
TTTCCAATGATGAGCACTTTTAAAGTTCTGCTATGTGGCGCGGTATTATCCCGTATTGACGC
CGGGCAAGAGCAACTCGGTCGCCGCATACACTATTCTCAGAATGACTTGGTTGAGTACTC
ACCAGTCACAGAAAAGCATCTTACGGATGGCATGACAGTAAGAGAATTATGCAGTGCTGC
CATAACCATGAGTGATAACACTGCGGCCAACTTACTTCTGACAACGATCGGAGGACCGAA
GGAGCTAACCGCTTTTTTGCACAACATGGGGGATCATGTAACTCGCCTTGATCGTTGGGAA
CCGGAGCTGAATGAAGCCATACCAAACGACGAGCGTGACACCACGATGCCTGTAGCAAT
GGCAACAACGTTGCGCAAACTATTAACTGGCGAACTACTTACTCTAGCTTCCCGGCAACA
ATTAATAGACTGGATGGAGGCGGATAAAGTTGCAGGACCACTTCTGCGCTCGGCCCTTCC
GGCTGGCTGGTTTATTGCTGATAAATCTGGAGCCGGTGAGCGTGGGTCTCGCGGTATCATT
GCAGCACTGGGGCCAGATGGTAAGCCCTCCCGTATCGTAGTTATCTACACGACGGGGAG
TCAGGCAACTATGGATGAACGAAATAGACAGATCGCTGAGATAGGTGCCTCACTGATTAAG
CATTGGTAACTGTCAGACCAAGTTTACTCATATATACTTTAGATTGATTTAAAACTTCATTTTTA
ATTTAAAAGGATCTAGGTGAAGATCCTTTTTGATAATCTCATGACCAAAATCCCTTAACGTGA
GTTTTCGTTCCACTGAGCGTCAGACCCCGTAGAAAAGATCAAAGGATCTTCTTGAGATCCT

TTTTTTCTGCGCGTAATCTGCTGCTTGCAAACAAAAAAACCACCGCTACCAGCGGTGGTTT
GTTTGCCGGATCAAGAGCTACCAACTCTTTTTCCGAAGGTAACTGGCTTCAGCAGAGCGC
AGATACCAAATACTGTTCTTCTAGTGTAGCCGTAGTTAGGCCACCACTTCAAGAACTCTGTA
GCACCGCCTACATACCTCGCTCTGCTAATCCTGTTACCAGTGGCTGCTGCCAGTGGCGAT
AAGTCGTGTCTTACCGGGTTGGACTCAAGACGATAGTTACCGGATAAGGCGCAGCGGTC
GGGCTGAACGGGGGGTTCGTGCACACAGCCCAGCTTGGAGCGAACGACCTACACCGAA
CTGAGATACCTACAGCGTGAGCTATGAGAAAGCGCCACGCTTCCCGAAGGGAGAAAGGC
GGACAGGTATCCGGTAAGCGGCAGGGTCGGAACAGGAGAGCGCACGAGGGAGCTTCC
AGGGGGAAACGCCTGGTATCTTTATAGTCCTGTCGGGTTTCGCCACCTCTGACTTGAGCG
TCGATTTTTGTGATGCTCGTCAGGGGGGCGGAGCCTATGGAAAAACGCCAGCAACGCGG
CCTTTTTACGGTTCCTGGCCTTTTGCTGGCCTTTTGCTCACATGTTCTTTCCTGCGTTATCCC
CTG

pCN38 *ktrA-yfp*

ATTCTGTGGATAACCGTATTACCGCCTTTGAGTGAGCTGGCGGCCGCTGCATGCCTGCAG
GTCGACTCTAGAGGATCCGTAGCCCTTGCCTACCTAGCTTCCAAGAAAGATATCCTTACAG
CACAAGAGCGGAAAGATGTTTTGTTCTACATCCAGAACAACCTCTGCTAAAATTCCTGAAAA
ATTTTCGAAAAAGTTGTTGACTTTATCTACAAGGTGTGGCATAATGTGTGTGCATAATCAAAC
ACGAACGGGGGAACCAACGATTGGCTGTTTTATTAACAGCCTTGGGGTGAATCTTACTAAG
TAAGAGGGGGTACTCTGAATCCCTAATCCGACAGCTAACCTCGTAGGCGTATACAGAGAG
GAGGTCTATTATCGGCCATTCTTTGCAATGGTCTTTTTTGTTTCAAAAACGTTAATTCACTAAA
GCTTAAGGAGGAAAGTCACATTATGAGCAAAGGTGAAGAACTGTTCACCGGCGTTGTGCC
AATTCTGGTTGAGCTGGATGGTGACGTGAATGGCCACAAATTTTCCGTGTCTGGTGAAGGC
GAGTGTGATGCTACTTATGGCAAACTGACTCTGAAACTGATCTGTACCACCGGCAAACTGC
CTGTTCCGTGGCCAACTCTGGTCACTACTCTGGGTTACGGCCTGATGTGTTTTGCGCGTTA
CCCGGATCACATGAAACAGCATGACTTCTTCAAATCTGCCATGCCGGAAGGCTATGTCCA
AGAACGTACGATCTTTTTCAAGGACGACGGCAACTATAAAACCCGTGCCGAAGTTAAATTC
GAGGGTGACACCCTGGTCAACCGCATCGAACTGAAAGGCATTGACTTCAAAGAGGACGG
CAACATTCTGGGTCACAAGCTGGAATACAACTACAACTCCCACAACGTTTACATTACTGCT
GACAAGCAGAAAAACGGCATCAAAGCAAACTTCAAGATCCGTCACAACATTGAAGATGGT
GGCGTACAGCTGGCAGATCACTACCAGCAGAACACTCCAATCGGTGATGGCCCAGTACT
GCTGCCAGATAACCATTACCTGTCCTACCAGAGCAAACTGTCTAAAGACCCGAACGAAAA
ACGTGACCACATGGTACTGCTGGAATTTGTTACCGCGGCAGGCATTACCCACGGTATGGA
CGAACTGTATAAATAAGCTAGCAAAAACCCCGCCCCTGACAGGGCGGGGTTTTTTTGAATT
CACTGGCCGTCGTTTTACAACGTCGTGACTGGGAAAACCCTGGCGTTACCCAACTTAATC
GCCTTGCAGCACATCCCCCTTTCGCCAGCTGGCGTAATAGCGAAGAGGCCCGCACCGA
TCGCCCTTCCCAACAGTTGCGCAGCCTGAATGGCGAATGGCGCCTTTGCGGAAAGAGTT
AATAAGTTAACAGAAGATGAACCAAAACTAAATGGTTTAGCAGGAAACTTAGATAAAAAAAT
GAATCCAGAATTATATTCAGAACAGGAACAGCAACAAGAACAACAAAAGAATCAAAAACGA
GATAGAGGTATGCACTTATAGAACATGCATTTATGCCGAGAAAACTTATTGGTTGGAATGGG
CTATGTGTTAGCTAACTTGTTAGCGAGTTGGTTGGACTTGAATTGGGATTAATCCCAAGAAA
GTACCAACTCAACAACACATAAAGCCCTGTAGGTTCCGACCAATAAGGAAATTGGAATAAA
GCAATAAAAGGAGTTGAAGAAATGAAATTCAGAGAAGCCTTTGAGAATTTTATAACAAGTAA
GTATGTACTTGGTGTTTTAGTAGTTTTAACTGTTTACCAGATAATACAAATGCTTAAATAAAAAA
AGACTTGATCTGATTAGACCAAATCTTTTGATAGTGTTATATTAATAACAAAATAAAAAGGAGT
CGCTCACGCCCTGACCAAAGTTTGTGAACGACATCATTCAAAGAAAAAAACACTGAGTTGT
TTTTATAATCTTGTATATTTAGATATTAAACGATATTTAAATATACATCAAGATATATATTTGGGTG
AGCGATTCCTTAAACGAAATTGAGATTAAGGAGTCGATTTTTTATGTATAAAAACAATCATGC
AAATCATTCAAATCATTTGGAAAATCACGATTTAGACAATTTTTCTAAAACCGGCTACTAATAG
CCGGTTGGACGCACATACTGTGTGCATATCTGATCCAAAATTAAGTTTTGATGCAATGACGA
TCGTTGGAAATCTCAACCGAGACAACGCTCAAGCCCTTTCTAAATTTATGAGTGTAGAGCC
CCAAATAAGACTTTGGGATATTCTTCAAACAAAGTTTAAAGCTAAAGCACTTCAAGAAAAAGT
TTATATTGAATATGACAAAGTGAAAGCAGATAGTTGGGATAGACGTAATATGCGTATTGAATTT
AATCCAAACAAACTTACACGAGATGAAATGATTTGGTTAAAACAAAATATAATAAGCTACATG
GAAGATGACGGTTTTACAAGATTAGATTTAGCCTTTGATTTTGAAGATGATTTGAGTGACTACT
ATGCAATGTCTGATAAAGCAGTTAAGAAAACTATTTTTTATGGTCGTAATGGTAAGCCAGAAA
CAAAATATTTTGGCGTGAGAGATAGTAATAGATTTATTAGAATTTATAATAAAAAGCAAGAACG
TAAAGATAATGCAGATGCTGAAGTTATGTCTGAACATTTATGGCGTGTAGAAATCGAACTTAA
AAGAGATATGGTGGATTACTGGAATGATTGCTTTAGTGATTTACATATCTTGCAACCAGATTG
GAAAACTATCCAACGCACTGCGGATAGAGCAATAGTTTTTATGTTATTGAGTGATGAAGAAG

AATGGGGAAAGCTTCACAGAAATTCTAGAACAAAATATAAGAATTTGATAAAAGAAATTTCGC
CAGTCGATTTAACGGACTTAATGAAATCGACTTTAAAAGCGAACGAAAAACAATTGCAAAAA
CAAATCGATTTTTGGCAACATGAATTTAAATTTTGGAAATAGTGTACATATTAATATTACTGAAC
AAAAATGATATATTTAAACTATTCTAATTTAGGAGGATTTTTTTATGAAGTGTCTATTTAAAAATTT
GGGGAATTTATATGAGGTGAAAGAATAATTTACCCCTATAAACTTTAGTCACCTCAAGTAAAG
AGGTAAAATTGTTTAGTTTATATAAAAAATTTAAAGGTTTGTTTTATAGCGTTTTATTTTGGCTTTG
TATTCTTTCATTTTTTAGTGTATTAAATGAAATGGTTTTAAATGTTTCTTTACCTGATATTGCAAAT
CATTTTAATACTACTCCTGGAATTACAAACTGGGTAAACACTGCATATATGTTAACTTTTTCGA
TAGGAACAGCAGTATATGGAAAATTATCTGATTATATAAATATAAAAAAATTGTTAATTATTGGT
ATTAGTTTGAGCTGTCTTGGTTCATTGATTGCTTTTATTGGGCCCACCTAGGTATTATCAAGAT
AAGAAAGAAAAGGATTTTTCGCTACGCTCAAATCCTTTAAAAAAACACAAAAGACCACATTTT
TTAATGTGGTCTTTTATTCTTCAACTAAAGCACCCATTAGTTCAACAAACGAAAATTGGATAAA
GTGGGATATTTTTAAAATATATATTTATGTTACAGTAATATTGACTTTTAAAAAAGGATTGATTCT
AATGAAGAAAGCAGACAAGTAAGCCTCCTAAATTCACTTTAGATAAAAATTTAGGAGGCATA
TCAAATGAACTTTAATAAAATTGATTTAGACAATTGGAAGAGAAAAGAGATATTTAATCATTATT
TGAACCAACAAACGACTTTTAGTATAACCACAGAAATTGATATTAGTGTTTTATACCGAAACA
TAAAACAAGAAGGATATAAATTTTACCCTGCATTTATTTTCTTAGTGACAAGGGTGATAAACTC
AAATACAGCTTTTAGAACTGGTTACAATAGCGACGGAGAGTTAGGTTATTGGGATAAGTTAG
AGCCACTTTATACAATTTTTGATGGTGTATCTAAAACATTCTCTGGTATTTGGACTCCTGTAAA
GAATGACTTCAAAGAGTTTTATGATTTATACCTTTCTGATGTAGAGAAATATAATGGTTCGGGG
AAATTGTTTCCCAAAACACCTATACCTGAAAATGCTTTTTCTCTTTCTATTATTCCATGGACTT
CATTTACTGGGTTTAACTTAAATATCAATAATAATAGTAATTACCTTCTACCCATTATTACAGCA
GGAAAATTCATTAATAAAGGTAATTCAATATATTTACCGCTATCTTTACAGGTACATCATTCTG
TTTGTGATGGTTATCATGCAGGATTGTTTATGAACTCTATTCAGGAATTGTCAGATAGGCCTA
ATGACTGGCTTTTATAATATGAGATAATGCCGACTGTACTTTTTACAGTCGGTTTTCTAATGTC
ACTAACCTGCCCCGTTAGTTGAAGAAGGCCGCGGCCTCGAGCGGCCGCATAGTTAAGC
CAGCCCCGACACCCGCCAACACCCGCTGACGCGCCCTGACGGGCTTGTCTGCTCCCG
GCATCCGCTTACAGACAAGCTGTGACCGTCTCCGGGAGCTGCATGTGTCAGAGGTTTTCA
CCGTCATCACCGAAACGCGCGAGACGAAAGGGCCTCGTGATACGCCTATTTTTATAGGTT
AATGTCATGATAATAATGGTTTCTTAGACGTCAGGTGGCACTTTTCGGGGAAATGTGCGCG
GAACCCCTATTTGTTTATTTTTCTAAATACATTCAAATATGTATCCGCTCATGAGACAATAACC
CTGATAAATGCTTCAATAATATTGAAAAAGGAAGAGTATGAGTATTCAACATTTCCGTGTCGC
CCTTATTCCCTTTTTTGCGGCATTTTGCCTTCCTGTTTTTGCTCACCCAGAAACGCTGGTGAA
AGTAAAAGATGCTGAAGATCAGTTGGGTGCACGAGTGGGTTACATCGAACTGGATCTCAAC
AGCGGTAAGATCCTTGAGAGTTTTCGCCCCGAAGAACGTTTTCCAATGATGAGCACTTTTAA
AGTTCTGCTATGTGGCGCGGTATTATCCCGTATTGACGCCGGGCAAGAGCAACTCGGTCG
CCGCATACACTATTCTCAGAATGACTTGGTTGAGTACTCACCAGTCACAGAAAAGCATCTTA
CGGATGGCATGACAGTAAGAGAATTATGCAGTGCTGCCATAACCATGAGTGATAACACTG
CGGCCAACTTACTTCTGACAACGATCGGAGGACCGAAGGAGCTAACCGCTTTTTTGCACA
ACATGGGGGATCATGTAACTCGCCTTGATCGTTGGGAACCGGAGCTGAATGAAGCCATAC
CAAACGACGAGCGTGACACCACGATGCCTGTAGCAATGGCAACAACGTTGCGCAAACTA
TTAACTGGCGAACTACTTACTCTAGCTTCCCGGCAACAATTAATAGACTGGATGGAGGCGG
ATAAAGTTGCAGGACCACTTCTGCGCTCGGCCCTTCCGGCTGGCTGGTTTATTGCTGATAA
ATCTGGAGCCGGTGAGCGTGGGTCTCGCGGTATCATTGCAGCACTGGGGCCAGATGGTA
AGCCCTCCCGTATCGTAGTTATCTACACGACGGGGAGTCAGGCAACTATGGATGAACGAA
ATAGACAGATCGCTGAGATAGGTGCCTCACTGATTAAGCATTGGTAACTGTCAGACCAAGT
TTACTCATATATACTTTAGATTGATTTAAAACTTCATTTTTAATTTAAAAGGATCTAGGTGAAGAT

CCTTTTTGATAATCTCATGACCAAAATCCCTTAACGTGAGTTTTCGTTCCACTGAGCGTCAG
ACCCCGTAGAAAAGATCAAAGGATCTTCTTGAGATCCTTTTTTTCTGCGCGTAATCTGCTGC
TTGCAAACAAAAAAACCACCGCTACCAGCGGTGGTTTGTTTGCCGGATCAAGAGCTACCA
ACTCTTTTTCCGAAGGTAACTGGCTTCAGCAGAGCGCAGATACCAAATACTGTTCTTCTAGT
GTAGCCGTAGTTAGGCCACCACTTCAAGAACTCTGTAGCACCGCCTACATACCTCGCTCT
GCTAATCCTGTTACCAGTGGCTGCTGCCAGTGGCGATAAGTCGTGTCTTACCGGGTTGGA
CTCAAGACGATAGTTACCGGATAAGGCGCAGCGGTCGGGCTGAACGGGGGGTTCGTGC
ACACAGCCCAGCTTGGAGCGAACGACCTACACCGAACTGAGATACCTACAGCGTGAGCT
ATGAGAAAGCGCCACGCTTCCCGAAGGGAGAAAGGCGGACAGGTATCCGGTAAGCGGC
AGGGTCGGAACAGGAGAGCGCACGAGGGAGCTTCCAGGGGGAAACGCCTGGTATCTTT
ATAGTCCTGTCGGGTTTCGCCACCTCTGACTTGAGCGTCGATTTTTGTGATGCTCGTCAGG
GGGGCGGAGCCTATGGAAAAACGCCAGCAACGCGGCCTTTTTACGGTTCCTGGCCTTTT
GCTGGCCTTTTGCTCACATGTTCTTTCCTGCGTTATCCCCTG

pCN38 *kimA-yfp*

ATTCTGTGGATAACCGTATTACCGCCTTTGAGTGAGCTGGCGGCCGCTGCATGCCTGCAG
GTCGACTCTAGAGGATCCGTAGCCCTTGCCTACCTAGCTTCCAAGAAAGATATCCTTACAG
CACAAGAGCGGAAAGATGTTTTGTTCTACATCCAGAACAACCTCTGCTAAAATTCCTGAAAA
ATTTTCGAAAAAGTTGTTGACTTTATCTACAAGGTGTGGCATAATGTGTGTGCAGAAAACAAA
TCGCTTAATCTGAAATCAGAGCGGGGGACCCAATAGAACGGCTTTTTGCCGTTGGGGTGA
ATCCTTTTTAGGTAGGGCTAACTCTCATATGCCCGAATCCGTCAGCTAACCTCGTAAGCGT
TCGTGAGAGGAGATGAATGAAACCTGTGTTCGATGTTATGGCACAGGGGCATCCGTTTGC
CTCTGTGTTTTTTGTTGTTCATTTTTGAATGCAAATCGCATACAGAGACATCATAGCAGAAAG
GACTATCATCAACATGTGAGAGCAAGACACTGATGGATGTTTTATTGTTTTAATTGAAAAATG
AGAAATTAAAAGCTTAAGGAGGAAAGTCACATTATGAGCAAAGGTGAAGAACTGTTCACCG
GCGTTGTGCCAATTCTGGTTGAGCTGGATGGTGACGTGAATGGCCACAAATTTTCCGTGTC
TGGTGAAGGCGAGTGTGATGCTACTTATGGCAAACTGACTCTGAAACTGATCTGTACCACC
GGCAAACTGCCTGTTCCGTGGCCAACTCTGGTCACTACTCTGGGTTACGGCCTGATGTGT
TTTGCGCGTTACCCGGATCACATGAAACAGCATGACTTCTTCAAATCTGCCATGCCGGAAG
GCTATGTCCAAGAACGTACGATCTTTTTCAAGGACGACGGCAACTATAAAACCCGTGCCG
AAGTTAAATTCGAGGGTGACACCCTGGTCAACCGCATCGAACTGAAAGGCATTGACTTCA
AAGAGGACGGCAACATTCTGGGTCACAAGCTGGAATACAACTACAACTCCCACAACGTTT
ACATTACTGCTGACAAGCAGAAAAACGGCATCAAAGCAAACTTCAAGATCCGTCACAACAT
TGAAGATGGTGGCGTACAGCTGGCAGATCACTACCAGCAGAACACTCCAATCGGTGATG
GCCCAGTACTGCTGCCAGATAACCATTACCTGTCCTACCAGAGCAAACTGTCTAAAGACC
CGAACGAAAAACGTGACCACATGGTACTGCTGGAATTTGTTACCGCGGCAGGCATTACCC
ACGGTATGGACGAACTGTATAAATAAGCTAGCAAAAACCCCGCCCCTGACAGGGCGGGG
TTTTTTTGAATTCACTGGCCGTCGTTTTACAACGTCGTGACTGGGAAAACCCTGGCGTTACC
CAACTTAATCGCCTTGCAGCACATCCCCCTTTCGCCAGCTGGCGTAATAGCGAAGAGGC
CCGCACCGATCGCCCTTCCCAACAGTTGCGCAGCCTGAATGGCGAATGGCGCCTTTGC
GGAAAGAGTTAATAAGTTAACAGAAGATGAACCAAAACTAAATGGTTTAGCAGGAAACTTAG
ATAAAAAAATGAATCCAGAATTATATTCAGAACAGGAACAGCAACAAGAACAACAAAAGAAT
CAAAAACGAGATAGAGGTATGCACTTATAGAACATGCATTTATGCCGAGAAAACTTATTGGT
TGGAATGGGCTATGTGTTAGCTAACTTGTTAGCGAGTTGGTTGGACTTGAATTGGGATTAATC
CCAAGAAAGTACCAACTCAACAACACATAAAGCCCTGTAGGTTCCGACCAATAAGGAAATT
GGAATAAAGCAATAAAAGGAGTTGAAGAAATGAAATTCAGAGAAGCCTTTGAGAATTTTATAA
CAAGTAAGTATGTACTTGGTGTTTTAGTAGTTTTAACTGTTTACCAGATAATACAAATGCTTAAA
TAAAAAAAGACTTGATCTGATTAGACCAAATCTTTTGATAGTGTTATATTAATAACAAAATAAAA
AGGAGTCGCTCACGCCCTGACCAAAGTTTGTGAACGACATCATTCAAAGAAAAAAACACT
GAGTTGTTTTTATAATCTTGTATATTTAGATATTAAACGATATTTAAATATACATCAAGATATATAT
TTGGGTGAGCGATTCCTTAAACGAAATTGAGATTAAGGAGTCGATTTTTTATGTATAAAAACA
ATCATGCAAATCATTCAAATCATTTGGAAAATCACGATTTAAAATTTAAAGGTTTGTTTTATAGC
GTTTTATTTTGGCTTTGTATTCTTTCATTTTTTAGTGTATTAAATGAAATGGTTTTAAATGTTTCTTT
ACCTGATATTGCAAATCATTTTAATACTACTCCTGGAATTACAAACTGGGTAAACACTGCATA
TATGTTAACTTTTTCGATAGGAACAGCAGTATATGGAAAATTATCTGATTATATAAATATAAAAA
AATTGTTAATTATTGGTATTAGTTTGAGCTGTCTTGGTTCATTGATTGCTTTTATTGGGCCCACC
TAGGTATTATCAAGATAAGAAAGAAAAGGATTTTTCGCTACGCTCAAATCCTTTAAAAAAACA
CAAAAGACCACATTTTTTAATGTGGTCTTTTATTCTTCAACTAAAGCACCCATTAGTTCAACAA
ACGAAAATTGGATAAAGTGGGATATTTTTAAAATATATATTTATGTTACAGTAATATTGACTTTTA
AAAAAGGATTGATTCTAATGAAGAAAGCAGACAAGTAAGCCTCCTAAATTCACTTTAGATAAA
AATTTAGGAGGCATATCAAATGAACTTTAATAAAATTGATTTAGACAATTGGAAGAGAAAAGA

GATATTTAATCATTATTTGAACCAACAAACGACTTTTAGTATAACCACAGAAATTGATATTAGT
GTTTTATACCGAAACATAAAACAAGAAGGATATAAATTTTACCCTGCATTTATTTTCTTAGTGA
CAAGGGTGATAAACTCAAATACAGCTTTTAGAACTGGTTACAATAGCGACGGAGAGTTAGG
TTATTGGGATAAGTTAGAGCCACTTTATACAATTTTTGATGGTGTATCTAAAACATTCTCTGGT
ATTTGGACTCCTGTAAAGAATGACTTCAAAGAGTTTTATGATTTATACCTTTCTGATGTAGAGA
AATATAATGGTTCGGGGAAATTGTTTCCCAAAACACCTATACCTGAAAATGCTTTTTCTCTTTC
TATTATTCCATGGACTTCATTTACTGGGTTTAACTTAAATATCAATAATAATAGTAATTACCTTCT
ACCCATTATTACAGCAGGAAAATTCATTAATAAAGGTAATTCAATATATTTACCGCTATCTTTA
CAGGTACATCATTCTGTTTGTGATGGTTATCATGCAGGATTGTTTATGAACTCTATTCAGGAAT
TGTCAGATAGGCCTAATGACTGGCTTTTATAATATGAGATAATGCCGACTGTACTTTTTACAG
TCGGTTTTCTAATGTCACTAACCTGCCCCGTTAGTTGAAGAAGGCCGCGGCCTCGAGCGG
CCGCATAGTTAAGCCAGCCCCGACACCCGCCAACACCCGCTGACGCGCCCTGACGGG
CTTGTCTGCTCCCGGCATCCGCTTACAGACAAGCTGTGACCGTCTCCGGGAGCTGCATG
TGTCAGAGGTTTTCACCGTCATCACCGAAACGCGCGAGACGAAAGGGCCTCGTGATACG
CCTATTTTTATAGGTTAATGTCATGATAATAATGGTTTCTTAGACGTCAGGTGGCACTTTTCGG
GGAAATGTGCGCGGAACCCCTATTTGTTTATTTTTCTAAATACATTCAAATATGTATCCGCTC
ATGAGACAATAACCCTGATAAATGCTTCAATAATATTGAAAAAGGAAGAGTATGAGTATTCAA
CATTTCCGTGTCGCCCTTATTCCCTTTTTTGCGGCATTTTGCCTTCCTGTTTTTGCTCACCCA
GAAACGCTGGTGAAAGTAAAAGATGCTGAAGATCAGTTGGGTGCACGAGTGGGTTACATC
GAACTGGATCTCAACAGCGGTAAGATCCTTGAGAGTTTTCGCCCCGAAGAACGTTTTCCAA
TGATGAGCACTTTTAAAGTTCTGCTATGTGGCGCGGTATTATCCCGTATTGACGCCGGGCA
AGAGCAACTCGGTCGCCGCATACACTATTCTCAGAATGACTTGGTTGAGTACTCACCAGTC
ACAGAAAAGCATCTTACGGATGGCATGACAGTAAGAGAATTATGCAGTGCTGCCATAACCA
TGAGTGATAACACTGCGGCCAACTTACTTCTGACAACGATCGGAGGACCGAAGGAGCTAA
CCGCTTTTTTGCACAACATGGGGGATCATGTAACTCGCCTTGATCGTTGGGAACCGGAGC
TGAATGAAGCCATACCAAACGACGAGCGTGACACCACGATGCCTGTAGCAATGGCAACA
ACGTTGCGCAAACTATTAACTGGCGAACTACTTACTCTAGCTTCCCGGCAACAATTAATAGA
CTGGATGGAGGCGGATAAAGTTGCAGGACCACTTCTGCGCTCGGCCCTTCCGGCTGGCT
GGTTTATTGCTGATAAATCTGGAGCCGGTGAGCGTGGGTCTCGCGGTATCATTGCAGCACT
GGGGCCAGATGGTAAGCCCTCCCGTATCGTAGTTATCTACACGACGGGGAGTCAGGCAA
CTATGGATGAACGAAATAGACAGATCGCTGAGATAGGTGCCTCACTGATTAAGCATTGGTA
ACTGTCAGACCAAGTTTACTCATATATACTTTAGATTGATTTAAAACTTCATTTTTAATTTAAAAG
GATCTAGGTGAAGATCCTTTTTGATAATCTCATGACCAAAATCCCTTAACGTGAGTTTTCGTT
CCACTGAGCGTCAGACCCCGTAGAAAAGATCAAAGGATCTTCTTGAGATCCTTTTTTTCTG
CGCGTAATCTGCTGCTTGCAAACAAAAAAACCACCGCTACCAGCGGTGGTTTGTTTGCCG
GATCAAGAGCTACCAACTCTTTTTCCGAAGGTAACTGGCTTCAGCAGAGCGCAGATACCA
AATACTGTTCTTCTAGTGTAGCCGTAGTTAGGCCACCACTTCAAGAACTCTGTAGCACCGC
CTACATACCTCGCTCTGCTAATCCTGTTACCAGTGGCTGCTGCCAGTGGCGATAAGTCGT
GTCTTACCGGGTTGGACTCAAGACGATAGTTACCGGATAAGGCGCAGCGGTCGGGCTGA
ACGGGGGGTTCGTGCACACAGCCCAGCTTGGAGCGAACGACCTACACCGAACTGAGAT
ACCTACAGCGTGAGCTATGAGAAAGCGCCACGCTTCCCGAAGGGAGAAAGGCGGACAG
GTATCCGGTAAGCGGCAGGGTCGGAACAGGAGAGCGCACGAGGGAGCTTCCAGGGGG
AAACGCCTGGTATCTTTATAGTCCTGTCGGGTTTCGCCACCTCTGACTTGAGCGTCGATTTT
TGTGATGCTCGTCAGGGGGGCGGAGCCTATGGAAAAACGCCAGCAACGCGGCCTTTTTA
CGGTTCCTGGCCTTTTGCTGGCCTTTTGCTCACATGTTCTTTCCTGCGTTATCCCCTG

pCN34e

ATTCTGTGGATAACCGTATTACCGCCTTTGAGTGAGCTGGCGGCCGCTGCATGCCTGCAG
GTCGACTCTAGAGGATCCCCGGGTACCGAGCTCGAATTCACTGGCCGTCGTTTTACAACG
TCGTGACTGGGAAAACCCTGGCGTTACCCAACTTAATCGCCTTGCAGCACATCCCCCTTT
CGCCAGCTGGCGTAATAGCGAAGAGGCCCGCACCGATCGCCCTTCCCAACAGTTGCGC
AGCCTGAATGGCGAATGGCGCCTTTGCGGAAAGAGTTAATAAGTTAACAGAAGATGAACC
AAAACTAAATGGTTTAGCAGGAAACTTAGATAAAAAAATGAATCCAGAATTATATTCAGAACA
GGAACAGCAACAAGAACAACAAAAGAATCAAAAACGAGATAGAGGTATGCACTTATAGAA
CATGCATTTATGCCGAGAAAACTTATTGGTTGGAATGGGCTATGTGTTAGCTAACTTGTTAGC
GAGTTGGTTGGACTTGAATTGGGATTAATCCCAAGAAAGTACCAACTCAACAACACATAAA
GCCCTGTAGGTTCCGACCAATAAGGAAATTGGAATAAAGCAATAAAAGGAGTTGAAGAAAT
GAAATTCAGAGAAGCCTTTGAGAATTTTATAACAAGTAAGTATGTACTTGGTGTTTTAGTAGTT
TTAACTGTTTACCAGATAATACAAATGCTTAAATAAAAAAAGACTTGATCTGATTAGACCAAAT
CTTTTGATAGTGTTATATTAATAACAAAATAAAAAGGAGTCGCTCACGCCCTGACCAAAGTTT
GTGAACGACATCATTCAAAGAAAAAAACACTGAGTTGTTTTTATAATCTTGTATATTTAGATATT
AAACGATATTTAAATATACATCAAGATATATATTTGGGTGAGCGATTCCTTAAACGAAATTGAG
ATTAAGGAGTCGATTTTTTATGTATAAAAACAATCATGCAAATCATTCAAATCATTTGGAAAAT
CACGATTTAGACAATTTTTCTAAAACCGGCTACTCTAATAGCCGGTTGGACGCACATACTGT
GTGCATATCTGATCCAAAATTAAGTTTTGATGCAATGACGATCGTTGGAAATCTCAACCGAG
ACAACGCTCAAGCCCTTTCTAAATTTATGAGTGTAGAGCCCCAAATAAGACTTTGGGATATT
CTTCAAACAAAGTTTAAAGCTAAAGCACTTCAAGAAAAAGTTTATATTGAATATGACAAAGTG
AAAGCAGATAGTTGGGATAGACGTAATATGCGTATTGAATTTAATCCAAACAAACTTACACG
AGATGAAATGATTTGGTTAAAACAAAATATAATAAGCTACATGGAAGATGACGGTTTTACAAG
ATTAGATTTAGCCTTTGATTTTGAAGATGATTTGAGTGACTACTATGCAATGTCTGATAAAGCA
GTTAAGAAAACTATTTTTTATGGTCGTAATGGTAAGCCAGAAACAAAATATTTTGGCGTGAGA
GATAGTAATAGATTTATTAGAATTTATAATAAAAAGCAAGAACGTAAAGATAATGCAGATGCTG
AAGTTATGTCTGAACATTTATGGCGTGTAGAAATCGAACTTAAAAGAGATATGGTGGATTACT
GGAATGATTGCTTTAGTGATTTACATATCTTGCAACCAGATTGGAAAACTATCCAACGCACT
GCGGATAGAGCAATAGTTTTTATGTTATTGAGTGATGAAGAAGAATGGGGAAAGCTTCACAG
AAATTCTAGAACAAAATATAAGAATTTGATAAAAGAAATTTCGCCAGTCGATTTAACGGACTT
AATGAAATCGACTTTAAAAGCGAACGAAAAACAATTGCAAAAACAAATCGATTTTTGGCAAC
ATGAATTTAAATTTTGGAAATAGTGTACATATTAATATTACTGAACAAAAATGATATATTTAAACT
ATTCTAATTTAGGAGGATTTTTTTATGAAGTGTCTATTTAAAAATTTGGGGAATTTATATGAGGT
GAAAGAATAATTTACCCCTATAAACTTTAGTCACCTCAAGTAAAGAGGTAAAATTGTTTAGTTT
ATATAAAAAATTTAAAGGTTTGTTTTATAGCGTTTTATTTTGGCTTTGTATTCTTTCATTTTTTAGTG
TATTAAATGAAATGGTTTTAAATGTTTCTTTACCTGATATTGCAAATCATTTTAATACTACTCCTG
GAATTACAAACTGGGTAAACACTGCATATATGTTAACTTTTTCGATAGGAACAGCAGTATATG
GAAAATTATCTGATTATATAAATATAAAAAAAATTGTTAATTATTGGTATTAGTTTGAGCTGTCTTG
GTTCATTGATTGCTTTTATTGGGCCCACCTAGGAATTGAATGAGACATGCTACACCTCCGGA
TAATAAATATATATAAACGTATATAGATTTCATAAAGTCTAACACACTAGACTTATTTACTTCGT
AATTAAGTCGTTAAACCGTGTGCTCTACGACCAAAACTATAAAACCTTTAAGAACTTTCTTTTT
TTACAAGAAAAAAGAAATTAGATAAATCTCTCATATCTTTTATTCAATAATCGCATCCGATTGC
AGTATAAATTTAACGATCACTCATCATGTTCATATTTATCAGAGCTCGTGCTATAATTATACTA
ATTTTATAAGGAGGAAAAAATATGGGCATTTTTAGTATTTTTGTAATCAGCACAGTTCATTATCA
ACCAAACAAAAAATAAGTGGTTATAATGAATCGTTAATAAGCAAAATTCATATAACCAAATTAA
AGAGGGTTATAATGAACGAGAAAAATATAAAACACAGTCAAAACTTTATTACTTCAAAACATA
ATATAGATAAAATAATGACAAATATAAGATTAAATGAACATGATAATATCTTTGAAATCGGCTC

AGGAAAAGGCCATTTTACCCTTGAATTAGTAAAGAGGTGTAATTTCGTAACTGCCATTGAAAT
AGACCATAAATTATGCAAAACTACAGAAAATAAACTTGTTGATCACGATAATTTCCAAGTTTTA
AACAAGGATATATTGCAGTTTAAATTTCCTAAAAACCAATCCTATAAAATATATGGTAATATAC
CTTATAACATAAGTACGGATATAATACGCAAAATTGTTTTTGATAGTATAGCTAATGAGATTTAT
TTAATCGTGGAATACGGGTTTGCTAAAAGATTATTAAATACAAAACGCTCATTGGCATTACTTT
TAATGGCAGAAGTTGATATTTCTATATTAAGTATGGTTCCAAGAGAATATTTTCATCCTAAACC
TAAAGTGAATAGCTCACTTATCAGATTAAGTAGAAAAAAATCAAGAATATCACACAAAGATAA
ACAAAAGTATAATTATTTCGTTATGAAATGGGTTAACAAAGAATACAAGAAAATATTTACAAAA
AATCAATTTAACAATTCCTTAAAACATGCAGGAATTGACGATTTAAACAATATTAGCTTTGAAC
AATTCTTATCTCTTTTCAATAGCTATAAATTATTTAATAAGTAAGTTAAGGGATGCATAAACTGC
ATCCCTTAACTTGTTTTTCGTGTGCCTATTTTTTGTGAATCGATTATGTCTTTTGCGCAGTCGG
CTTAAACCAGTTTTCCGCGGCCTCGAGCGGCCGCATAGTTAAGCCAGCCCCGACACCC
GCCAACACCCGCTGACGCGCCCTGACGGGCTTGTCTGCTCCCGGCATCCGCTTACAGA
CAAGCTGTGACCGTCTCCGGGAGCTGCATGTGTCAGAGGTTTTCACCGTCATCACCGAAA
CGCGCGAGACGAAAGGGCCTCGTGATACGCCTATTTTTATAGGTTAATGTCATGATAATAAT
GGTTTCTTAGACGTCAGGTGGCACTTTTCGGGGAAATGTGCGCGGAACCCCTATTTGTTTAT
TTTTCTAAATACATTCAAATATGTATCCGCTCATGAGACAATAACCCTGATAAATGCTTCAATA
ATATTGAAAAAGGAAGAGTATGAGTATTCAACATTTCCGTGTCGCCCTTATTCCCTTTTTTGC
GGCATTTTGCCTTCCTGTTTTTGCTCACCCAGAAACGCTGGTGAAAGTAAAAGATGCTGAA
GATCAGTTGGGTGCACGAGTGGGTTACATCGAACTGGATCTCAACAGCGGTAAGATCCTT
GAGAGTTTTCGCCCCGAAGAACGTTTTCCAATGATGAGCACTTTTAAAGTTCTGCTATGTGG
CGCGGTATTATCCCGTATTGACGCCGGGCAAGAGCAACTCGGTCGCCGCATACACTATT
CTCAGAATGACTTGGTTGAGTACTCACCAGTCACAGAAAAGCATCTTACGGATGGCATGAC
AGTAAGAGAATTATGCAGTGCTGCCATAACCATGAGTGATAACACTGCGGCCAACTTACTT
CTGACAACGATCGGAGGACCGAAGGAGCTAACCGCTTTTTTGCACAACATGGGGGATCAT
GTAACTCGCCTTGATCGTTGGGAACCGGAGCTGAATGAAGCCATACCAAACGACGAGCG
TGACACCACGATGCCTGTAGCAATGGCAACAACGTTGCGCAAACTATTAACTGGCGAACT
ACTTACTCTAGCTTCCCGGCAACAATTAATAGACTGGATGGAGGCGGATAAAGTTGCAGGA
CCACTTCTGCGCTCGGCCCTTCCGGCTGGCTGGTTTATTGCTGATAAATCTGGAGCCGGT
GAGCGTGGGTCTCGCGGTATCATTGCAGCACTGGGGCCAGATGGTAAGCCCTCCCGTAT
CGTAGTTATCTACACGACGGGGAGTCAGGCAACTATGGATGAACGAAATAGACAGATCGC
TGAGATAGGTGCCTCACTGATTAAGCATTGGTAACTGTCAGACCAAGTTTACTCATATATACT
TTAGATTGATTTAAAACTTCATTTTTAATTTAAAAGGATCTAGGTGAAGATCCTTTTTGATAATCT
CATGACCAAAATCCCTTAACGTGAGTTTTCGTTCCACTGAGCGTCAGACCCCGTAGAAAAG
ATCAAAGGATCTTCTTGAGATCCTTTTTTTCTGCGCGTAATCTGCTGCTTGCAAACAAAAAAA
CCACCGCTACCAGCGGTGGTTTGTTTGCCGGATCAAGAGCTACCAACTCTTTTTCCGAAG
GTAACTGGCTTCAGCAGAGCGCAGATACCAAATACTGTTCTTCTAGTGTAGCCGTAGTTAG
GCCACCACTTCAAGAACTCTGTAGCACCGCCTACATACCTCGCTCTGCTAATCCTGTTAC
CAGTGGCTGCTGCCAGTGGCGATAAGTCGTGTCTTACCGGGTTGGACTCAAGACGATAGT
TACCGGATAAGGCGCAGCGGTCGGGCTGAACGGGGGGTTCGTGCACACAGCCCAGCTT
GGAGCGAACGACCTACACCGAACTGAGATACCTACAGCGTGAGCTATGAGAAAGCGCCA
CGCTTCCCGAAGGGAGAAAGGCGGACAGGTATCCGGTAAGCGGCAGGGTCGGAACAG
GAGAGCGCACGAGGGAGCTTCCAGGGGGAAACGCCTGGTATCTTTATAGTCCTGTCGGG
TTTCGCCACCTCTGACTTGAGCGTCGATTTTTGTGATGCTCGTCAGGGGGGCGGAGCCTA
TGGAAAAACGCCAGCAACGCGGCCTTTTTACGGTTCCTGGCCTTTTGCTGGCCTTTTGCTC
ACATGTTCTTTCCTGCGTTATCCCCTG

pCN34e *yfp*

ATTCTGTGGATAACCGTATTACCGCCTTTGAGTGAGCTGGCGGCCGCTGCATGCCTGCAG
GTCGACTCTAGAGGATCCGTAGCCCTTGCCTACCTAGCTTCCAAGAAAGATATCCTTACAG
CACAAGAGCGGAAAGATGTTTTGTTCTACATCCAGAACAACCTCTGCTAAAATTCCTGAAAA
ATTTTCGAAAAAGTTGTTGACTTTATCTACAAGGTGTGGCATAATGTGTGTGCATAATCAAAC
ACGAACGGGGGAACCAAAAGCTTAAGGAGGAAAGTCACATTATGAGCAAAGGTGAAGAA
CTGTTCACCGGCGTTGTGCCAATTCTGGTTGAGCTGGATGGTGACGTGAATGGCCACAAA
TTTTCCGTGTCTGGTGAAGGCGAGTGTGATGCTACTTATGGCAAACTGACTCTGAAACTGAT
CTGTACCACCGGCAAACTGCCTGTTCCGTGGCCAACTCTGGTCACTACTCTGGGTTACGG
CCTGATGTGTTTTGCGCGTTACCCGGATCACATGAAACAGCATGACTTCTTCAAATCTGCC
ATGCCGGAAGGCTATGTCCAAGAACGTACGATCTTTTTCAAGGACGACGGCAACTATAAAA
CCCGTGCCGAAGTTAAATTCGAGGGTGACACCCTGGTCAACCGCATCGAACTGAAAGGC
ATTGACTTCAAAGAGGACGGCAACATTCTGGGTCACAAGCTGGAATACAACTACAACTCC
CACAACGTTTACATTACTGCTGACAAGCAGAAAAACGGCATCAAAGCAAACTTCAAGATCC
GTCACAACATTGAAGATGGTGGCGTACAGCTGGCAGATCACTACCAGCAGAACACTCCAA
TCGGTGATGGCCCAGTACTGCTGCCAGATAACCATTACCTGTCCTACCAGAGCAAACTGT
CTAAAGACCCGAACGAAAAACGTGACCACATGGTACTGCTGGAATTTGTTACCGCGGCAG
GCATTACCCACGGTATGGACGAACTGTATAAATAAGCTAGCAAAAACCCCGCCCCTGACA
GGGCGGGGTTTTTTGAATTCACTGGCCGTCGTTTTACAACGTCGTGACTGGGAAAACCCT
GGCGTTACCCAACTTAATCGCCTTGCAGCACATCCCCCTTTCGCCAGCTGGCGTAATAGC
GAAGAGGCCCGCACCGATCGCCCTTCCCAACAGTTGCGCAGCCTGAATGGCGAATGGC
GCCTTTGCGGAAAGAGTTAATAAGTTAACAGAAGATGAACCAAAACTAAATGGTTTAGCAGG
AAACTTAGATAAAAAAATGAATCCAGAATTATATTCAGAACAGGAACAGCAACAAGAACAAC
AAAAGAATCAAAAACGAGATAGAGGTATGCACTTATAGAACATGCATTTATGCCGAGAAAAC
TTATTGGTTGGAATGGGCTATGTGTTAGCTAACTTGTTAGCGAGTTGGTTGGACTTGAATTGG
GATTAATCCCAAGAAAGTACCAACTCAACAACACATAAAGCCCTGTAGGTTCCGACCAATA
AGGAAATTGGAATAAAGCAATAAAAGGAGTTGAAGAAATGAAATTCAGAGAAGCCTTTGAGA
ATTTTATAACAAGTAAGTATGTACTTGGTGTTTTAGTAGTTTTAACTGTTTACCAGATAATACAA
ATGCTTAAATAAAAAAAGACTTGATCTGATTAGACCAAATCTTTTGATAGTGTTATATTAATAAC
AAAATAAAAAGGAGTCGCTCACGCCCTGACCAAAGTTTGTGAACGACATCATTCAAAGAAA
AAAACACTGAGTTGTTTTTATAATCTTGTATATTTAGATATTAAACGATATTTAAATATACATCAA
GATATATATTTGGGTGAGCGATTCCTTAAACGAAATTGAGATTAAGGAGTCGATTTTTTATGTA
TAAAAACAATCATGCAAATCATTCAAATCATTTGGAAAATCACGATTTAGACAATTTTTCTAAA
ACCGGCTACTCTAATAGCCGGTTGGACGCACATACTGTGTGCATATCTGATCCAAAATTAA
GTTTTGATGCAATGACGATCGTTGGAAATCTCAACCGAGACAACGCTCAAGCCCTTTCTAA
ATTTATGAGTGTAGAGCCCCAAATAAGACTTTGGGATATTCTTCAAACAAAGTTTAAAGCTAA
AGCACTTCAAGAAAAAGTTTATATTGAATATGACAAAGTGAAAGCAGATAGTTGGGATAGAC
GTAATATGCGTATTGAATTTAATCCAAACAAACTTACACGAGATGAAATGATTTGGTTAAAAC
AAAATATAATAAGCTACATGGAAGATGACGGTTTTACAAGATTAGATTTAGCCTTTGATTTTGA
AGATGATTTGAGTGACTACTATGCAATGTCTGATAAAGCAGTTAAGAAAACTATTTTTTATGGT
CGTAATGGTAAGCCAGAAACAAAATATTTTGGCGTGAGAGATAGTAATAGATTTATTAGAATT
TATAATAAAAAGCAAGAACGTAAAGATAATGCAGATGCTGAAGTTATGTCTGAACATTTATGG
CGTGTAGAAATCGAACTTAAAAGAGATATGGTGGATTACTGGAATGATTGCTTTAGTGATTTA
CATATCTTGCAACCAGATTGGAAAACTATCCAACGCACTGCGGATAGAGCAATAGTTTTTAT
GTTATTGAGTGATGAAGAAGAATGGGGAAAGCTTCACAGAAATTCTAGAACAAAATATAAGA
ATTTGATAAAAGAAATTTCGCCAGTCGATTTAACGGACTTAATGAAATCGACTTTAAAAGCGA
ACGAAAAACAATTGCAAAAACAAATCGATTTTTGGCAACATGAATTTAAATTTTGGAAATAGT

GTACATATTAATATTACTGAACAAAAATGATATATTTAAACTATTCTAATTTAGGAGGATTTTTTT
ATGAAGTGTCTATTTAAAAATTTGGGGAATTTATATGAGGTGAAAGAATAATTTACCCCTATAA
ACTTTAGTCACCTCAAGTAAAGAGGTAAAATTGTTTAGTTTATATAAAAAATTTAAAGGTTTGTT
TTATAGCGTTTTATTTTGGCTTTGTATTCTTTCATTTTTTAGTGTATTAAATGAAATGGTTTTAAAT
GTTTCTTTACCTGATATTGCAAATCATTTTAATACTACTCCTGGAATTACAAACTGGGTAAACA
CTGCATATATGTTAACTTTTTCGATAGGAACAGCAGTATATGGAAAATTATCTGATTATATAAAT
ATAAAAAAATTGTTAATTATTGGTATTAGTTTGAGCTGTCTTGGTTCATTGATTGCTTTTATTGGG
CCCACCTAGGAATTGAATGAGACATGCTACACCTCCGGATAATAAATATATATAAACGTATA
TAGATTTCATAAAGTCTAACACACTAGACTTATTTACTTCGTAATTAAGTCGTTAAACCGTGTG
CTCTACGACCAAAACTATAAAACCTTTAAGAACTTTCTTTTTTTACAAGAAAAAAGAAATTAGA
TAAATCTCTCATATCTTTTATTCAATAATCGCATCCGATTGCAGTATAAATTTAACGATCACTC
ATCATGTTCATATTTATCAGAGCTCGTGCTATAATTATACTAATTTTATAAGGAGGAAAAAATAT
GGGCATTTTTAGTATTTTTGTAATCAGCACAGTTCATTATCAACCAAACAAAAAATAAGTGGTT
ATAATGAATCGTTAATAAGCAAAATTCATATAACCAAATTAAAGAGGGTTATAATGAACGAGA
AAAATATAAAACACAGTCAAAACTTTATTACTTCAAAACATAATATAGATAAAATAATGACAAAT
ATAAGATTAAATGAACATGATAATATCTTTGAAATCGGCTCAGGAAAAGGCCATTTTACCCTT
GAATTAGTAAAGAGGTGTAATTTCGTAACTGCCATTGAAATAGACCATAAATTATGCAAAACT
ACAGAAAATAAACTTGTTGATCACGATAATTTCCAAGTTTTAAACAAGGATATATTGCAGTTTA
AATTTCCTAAAAACCAATCCTATAAAATATATGGTAATATACCTTATAACATAAGTACGGATAT
AATACGCAAAATTGTTTTTGATAGTATAGCTAATGAGATTTATTTAATCGTGGAATACGGGTTT
GCTAAAAGATTATTAAATACAAAACGCTCATTGGCATTACTTTTAATGGCAGAAGTTGATATTT
CTATATTAAGTATGGTTCCAAGAGAATATTTTCATCCTAAACCTAAAGTGAATAGCTCACTTAT
CAGATTAAGTAGAAAAAAATCAAGAATATCACACAAAGATAAACAAAAGTATAATTATTTCGTT
ATGAAATGGGTTAACAAAGAATACAAGAAAATATTTACAAAAAATCAATTTAACAATTCCTTAA
AACATGCAGGAATTGACGATTTAAACAATATTAGCTTTGAACAATTCTTATCTCTTTTCAATAG
CTATAAATTATTTAATAAGTAAGTTAAGGGATGCATAAACTGCATCCCTTAACTTGTTTTTCGT
GTGCCTATTTTTTGTGAATCGATTATGTCTTTTGCGCAGTCGGCTTAAACCAGTTTTCCGCGG
CCTCGAGCGGCCGCATAGTTAAGCCAGCCCCGACACCCGCCAACACCCGCTGACGCG
CCCTGACGGGCTTGTCTGCTCCCGGCATCCGCTTACAGACAAGCTGTGACCGTCTCCGG
GAGCTGCATGTGTCAGAGGTTTTCACCGTCATCACCGAAACGCGCGAGACGAAAGGGCC
TCGTGATACGCCTATTTTTATAGGTTAATGTCATGATAATAATGGTTTCTTAGACGTCAGGTGG
CACTTTTCGGGGAAATGTGCGCGGAACCCCTATTTGTTTATTTTTCTAAATACATTCAAATATG
TATCCGCTCATGAGACAATAACCCTGATAAATGCTTCAATAATATTGAAAAAGGAAGAGTAT
GAGTATTCAACATTTCCGTGTCGCCCTTATTCCCTTTTTTGCGGCATTTTGCCTTCCTGTTTTT
GCTCACCCAGAAACGCTGGTGAAAGTAAAAGATGCTGAAGATCAGTTGGGTGCACGAGTG
GGTTACATCGAACTGGATCTCAACAGCGGTAAGATCCTTGAGAGTTTTCGCCCCGAAGAA
CGTTTTCCAATGATGAGCACTTTTAAAGTTCTGCTATGTGGCGCGGTATTATCCCGTATTGAC
GCCGGGCAAGAGCAACTCGGTCGCCGCATACACTATTCTCAGAATGACTTGGTTGAGTAC
TCACCAGTCACAGAAAAGCATCTTACGGATGGCATGACAGTAAGAGAATTATGCAGTGCTG
CCATAACCATGAGTGATAACACTGCGGCCAACTTACTTCTGACAACGATCGGAGGACCGA
AGGAGCTAACCGCTTTTTTGCACAACATGGGGGATCATGTAACTCGCCTTGATCGTTGGGA
ACCGGAGCTGAATGAAGCCATACCAAACGACGAGCGTGACACCACGATGCCTGTAGCAA
TGGCAACAACGTTGCGCAAACTATTAACTGGCGAACTACTTACTCTAGCTTCCCGGCAACA
ATTAATAGACTGGATGGAGGCGGATAAAGTTGCAGGACCACTTCTGCGCTCGGCCCTTCC
GGCTGGCTGGTTTATTGCTGATAAATCTGGAGCCGGTGAGCGTGGGTCTCGCGGTATCATT
GCAGCACTGGGGCCAGATGGTAAGCCCTCCCGTATCGTAGTTATCTACACGACGGGGAG
TCAGGCAACTATGGATGAACGAAATAGACAGATCGCTGAGATAGGTGCCTCACTGATTAAG

CATTGGTAACTGTCAGACCAAGTTTACTCATATATACTTTAGATTGATTTAAAACTTCATTTTTA
ATTTAAAAGGATCTAGGTGAAGATCCTTTTTGATAATCTCATGACCAAAATCCCTTAACGTGA
GTTTTCGTTCCACTGAGCGTCAGACCCCGTAGAAAAGATCAAAGGATCTTCTTGAGATCCT
TTTTTTCTGCGCGTAATCTGCTGCTTGCAAACAAAAAAACCACCGCTACCAGCGGTGGTTT
GTTTGCCGGATCAAGAGCTACCAACTCTTTTTCCGAAGGTAACTGGCTTCAGCAGAGCGC
AGATACCAAATACTGTTCTTCTAGTGTAGCCGTAGTTAGGCCACCACTTCAAGAACTCTGTA
GCACCGCCTACATACCTCGCTCTGCTAATCCTGTTACCAGTGGCTGCTGCCAGTGGCGAT
AAGTCGTGTCTTACCGGGTTGGACTCAAGACGATAGTTACCGGATAAGGCGCAGCGGTC
GGGCTGAACGGGGGGTTCGTGCACACAGCCCAGCTTGGAGCGAACGACCTACACCGAA
CTGAGATACCTACAGCGTGAGCTATGAGAAAGCGCCACGCTTCCCGAAGGGAGAAAGGC
GGACAGGTATCCGGTAAGCGGCAGGGTCGGAACAGGAGAGCGCACGAGGGAGCTTCC
AGGGGGAAACGCCTGGTATCTTTATAGTCCTGTCGGGTTTCGCCACCTCTGACTTGAGCG
TCGATTTTTGTGATGCTCGTCAGGGGGGCGGAGCCTATGGAAAAACGCCAGCAACGCGG
CCTTTTTACGGTTCCTGGCCTTTTGCTGGCCTTTTGCTCACATGTTCTTTCCTGCGTTATCCC
CTG

pCN34e *ktrA-yfp*

ATTCTGTGGATAACCGTATTACCGCCTTTGAGTGAGCTGGCGGCCGCTGCATGCCTGCAG
GTCGACTCTAGAGGATCCGTAGCCCTTGCCTACCTAGCTTCCAAGAAAGATATCCTTACAG
CACAAGAGCGGAAAGATGTTTTGTTCTACATCCAGAACAACCTCTGCTAAAATTCCTGAAAA
ATTTTCGAAAAAGTTGTTGACTTTATCTACAAGGTGTGGCATAATGTGTGTGCATAATCAAAC
ACGAACGGGGGAACCAACGATTGGCTGTTTTATTAACAGCCTTGGGGTGAATCTTACTAAG
TAAGAGGGGGTACTCTGAATCCCTAATCCGACAGCTAACCTCGTAGGCGTATACAGAGAG
GAGGTCTATTATCGGCCATTCTTTGCAATGGTCTTTTTTGTTTCAAAAACGTTAATTCACTAAA
GCTTAAGGAGGAAAGTCACATTATGAGCAAAGGTGAAGAACTGTTCACCGGCGTTGTGCC
AATTCTGGTTGAGCTGGATGGTGACGTGAATGGCCACAAATTTTCCGTGTCTGGTGAAGGC
GAGTGTGATGCTACTTATGGCAAACTGACTCTGAAACTGATCTGTACCACCGGCAAACTGC
CTGTTCCGTGGCCAACTCTGGTCACTACTCTGGGTTACGGCCTGATGTGTTTTGCGCGTTA
CCCGGATCACATGAAACAGCATGACTTCTTCAAATCTGCCATGCCGGAAGGCTATGTCCA
AGAACGTACGATCTTTTTCAAGGACGACGGCAACTATAAAACCCGTGCCGAAGTTAAATTC
GAGGGTGACACCCTGGTCAACCGCATCGAACTGAAAGGCATTGACTTCAAAGAGGACGG
CAACATTCTGGGTCACAAGCTGGAATACAACTACAACTCCCACAACGTTTACATTACTGCT
GACAAGCAGAAAAACGGCATCAAAGCAAACTTCAAGATCCGTCACAACATTGAAGATGGT
GGCGTACAGCTGGCAGATCACTACCAGCAGAACACTCCAATCGGTGATGGCCCAGTACT
GCTGCCAGATAACCATTACCTGTCCTACCAGAGCAAACTGTCTAAAGACCCGAACGAAAA
ACGTGACCACATGGTACTGCTGGAATTTGTTACCGCGGCAGGCATTACCCACGGTATGGA
CGAACTGTATAAATAAGCTAGCAAAAACCCCGCCCCTGACAGGGCGGGGTTTTTTTGAATT
CACTGGCCGTCGTTTTACAACGTCGTGACTGGGAAAACCCTGGCGTTACCCAACTTAATC
GCCTTGCAGCACATCCCCCTTTCGCCAGCTGGCGTAATAGCGAAGAGGCCCGCACCGA
TCGCCCTTCCCAACAGTTGCGCAGCCTGAATGGCGAATGGCGCCTTTGCGGAAAGAGTT
AATAAGTTAACAGAAGATGAACCAAAACTAAATGGTTTAGCAGGAAACTTAGATAAAAAAAT
GAATCCAGAATTATATTCAGAACAGGAACAGCAACAAGAACAACAAAAGAATCAAAAACGA
GATAGAGGTATGCACTTATAGAACATGCATTTATGCCGAGAAAACTTATTGGTTGGAATGGG
CTATGTGTTAGCTAACTTGTTAGCGAGTTGGTTGGACTTGAATTGGGATTAATCCCAAGAAA
GTACCAACTCAACAACACATAAAGCCCTGTAGGTTCCGACCAATAAGGAAATTGGAATAAA
GCAATAAAAGGAGTTGAAGAAATGAAATTCAGAGAAGCCTTTGAGAATTTTATAACAAGTAA
GTATGTACTTGGTGTTTTAGTAGTTTTAACTGTTTACCAGATAATACAAATGCTTAAATAAAAAA
AGACTTGATCTGATTAGACCAAATCTTTTGATAGTGTTATATTAATAACAAAATAAAAAGGAGT
CGCTCACGCCCTGACCAAAGTTTGTGAACGACATCATTCAAAGAAAAAAACACTGAGTTGT
TTTTATAATCTTGTATATTTAGATATTAAACGATATTTAAATATACATCAAGATATATATTTGGGTG
AGCGATTCCTTAAACGAAATTGAGATTAAGGAGTCGATTTTTTATGTATAAAAACAATCATGC
AAATCATTCAAATCATTTGGAAAATCACGATTTAGACAATTTTTCTAAAACCGGCTACTCTAAT
AGCCGGTTGGACGCACATACTGTGTGCATATCTGATCCAAAATTAAGTTTTGATGCAATGAC
GATCGTTGGAAATCTCAACCGAGACAACGCTCAAGCCCTTTCTAAATTTATGAGTGTAGAG
CCCCAAATAAGACTTTGGGATATTCTTCAAACAAAGTTTAAAGCTAAAGCACTTCAAGAAAA
AGTTTATATTGAATATGACAAAGTGAAAGCAGATAGTTGGGATAGACGTAATATGCGTATTGA
ATTTAATCCAAACAAACTTACACGAGATGAAATGATTTGGTTAAAACAAAATATAATAAGCTAC
ATGGAAGATGACGGTTTTACAAGATTAGATTTAGCCTTTGATTTTGAAGATGATTTGAGTGACT
ACTATGCAATGTCTGATAAAGCAGTTAAGAAAACTATTTTTTATGGTCGTAATGGTAAGCCAG
AAACAAAATATTTTGGCGTGAGAGATAGTAATAGATTTATTAGAATTTATAATAAAAAGCAAGA
ACGTAAAGATAATGCAGATGCTGAAGTTATGTCTGAACATTTATGGCGTGTAGAAATCGAAC
TTAAAAGAGATATGGTGGATTACTGGAATGATTGCTTTAGTGATTTACATATCTTGCAACCAG
ATTGGAAAACTATCCAACGCACTGCGGATAGAGCAATAGTTTTTATGTTATTGAGTGATGAA

GAAGAATGGGGAAAGCTTCACAGAAATTCTAGAACAAAATATAAGAATTTGATAAAAGAAATT
TCGCCAGTCGATTTAACGGACTTAATGAAATCGACTTTAAAAGCGAACGAAAAACAATTGCA
AAAACAAATCGATTTTTGGCAACATGAATTTAAATTTTGGAAATAGTGTACATATTAATATTACT
GAACAAAAATGATATATTTAAACTATTCTAATTTAGGAGGATTTTTTTATGAAGTGTCTATTTAAA
AATTTGGGGAATTTATATGAGGTGAAAGAATAATTTACCCCTATAAACTTTAGTCACCTCAAG
TAAAGAGGTAAAATTGTTTAGTTTATATAAAAAAATTTAAAGGTTTGTTTTATAGCGTTTTATTTTGG
CTTTGTATTCTTTCATTTTTTAGTGTATTAAATGAAATGGTTTTAAATGTTTCTTTACCTGATATTG
CAAATCATTTTAATACTACTCCTGGAATTACAAACTGGGTAAACACTGCATATATGTTAACTTT
TTCGATAGGAACAGCAGTATATGGAAAATTATCTGATTATATAAATATAAAAAAATTGTTAATTA
TTGGTATTAGTTTGAGCTGTCTTGGTTCATTGATTGCTTTTATTGGGCCCACCTAGGAATTGAA
TGAGACATGCTACACCTCCGGATAATAAATATATATAAACGTATATAGATTTCATAAAGTCTAA
CACACTAGACTTATTTACTTCGTAATTAAGTCGTTAAACCGTGTGCTCTACGACCAAAACTAT
AAAACCTTTAAGAACTTTCTTTTTTTACAAGAAAAAAGAAATTAGATAAATCTCTCATATCTTTTA
TTCAATAATCGCATCCGATTGCAGTATAAATTTAACGATCACTCATCATGTTCATATTTATCAG
AGCTCGTGCTATAATTATACTAATTTTATAAGGAGGAAAAAATATGGGCATTTTTAGTATTTTTG
TAATCAGCACAGTTCATTATCAACCAAACAAAAAATAAGTGGTTATAATGAATCGTTAATAAG
CAAAATTCATATAACCAAATTAAAGAGGGTTATAATGAACGAGAAAAATATAAAACACAGTCA
AAACTTTATTACTTCAAAACATAATATAGATAAAATAATGACAAATATAAGATTAAATGAACATG
ATAATATCTTTGAAATCGGCTCAGGAAAAGGCCATTTTACCCTTGAATTAGTAAAGAGGTGTA
ATTTCGTAACTGCCATTGAAATAGACCATAAATTATGCAAAACTACAGAAAATAAACTTGTTG
ATCACGATAATTTCCAAGTTTTAAACAAGGATATATTGCAGTTTAAATTTCCTAAAAACCAATC
CTATAAAATATATGGTAATATACCTTATAACATAAGTACGGATATAATACGCAAAATTGTTTTTG
ATAGTATAGCTAATGAGATTTATTTAATCGTGGAATACGGGTTTGCTAAAAGATTATTAAATAC
AAAACGCTCATTGGCATTACTTTTAATGGCAGAAGTTGATATTTCTATATTAAGTATGGTTCCA
AGAGAATATTTTCATCCTAAACCTAAAGTGAATAGCTCACTTATCAGATTAAGTAGAAAAAAAT
CAAGAATATCACACAAAGATAAACAAAAGTATAATTATTTCGTTATGAAATGGGTTAACAAAG
AATACAAGAAAATATTTACAAAAAATCAATTTAACAATTCCTTAAAACATGCAGGAATTGACGA
TTTAAACAATATTAGCTTTGAACAATTCTTATCTCTTTTCAATAGCTATAAATTATTTAATAAGTAA
GTTAAGGGATGCATAAACTGCATCCCTTAACTTGTTTTTCGTGTGCCTATTTTTTGTGAATCGA
TTATGTCTTTTGCGCAGTCGGCTTAAACCAGTTTTCCGCGGCCTCGAGCGGCCGCATAGTT
AAGCCAGCCCCGACACCCGCCAACACCCGCTGACGCGCCCTGACGGGCTTGTCTGCT
CCCGGCATCCGCTTACAGACAAGCTGTGACCGTCTCCGGGAGCTGCATGTGTCAGAGGT
TTTCACCGTCATCACCGAAACGCGCGAGACGAAAGGGCCTCGTGATACGCCTATTTTTATA
GGTTAATGTCATGATAATAATGGTTTCTTAGACGTCAGGTGGCACTTTTCGGGGAAATGTGC
GCGGAACCCCTATTTGTTTATTTTTCTAAATACATTCAAATATGTATCCGCTCATGAGACAATA
ACCCTGATAAATGCTTCAATAATATTGAAAAAGGAAGAGTATGAGTATTCAACATTTCCGTGT
CGCCCTTATTCCCTTTTTTGCGGCATTTTGCCTTCCTGTTTTTGCTCACCCAGAAACGCTGG
TGAAAGTAAAAGATGCTGAAGATCAGTTGGGTGCACGAGTGGGTTACATCGAACTGGATCT
CAACAGCGGTAAGATCCTTGAGAGTTTTCGCCCCGAAGAACGTTTTCCAATGATGAGCACT
TTTAAAGTTCTGCTATGTGGCGCGGTATTATCCCGTATTGACGCCGGGCAAGAGCAACTCG
GTCGCCGCATACACTATTCTCAGAATGACTTGGTTGAGTACTCACCAGTCACAGAAAAGCA
TCTTACGGATGGCATGACAGTAAGAGAATTATGCAGTGCTGCCATAACCATGAGTGATAAC
ACTGCGGCCAACTTACTTCTGACAACGATCGGAGGACCGAAGGAGCTAACCGCTTTTTTG
CACAACATGGGGGATCATGTAACTCGCCTTGATCGTTGGGAACCGGAGCTGAATGAAGC
CATACCAAACGACGAGCGTGACACCACGATGCCTGTAGCAATGGCAACAACGTTGCGCA
AACTATTAACTGGCGAACTACTTACTCTAGCTTCCCGGCAACAATTAATAGACTGGATGGA
GGCGGATAAAGTTGCAGGACCACTTCTGCGCTCGGCCCTTCCGGCTGGCTGGTTTATTGC

TGATAAATCTGGAGCCGGTGAGCGTGGGTCTCGCGGTATCATTGCAGCACTGGGGCCAG
ATGGTAAGCCCTCCCGTATCGTAGTTATCTACACGACGGGGAGTCAGGCAACTATGGATG
AACGAAATAGACAGATCGCTGAGATAGGTGCCTCACTGATTAAGCATTGGTAACTGTCAGA
CCAAGTTTACTCATATATACTTTAGATTGATTTAAAACTTCATTTTTAATTTAAAAGGATCTAGGT
GAAGATCCTTTTTGATAATCTCATGACCAAAATCCCTTAACGTGAGTTTTCGTTCCACTGAGC
GTCAGACCCCGTAGAAAAGATCAAAGGATCTTCTTGAGATCCTTTTTTTCTGCGCGTAATCT
GCTGCTTGCAAACAAAAAAACCACCGCTACCAGCGGTGGTTTGTTTGCCGGATCAAGAGC
TACCAACTCTTTTTCCGAAGGTAACTGGCTTCAGCAGAGCGCAGATACCAAATACTGTTCTT
CTAGTGTAGCCGTAGTTAGGCCACCACTTCAAGAACTCTGTAGCACCGCCTACATACCTC
GCTCTGCTAATCCTGTTACCAGTGGCTGCTGCCAGTGGCGATAAGTCGTGTCTTACCGGG
TTGGACTCAAGACGATAGTTACCGGATAAGGCGCAGCGGTCGGGCTGAACGGGGGGTTC
GTGCACACAGCCCAGCTTGGAGCGAACGACCTACACCGAACTGAGATACCTACAGCGT
GAGCTATGAGAAAGCGCCACGCTTCCCGAAGGGAGAAAGGCGGACAGGTATCCGGTAA
GCGGCAGGGTCGGAACAGGAGAGCGCACGAGGGAGCTTCCAGGGGGAAACGCCTGGT
ATCTTTATAGTCCTGTCGGGTTTCGCCACCTCTGACTTGAGCGTCGATTTTTGTGATGCTCG
TCAGGGGGGCGGAGCCTATGGAAAAACGCCAGCAACGCGGCCTTTTTACGGTTCCTGGC
CTTTTGCTGGCCTTTTGCTCACATGTTCTTTCCTGCGTTATCCCCTG

pCN34e *kimA-yfp*

ATTCTGTGGATAACCGTATTACCGCCTTTGAGTGAGCTGGCGGCCGCTGCATGCCTGCAG
GTCGACTCTAGAGGATCCGTAGCCCTTGCCTACCTAGCTTCCAAGAAAGATATCCTTACAG
CACAAGAGCGGAAAGATGTTTTGTTCTACATCCAGAACAACCTCTGCTAAAATTCCTGAAAA
ATTTTCGAAAAAGTTGTTGACTTTATCTACAAGGTGTGGCATAATGTGTGTGCAGAAAACAAA
TCGCTTAATCTGAAATCAGAGCGGGGGACCCAATAGAACGGCTTTTTGCCGTTGGGGTGA
ATCCTTTTTAGGTAGGGCTAACTCTCATATGCCCGAATCCGTCAGCTAACCTCGTAAGCGT
TCGTGAGAGGAGATGAATGAAACCTGTGTTCGATGTTATGGCACAGGGGCATCCGTTTGC
CTCTGTGTTTTTTGTTGTTCATTTTTGAATGCAAATCGCATACAGAGACATCATAGCAGAAAG
GACTATCATCAACATGTGAGAGCAAGACACTGATGGATGTTTTATTGTTTTAATTGAAAAATG
AGAAATTAAAAGCTTAAGGAGGAAAGTCACATTATGAGCAAAGGTGAAGAACTGTTCACCG
GCGTTGTGCCAATTCTGGTTGAGCTGGATGGTGACGTGAATGGCCACAAATTTTCCGTGTC
TGGTGAAGGCGAGTGTGATGCTACTTATGGCAAACTGACTCTGAAACTGATCTGTACCACC
GGCAAACTGCCTGTTCCGTGGCCAACTCTGGTCACTACTCTGGGTTACGGCCTGATGTGT
TTTGCGCGTTACCCGGATCACATGAAACAGCATGACTTCTTCAAATCTGCCATGCCGGAAG
GCTATGTCCAAGAACGTACGATCTTTTTCAAGGACGACGGCAACTATAAAACCCGTGCCG
AAGTTAAATTCGAGGGTGACACCCTGGTCAACCGCATCGAACTGAAAGGCATTGACTTCA
AAGAGGACGGCAACATTCTGGGTCACAAGCTGGAATACAACTACAACTCCCACAACGTTT
ACATTACTGCTGACAAGCAGAAAAACGGCATCAAAGCAAACTTCAAGATCCGTCACAACAT
TGAAGATGGTGGCGTACAGCTGGCAGATCACTACCAGCAGAACACTCCAATCGGTGATG
GCCCAGTACTGCTGCCAGATAACCATTACCTGTCCTACCAGAGCAAACTGTCTAAAGACC
CGAACGAAAAACGTGACCACATGGTACTGCTGGAATTTGTTACCGCGGCAGGCATTACCC
ACGGTATGGACGAACTGTATAAATAAGCTAGCAAAAACCCCGCCCCTGACAGGGCGGGG
TTTTTTTGAATTCACTGGCCGTCGTTTTACAACGTCGTGACTGGGAAAACCCTGGCGTTACC
CAACTTAATCGCCTTGCAGCACATCCCCCTTTCGCCAGCTGGCGTAATAGCGAAGAGGC
CCGCACCGATCGCCCTTCCCAACAGTTGCGCAGCCTGAATGGCGAATGGCGCCTTTGC
GGAAAGAGTTAATAAGTTAACAGAAGATGAACCAAAACTAAATGGTTTAGCAGGAAACTTAG
ATAAAAAAATGAATCCAGAATTATATTCAGAACAGGAACAGCAACAAGAACAACAAAAGAAT
CAAAAACGAGATAGAGGTATGCACTTATAGAACATGCATTTATGCCGAGAAAACTTATTGGT
TGGAATGGGCTATGTGTTAGCTAACTTGTTAGCGAGTTGGTTGGACTTGAATTGGGATTAATC
CCAAGAAAGTACCAACTCAACAACACATAAAGCCCTGTAGGTTCCGACCAATAAGGAAATT
GGAATAAAGCAATAAAAGGAGTTGAAGAAATGAAATTCAGAGAAGCCTTTGAGAATTTTATAA
CAAGTAAGTATGTACTTGGTGTTTTAGTAGTTTTAACTGTTTACCAGATAATACAAATGCTTAAA
TAAAAAAAGACTTGATCTGATTAGACCAAATCTTTTGATAGTGTTATATTAATAACAAAATAAAA
AGGAGTCGCTCACGCCCTGACCAAAGTTTGTGAACGACATCATTCAAAGAAAAAAACACT
GAGTTGTTTTTATAATCTTGTATATTTAGATATTAAACGATATTTAAATATACATCAAGATATATAT
TTGGGTGAGCGATTCCTTAAACGAAATTGAGATTAAGGAGTCGATTTTTTATGTATAAAAACA
ATCATGCAAATCATTCAAATCATTTGGAAAATCACGATTTAGACAATTTTTCTAAAACCGGCTA
CTCTAATAGCCGGTTGGACGCACATACTGTGTGCATATCTGATCCAAAATTAAGTTTTGATG
CAATGACGATCGTTGGAAATCTCAACCGAGACAACGCTCAAGCCCTTTCTAAATTTATGAG
TGTAGAGCCCCAAATAAGACTTTGGGATATTCTTCAAACAAAGTTTAAAGCTAAAGCACTTC
AAGAAAAAGTTTATATTGAATATGACAAAGTGAAAGCAGATAGTTGGGATAGACGTAATATGC
GTATTGAATTTAATCCAAACAAACTTACACGAGATGAAATGATTTGGTTAAAACAAAATATAAT
AAGCTACATGGAAGATGACGGTTTTACAAGATTAGATTTAGCCTTTGATTTTGAAGATGATTTG
AGTGACTACTATGCAATGTCTGATAAAGCAGTTAAGAAAACTATTTTTTATGGTCGTAATGGTA
AGCCAGAAACAAAATATTTTGGCGTGAGAGATAGTAATAGATTTATTAGAATTTATAATAAAAA
GCAAGAACGTAAAGATAATGCAGATGCTGAAGTTATGTCTGAACATTTATGGCGTGTAGAAA

TCGAACTTAAAAGAGATATGGTGGATTACTGGAATGATTGCTTTAGTGATTTACATATCTTGCA
ACCAGATTGGAAAACTATCCAACGCACTGCGGATAGAGCAATAGTTTTTATGTTATTGAGTG
ATGAAGAAGAATGGGGAAAGCTTCACAGAAATTCTAGAACAAAATATAAGAATTTGATAAAA
GAAATTTCGCCAGTCGATTTAACGGACTTAATGAAATCGACTTTAAAAGCGAACGAAAAACA
ATTGCAAAAACAAATCGATTTTTGGCAACATGAATTTAAATTTTGGAAATAGTGTACATATTAAT
ATTACTGAACAAAAATGATATATTTAAACTATTCTAATTTAGGAGGATTTTTTTATGAAGTGTCTA
TTTAAAAATTTGGGGAATTTATATGAGGTGAAAGAATAATTTACCCCTATAAACTTTAGTCACC
TCAAGTAAAGAGGTAAAATTGTTTAGTTTATATAAAAAATTTAAAGGTTTGTTTTATAGCGTTTTA
TTTTGGCTTTGTATTCTTTCATTTTTTAGTGTATTAAATGAAATGGTTTTAAATGTTTCTTTACCTG
ATATTGCAAATCATTTTAATACTACTCCTGGAATTACAAACTGGGTAAACACTGCATATATGTT
AACTTTTTCGATAGGAACAGCAGTATATGGAAAATTATCTGATTATATAAATATAAAAAAATTGT
TAATTATTGGTATTAGTTTGAGCTGTCTTGGTTCATTGATTGCTTTTATTGGGCCCACCTAGGA
ATTGAATGAGACATGCTACACCTCCGGATAATAAATATATATAAACGTATATAGATTTCATAAA
GTCTAACACACTAGACTTATTTACTTCGTAATTAAGTCGTTAAACCGTGTGCTCTACGACCAA
AACTATAAAACCTTTAAGAACTTTCTTTTTTTACAAGAAAAAAGAAATTAGATAAATCTCTCATA
TCTTTTATTCAATAATCGCATCCGATTGCAGTATAAATTTAACGATCACTCATCATGTTCATATT
TATCAGAGCTCGTGCTATAATTATACTAATTTTATAAGGAGGAAAAAATATGGGCATTTTTAGT
ATTTTTGTAATCAGCACAGTTCATTATCAACCAAACAAAAAATAAGTGGTTATAATGAATCGTT
AATAAGCAAAATTCATATAACCAAATTAAAGAGGGTTATAATGAACGAGAAAAATATAAAACA
CAGTCAAAACTTTATTACTTCAAAACATAATATAGATAAAATAATGACAAATATAAGATTAAATG
AACATGATAATATCTTTGAAATCGGCTCAGGAAAAGGCCATTTTACCCTTGAATTAGTAAAGA
GGTGTAATTTCGTAACTGCCATTGAAATAGACCATAAATTATGCAAAACTACAGAAAATAAAC
TTGTTGATCACGATAATTTCCAAGTTTTAAACAAGGATATATTGCAGTTTAAATTTCCTAAAAAC
CAATCCTATAAAATATATGGTAATATACCTTATAACATAAGTACGGATATAATACGCAAAATTG
TTTTTGATAGTATAGCTAATGAGATTTATTTAATCGTGGAATACGGGTTTGCTAAAAGATTATTA
AATACAAAACGCTCATTGGCATTACTTTTAATGGCAGAAGTTGATATTTCTATATTAAGTATGG
TTCCAAGAGAATATTTTCATCCTAAACCTAAAGTGAATAGCTCACTTATCAGATTAAGTAGAA
AAAAATCAAGAATATCACACAAAGATAAACAAAAGTATAATTATTTCGTTATGAAATGGGTTAA
CAAAGAATACAAGAAAATATTTACAAAAAATCAATTTAACAATTCCTTAAAACATGCAGGAATT
GACGATTTAAACAATATTAGCTTTGAACAATTCTTATCTCTTTTCAATAGCTATAAATTATTTAAT
AAGTAAGTTAAGGGATGCATAAACTGCATCCCTTAACTTGTTTTTCGTGTGCCTATTTTTTGTG
AATCGATTATGTCTTTTGCGCAGTCGGCTTAAACCAGTTTTCCGCGGCCTCGAGCGGCCG
CATAGTTAAGCCAGCCCCGACACCCGCCAACACCCGCTGACGCGCCCTGACGGGCTT
GTCTGCTCCCGGCATCCGCTTACAGACAAGCTGTGACCGTCTCCGGGAGCTGCATGTGT
CAGAGGTTTTCACCGTCATCACCGAAACGCGCGAGACGAAAGGGCCTCGTGATACGCCT
ATTTTTATAGGTTAATGTCATGATAATAATGGTTTCTTAGACGTCAGGTGGCACTTTTCGGGGA
AATGTGCGCGGAACCCCTATTTGTTTATTTTTCTAAATACATTCAAATATGTATCCGCTCATGA
GACAATAACCCTGATAAATGCTTCAATAATATTGAAAAAGGAAGAGTATGAGTATTCAACATT
TCCGTGTCGCCCTTATTCCCTTTTTTGCGGCATTTTGCCTTCCTGTTTTTGCTCACCCAGAAA
CGCTGGTGAAAGTAAAAGATGCTGAAGATCAGTTGGGTGCACGAGTGGGTTACATCGAAC
TGGATCTCAACAGCGGTAAGATCCTTGAGAGTTTTCGCCCCGAAGAACGTTTTCCAATGAT
GAGCACTTTTAAAGTTCTGCTATGTGGCGCGGTATTATCCCGTATTGACGCCGGGCAAGAG
CAACTCGGTCGCCGCATACACTATTCTCAGAATGACTTGGTTGAGTACTCACCAGTCACAG
AAAAGCATCTTACGGATGGCATGACAGTAAGAGAATTATGCAGTGCTGCCATAACCATGAG
TGATAACACTGCGGCCAACTTACTTCTGACAACGATCGGAGGACCGAAGGAGCTAACCG
CTTTTTTGCACAACATGGGGGATCATGTAACTCGCCTTGATCGTTGGGAACCGGAGCTGAA
TGAAGCCATACCAAACGACGAGCGTGACACCACGATGCCTGTAGCAATGGCAACAACGT

TGCGCAAACTATTAACTGGCGAACTACTTACTCTAGCTTCCCGGCAACAATTAATAGACTG
GATGGAGGCGGATAAAGTTGCAGGACCACTTCTGCGCTCGGCCCTTCCGGCTGGCTGGT
TTATTGCTGATAAATCTGGAGCCGGTGAGCGTGGGTCTCGCGGTATCATTGCAGCACTGG
GGCCAGATGGTAAGCCCTCCCGTATCGTAGTTATCTACACGACGGGGAGTCAGGCAACT
ATGGATGAACGAAATAGACAGATCGCTGAGATAGGTGCCTCACTGATTAAGCATTGGTAAC
TGTCAGACCAAGTTTACTCATATATACTTTAGATTGATTTAAAACTTCATTTTTAATTTAAAAGGA
TCTAGGTGAAGATCCTTTTTGATAATCTCATGACCAAAATCCCTTAACGTGAGTTTTCGTTCC
ACTGAGCGTCAGACCCCGTAGAAAAGATCAAAGGATCTTCTTGAGATCCTTTTTTTCTGCG
CGTAATCTGCTGCTTGCAAACAAAAAAACCACCGCTACCAGCGGTGGTTTGTTTGCCGGAT
CAAGAGCTACCAACTCTTTTTCCGAAGGTAACTGGCTTCAGCAGAGCGCAGATACCAAATA
CTGTTCTTCTAGTGTAGCCGTAGTTAGGCCACCACTTCAAGAACTCTGTAGCACCGCCTAC
ATACCTCGCTCTGCTAATCCTGTTACCAGTGGCTGCTGCCAGTGGCGATAAGTCGTGTCTT
ACCGGGTTGGACTCAAGACGATAGTTACCGGATAAGGCGCAGCGGTCGGGCTGAACGG
GGGGTTCGTGCACACAGCCCAGCTTGGAGCGAACGACCTACACCGAACTGAGATACCTA
CAGCGTGAGCTATGAGAAAGCGCCACGCTTCCCGAAGGGAGAAAGGCGGACAGGTATC
CGGTAAGCGGCAGGGTCGGAACAGGAGAGCGCACGAGGGAGCTTCCAGGGGGAAACG
CCTGGTATCTTTATAGTCCTGTCGGGTTTCGCCACCTCTGACTTGAGCGTCGATTTTTGTGA
TGCTCGTCAGGGGGGCGGAGCCTATGGAAAAACGCCAGCAACGCGGCCTTTTTACGGTT
CCTGGCCTTTTGCTGGCCTTTTGCTCACATGTTCTTTCCTGCGTTATCCCCTG

References:

1. Boles BR, Thoendel M, Roth AJ, Horswill AR. 2010. Identification of Genes Involved in Polysaccharide-Independent Biofilm Formation. Plos One 5.
2. Corrigan RM, Abbott JC, Burhenne H, Kaever V, Gründling A. 2011. c-di-AMP Is a new second messenger in *Staphylococcus aureus* with a role in controlling cell size and envelope stress. Plos Pathogens 7.
3. Bose JL, Fey PD, Bayles KW. 2013. Genetic tools to enhance the study of gene function and regulation in *Staphylococcus aureus*. Appl Environ Microbiol 79:2218-24.
4. Bowman L, Zeden MS, Schuster CF, Kaever V, Gründling A. 2016. New insights into the cyclic di-adenosine monophosphate (c-di-AMP) degradation pathway and the requirement of the cyclic dinucleotide for acid stress resistance in *Staphylococcus aureus*. Journal of Biological Chemistry 291:26970-26986.
5. Corrigan RM, Bowman L, Willis AR, Kaever V, Gründling A. 2015. Cross-talk between two nucleotide-signaling pathways in *Staphylococcus aureus*. Journal of Biological Chemistry 290:5826-5839.
6. Zeden MS, Schuster CF, Bowman L, Zhong QY, Williams HD, Gründling A. 2018. Cyclic di-adenosine monophosphate (c-di-AMP) is required for osmotic regulation in *Staphylococcus aureus* but dispensable for viability in anaerobic conditions. Journal of Biological Chemistry 293:3180-3200.
7. Coe KA, Lee W, Stone MC, Komazin-Meredith G, Meredith TC, Grad YH, Walker S. 2019. Multi-strain Tn-Seq reveals common daptomycin resistance determinants in. Plos Pathogens 15.
8. Fey PD, Endres JL, Yajjala VK, Widhelm TJ, Boissy RJ, Bose JL, Bayles KW. 2013. A genetic resource for rapid and comprehensive phenotype screening of nonessential *Staphylococcus aureus* genes. mBio 4:e00537-12.
9. O'Toole GA, Kolter R. 1998. Initiation of biofilm formation in WCS365 proceeds via multiple, convergent signalling pathways:: a genetic analysis. Molecular Microbiology 28:449-461.
10. Bae T, Banger AK, Wallace A, Glass EM, Aslund F, Schneewind O, Missiakas DM. 2004. *Staphylococcus aureus* virulence genes identified by bursa aurealis mutagenesis and nematode killing. Proc Natl Acad Sci U S A 101:12312-7.
